# Photophysical Properties and DNA Binding of Two Intercalating Osmium Polypyridyl Complexes Showing Light-Switch Effects

**DOI:** 10.1021/acs.inorgchem.2c01231

**Published:** 2022-09-12

**Authors:** Mark Stitch, Rayhaan Z. Boota, Alannah S. Chalkley, Tony D. Keene, Jeremy C. Simpson, Paul A. Scattergood, Paul I. P. Elliott, Susan J Quinn

**Affiliations:** † School of Chemistry, University College Dublin, Dublin 4 D04 V1W8, Ireland; ‡ Department of Chemical Sciences, School of Applied Sciences University of Huddersfield, Queensgate, Huddersfield HD1 3DH, U.K.; § Cell Screening Laboratory, School of Biology and Environmental Science, University College Dublin, Dublin 4 D04 V1W8, Ireland

## Abstract

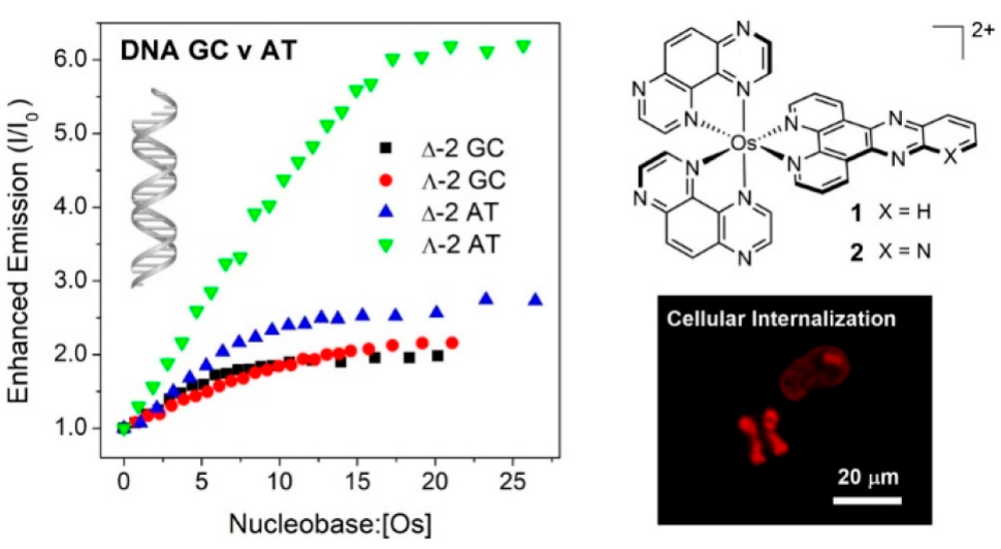

The synthesis and photophysical characterization of two osmium(II) polypyridyl complexes, [Os(TAP)_2_dppz]^2+^ (**1**) and [Os(TAP)_2_dppp2]^2+^ (**2**) containing dppz (dipyrido[3,2-*a*:2′,3′-*c*]phenazine) and dppp2 (pyrido[2′,3′:5,6]pyrazino[2,3-*f*][1,10]phenanthroline) intercalating ligands and TAP (1,4,5,8-tetraazaphenanthrene) ancillary ligands, are reported. The complexes exhibit complex electrochemistry with five distinct reductive redox couples, the first of which is assigned to a TAP-based process. The complexes emit in the near-IR (**1** at 761 nm and **2** at 740 nm) with lifetimes of >35 ns with a low quantum yield of luminescence in aqueous solution (∼0.25%). The Δ and Λ enantiomers of **1** and **2** are found to bind to natural DNA and with AT and GC oligodeoxynucleotides with high affinities. In the presence of natural DNA, the visible absorption spectra are found to display significant hypochromic shifts, which is strongly evident for the ligand-centered π–π* dppp2 transition at 355 nm, which undergoes 46% hypochromism. The emission of both complexes increases upon DNA binding, which is observed to be sensitive to the Δ or Λ enantiomer and the DNA composition. A striking result is the sensitivity of Λ-**2** to the presence of AT DNA, where a 6-fold enhancement of luminescence is observed and reflects the nature of the binding for the enantiomer and the protection from solution. Thermal denaturation studies show that both complexes are found to stabilize natural DNA. Finally, cellular studies show that the complexes are internalized by cultured mammalian cells and localize in the nucleus.

## Abstract 2

Osmium(II) polypyridyl complexes comprising extended dipyrido[3,2-*a*:2′,3′-*c*]phenazine (**1**) and pyrido[2′,3′:5,6]pyrazino[2,3-*f*][1,10]phenanthroline (**2**) intercalating ligands are shown to be effective DNA binders accompanied by enhanced near-IR emission. The emission response to B-DNA is found to be sensitive to the enantiomer and the composition of DNA, with greater emission observed for AT-rich sequences. Thermal denaturation studies show that both complexes stabilize natural DNA. Cellular studies show that the complexes are internalized by cultured mammalian cells and localize in the nucleus.

## Introduction

The excellent photophysical and electrochemical properties of transition-metal polypyridyl complexes make them attractive candidates for imaging and phototherapeutic applications.^1^ Ruthenium polypyridyl systems have been extensively studied by exploiting their ability to tune these properties through the careful choice of ligands to yield robust and water-soluble complexes.^1−6^ Recently, the potential of the diverse coordination chemistry and rich redox chemistry and photochemistry of osmium polypyridyl complexes has attracted renewed attention, in part related to their extended emission in the near-IR (NIR) region, which makes them attractive probes for biological studies.^7−14^ Early studies of osmium(II) polypyridyl complexes noted their tunable photophysical and redox properties, photostability, and ability to sensitize singlet oxygen (^1^O_2_) formation through energy transfer.^15−17^ However, while osmium complexes have the advantage of gaining access to the therapeutic window for in vivo applications, their electronic properties present some challenges. First, the greater electron count of the Os center compared to the Ru ion is expected to provide a less photooxidizing agent, and, second, the lifetimes of osmium(II) polypyridyl complexes are found to be shorter than those of their ruthenium(II) counterparts, which is attributed to energy gap law considerations, leading to more rapid nonradiative decay.^18^ In spite of this, a number of studies have revealed their potential as imaging and therapeutic agents and as attractive systems for DNA targeting.

A particularly attractive property of ruthenium(II) polypyridyl systems is their light-switch behavior, which has been extensively studied.^19−26^ Related to this, the DNA-intercalating Os(phen)_2_dppz^2+^ (phen = 1,10-phenanthroline; dppz = dipyridophenazine) also exhibits light-switch behavior, with significant emission enhancement observed in the presence of DNA.^27^ Notably the emission at ca. 730 nm is significantly red-shifted from that observed for the isostructural Ru(phen)_2_dppz^2+^ complex (ca. 617 nm).^21^ Furthermore, the Δ enantiomer, Δ-Os(phen)_2_dppz^2+^, is found to exhibit greater luminescent enhancement than the Λ form in the presence of natural DNA, which mirrors the observation for the ruthenium(II)-based system.^23,28^ Studies by the Barton group have demonstrated the ability to manipulate the oxidation potential of the metal-centered process (3^+^/2^+^) by incorporating electron-donating or -withdrawing substituents on the ancillary ligands, while substituents on the phenazine portion of the dppz ligand only affect the ligand reduction potential and not the metal-centered oxidation potential.^29^ Dinuclear osmium(II) bipyridine complexes comprising a pyrenylbiimidazole-based bridge have also been reported to show emission enhancement upon DNA binding attributed to decreasing the vibrational mode of relaxation.^30^ The application of the light-switching behavior has also been applied to cellular imaging, and the [Os(phen)_2_(dppz)]^2+^ complex has been used for correlative luminescence and transmission electron microscopy imaging of nuclear DNA.^31,32^ The Thomas group has also developed DNA-targeting dinuclear osmium(II) complexes as high-resolution contrast probes for cellular imaging using electron microscopy offering a safer alternative to the highly toxic OsO_4_,^13^ while in related work, targeted cellular imaging has been achieved using peptide-labeled osmium polypyridyl complexes.^9^ In a recent development, the 1,4,5,8-tetraazaphenanthrene (TAP)-based dinuclear osmium(II) was found to be stable to the high photon fluxes necessary for stimulated emission depletion (STED) microscopy and suitable for use in super-resolution NIR STED.^14^

Osmium polypyridyl complexes are also of interest for potential therapeutic applications.^10,12,33−36^ An early example of the DNA-targeting potential was reported by the Mesmaeker group, who demonstrated the ability of the Os(TAP)_3_^2+^ complex to photooxidize guanine by a direct single-electron-transfer mechanism. In contrast, the structurally similar Os(phen)_3_^2+^ was incapable of guanine photooxidation.^34^ However, the excited state of Os(TAP)_3_^2+^ is less oxidizing (*E*_red_* = +1.11 V vs SCE) than that of its Ru(TAP)_3_^2+^ (*E*_red_* = +1.35 V vs SCE) equivalent. DNA photodamage has also been achieved by ^1^O_2_ sensitization by the triplet state formed by photoexcitation of a Os(bpy)_2_dppn^2+^ (dppn = benzo[*i*]dipyrido-[3,2-*a*:2,3-*c*]phenazine) complex containing the extended dppn ligand, leading to DNA photocleavage in the photodynamic therapy (PDT) window.^35^ McFarland and co-workers have recently reported the potent PDT application of an osmium(II) analogue of their successful ruthenium-based PDT agent TLD-1433.^36^ In an exciting development, an accompanying study nicely profiled the intracellular photophysics of the system.^12^ The use of NIR imaging to gain insight into the mechanism of cytotoxicity was nicely demonstrated by the Keyes group, who reported cellular localization of an osmium(II) bis(4′-(4-carboxyphenyl)-2,2′:6′,2″-terpyridine) complex labeled with mitochondrial-targeting peptide sequences.^10^ No phototoxicity was detected, but critically the NIR imaging provided information on the distribution-dependent cytotoxicity.

We are interested in developing transition-metal DNA polypyridyl probes for imaging and therapeutics. The Quinn group recently explored the binding interactions of [Ru(phen)_2_dppz]^2+^ with a quadruplex and i-motif DNA and have extensively studied the photooxidation of guanine by the intercalating [Ru(TAP)_2_dppz]^2+^ complex for a number of DNA systems in both solution and crystals.^6,37,38^ In an exciting development, we recently revealed the role of a ligand-centered (^1^LC) excited state, and not the previously thought doublet metal-centered state, in the oxidation of adenine and guanine by the intercalated [Cr(TMP)_2_dppz]^2+^ complex (TMP = tetramethylphenanthroline).^39^ Work by the Scattergood and Elliott group has previously investigated triazole-containing osmium(II) complexes that show lysosomal/endosomal and mitochondrial localization as optical probes^7,8^ and as antimicrobial agents suitable for super-resolution imaging.^40^ The group has also recently investigated the photophysical properties and complex photochemistry exhibited by TAP complexes of ruthenium(II) featuring bitriazolyl ligands.^41^

**Figure 1:**
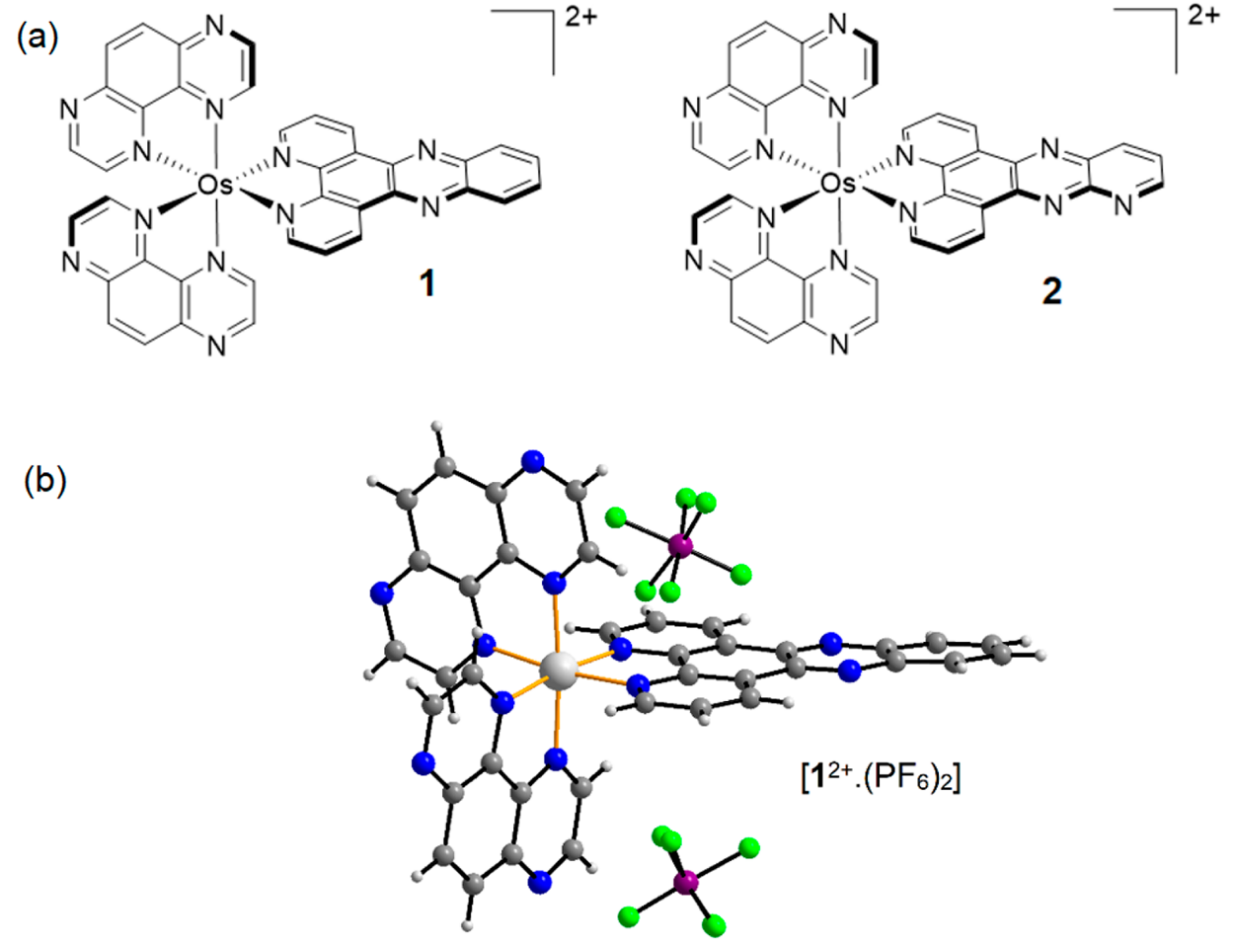
(a) Schematics of complexes **1** and **2**. (b) Crystal structure of complex **1**.

Now in this study we report the synthesis and photophysics of Os-dppz complexes incorporating the TAP ligand and consider the DNA binding properties of the resolved enantiomers (Figure 1). The Turro group previously observed that the exchange of dppz in Ru(bpy)_2_dppz^2+^, by the structurally related pyrido[2′,3′:5,6]pyrazino[2,3-*f*][1,10]phenanthroline (dppp2) ligand, results is a strong solvent-dependent emission, which has potential for sensing applications.^42,43^ Motivated, in part, by this work, we have performed a comparative study of the [Os(TAP)_2_(dppz)]^2+^ and [Os(TAP)_2_(dppp2)]^2+^ complexes to assess their potential as imaging and phototherapeutic agents. We used density functional theory (DFT) and time-dependent DFT (TDDFT) calculations to aid our understanding of the photophysical properties in solution and a DNA environment. Importantly, our results reveal the influence of both the enantiomer and the nature of the intercalating ligand (dppz or dppp2) on the photophysical response to the presence of DNA and on the ability to stabilize the DNA structure. Finally, we examine the cellular uptake and toxicity of the complexes.

## Results

### Synthesis and Chiral Resolution

[Os(TAP)_2_(dppz)][PF_6_]_2_ [**1^2+^**·(PF_6_)_2_] and [Os(TAP)_2_(dppp2)][PF_6_]_2_ [**2^2+^**·(PF_6_)_2_] were prepared via a two-step procedure (Scheme S1). Briefly, [OsCl_6_][NH_4_]_2_ was combined with 2 equiv of TAP in refluxing ethylene glycol to form the dichloride precursor complex [Os(TAP)_2_(Cl)_2_]. Further reaction with a stoichiometric amount of dppz or dppp2 followed by counterion metathesis with NH_4_PF_6_ yielded the hexafluorophosphate salts of **1^2+^** and **2^2+^** as dark-brown solids, whose identities were confirmed by NMR spectroscopy and mass spectrometry (Figures S1–S6). The enantiomers were resolved by passing through a C25 Sephadex column eluted with a (−)-*O*,*O*′-dibenzoyl-l-tartrate mobile phase (Figure S7). The structure of compound **1** was confirmed by single-crystal X-ray diffraction (Figure 1; the full structure description is given in the Supporting Information and in the packing diagram in Figure S8). The Os^2+^ oxidation state was determined by bond-valence-sum (BVS) analysis,^44^ having derived the appropriate *r*_0_ value empirically from a survey of the Cambridge Structural Database^45^ for Os^2/3+^ with N_6_ coordination spheres. Osmium N_6_ complexes show the unusual feature of increasing their bond length upon oxidation, which complicates the use of the BVS model. However, the use of the different *r*_0_ parameters for Os^2+^/Os^3+^ will return the correct oxidation state if used with care. Full details are given in the Excel file in the Supporting Information and Table S1.

### Electrochemical Studies

[**1^2+^**][PF_6_]_2_ was analyzed by cyclic voltammetry (Figure S9 and Table 1), revealing a fully reversible oxidation process at +0.95 V (vs Fc^+^/Fc) attributed to the osmium(II/III) couple. The reductive electrochemistry is more complex, displaying five distinct redox couples between −1.1 and −2.2 V. The most anodic process, centered at −1.11 V, is assigned to a TAP-based reduction, indicating that the lowest unoccupied molecular orbital (LUMO) is likely localized on the TAP ligands. The second and third reduction waves are closely spaced and electrochemically reversible, assigned with the aid of DFT calculations (*vide infra*) to reduction of the dppz moiety and a second TAP-based process, respectively. The two most cathodically shifted couples are found to be quasi-reversible and tentatively assigned to further reduction processes associated with the TAP ligands, again in good agreement with computational calculations. A somewhat more complex behavior is observed for [**2^2+^**][Cl]_2_, where, in addition to the electrochemically reversible osmium(II/III) couple, an irreversible oxidation step is observed that, because of the absence of this process in **1^2+^**, is likely to be associated with the terminal pyridyl unit on the dppp2 ligand (Figure S10).

**Table 1:** Summarized Electrochemical Data Recorded for 1.5 mmol dm^–3^ Room Temperature MeCN Solutions at 100 mV s^–1^^a^

	*E*_ox_/V	*E*_red_/V
**1**	+0.95 (73)	–1.11 (63), −1.32 (68), −1.43 (79), −1.92 (146)^b^, −2.18 (142)^b^
**2**	+0.71 (anodic peak), +0.97 (63)	–1.15 (110), −1.42 (86), −1.88 (113), −2.18 (140), −2.53 (171)

a The potentials are quoted relative to Fc^+^/Fc. The anodic–cathodic peak separations are shown in millivolts within brackets. Δ*E*_a,c_ for Fc^+^/Fc was typically 70 mV.

b Quasi-reversible.

The presence of the more electron-withdrawing TAP ligands results in a notable shift of both the osmium(II/III)- and ligand-based redox potentials toward more positive values compared to known bpy- or phen-based analogues, which is consistent with the relative oxidizing power of the two ligands.^46,47^

### Photophysical Studies

**Figure 2:**
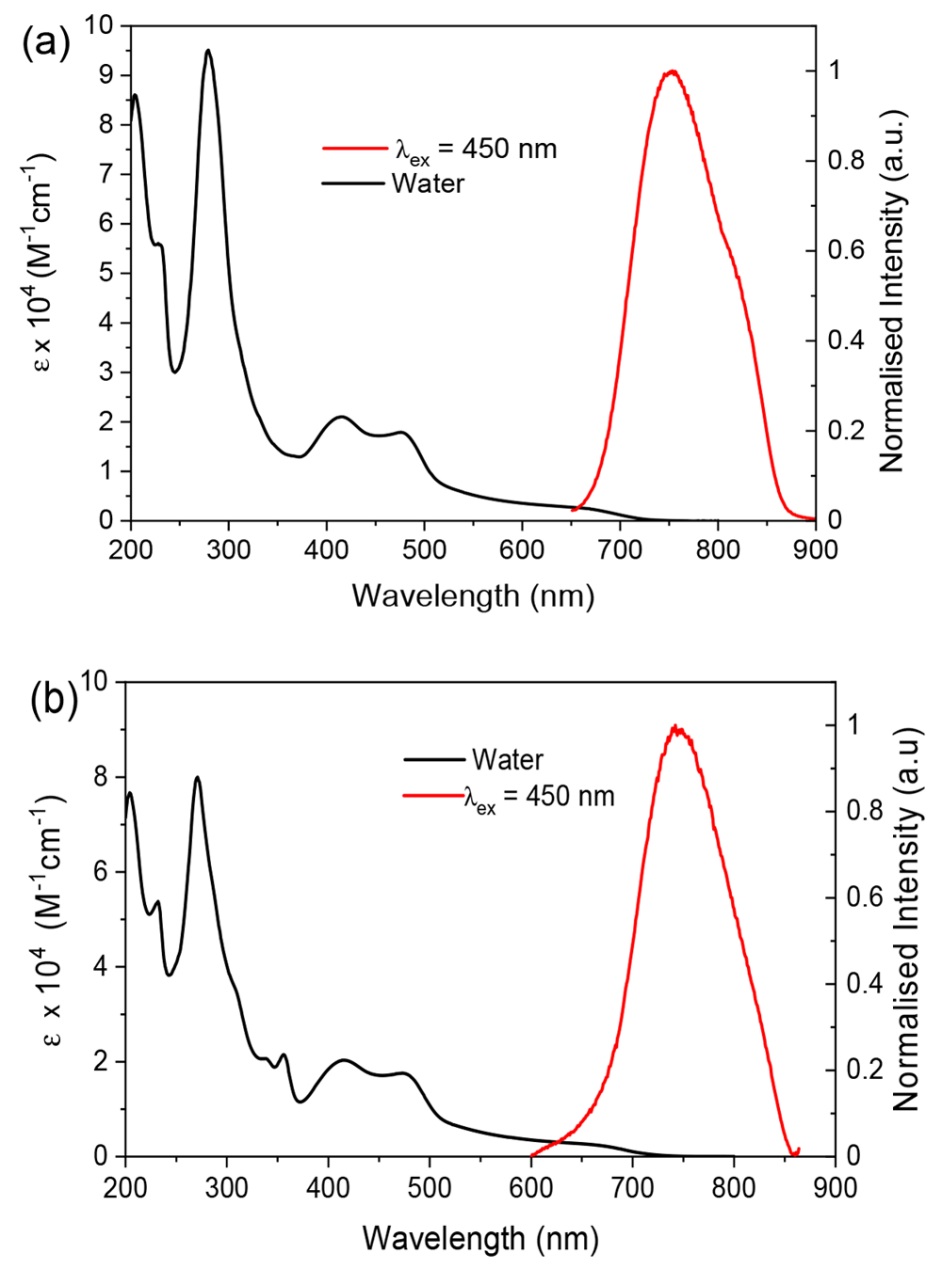
UV–visible electronic absorption and normalized photoluminescence spectra (λ_ex_ = 450 nm) recorded for aerated aqueous solutions of (a) [**1^2+^**][Cl]_2_ and (b) [**2^2+^**][Cl]_2_.

Metathesis of the counterions yielded the chloride salts of **1^2+^** and **2^2+^**, which were found to display excellent aqueous solubility. The UV–visible electronic absorption spectra of the complexes were recorded in aqueous solution (Figure 2). Sharp and intense transitions observed between 250 and 300 nm are assigned to singlet ^1^LC excitations associated with the TAP and dppz moieties; in the case of **2**, an additional absorbance band is observed at 355 nm due to a ^1^LC dppp2 transition. The broad absorption envelope between 370 and 520 nm is attributed to metal-to-ligand charge-transfer transitions of singlet character (^1^MLCT) from the Os center to both the TAP and dppz ligands. Such moderately intense absorbances in the visible region are commonly observed for osmium(II) polypyridyl systems, with ^1^MLCT transitions for Os(bpy)_3_^2+^, for example, falling between 430 and 520 nm.^48−50^ Further absorbances, still of appreciable intensity, are noted beyond 570 nm, which extend into the red region before tailing off at 720 nm. This feature is typical for osmium(II) coordination complexes^49,8,51−54^ and arises due to the formally spin-forbidden direct population of triplet states with MLCT character (^3^MLCT) as a consequence of the high spin–orbit coupling constant of the Os center.^48^ The circular dichroism (CD) spectra for the Δ and Λ stereoisomers of [**1^2+^**][Cl]_2_ and [**2^2+^**][Cl]_2_ show opposite (but equal) Cotton effects, with characteristic couplets observed for the ^1^LC and ^3^MLCT transitions, with the latter extending to the NIR region (Figures S11 and S12).

Both complexes are photoluminescent in aerated aqueous solution (Figure 2 and Table 2) and display a broad, featureless band lying within the deep-red/NIR region (λ_em_ = 761 nm for [**1**^**2+**^][Cl]_2_ and 740 nm for [**2**^**2+**^][Cl]_2_) assigned to the emission from a ^3^MLCT state. The quantum yields of luminescence [Φ_em_ = 0.29% (**1**) and 0.24% (**2**)] are low and consistent with the energy gap law, which predicts large nonradiative contributions to excited-state decay for low-energy states typically observed for osmium(II) complexes. As a result, a short luminescence lifetime of τ = 38 ns is observed for **1**. Notably, the luminescence of **2** exhibits a multiexponential decay, which in addition to a 37 ns (10%) component has significant contributions from a longer-lived species, 122 ns (36%) and 843 ns (54%). This longer-lived component may indicate some contribution from an ^3^LC state. This is in contrast to the monoexponential decay observed for dppz complexes of the type Os(bpy)_2_dppz-R. In both cases, saturation of the aqueous solution with N_2_ leads to only very slight changes in the quantum yield of luminescence, with the lifetime remaining similarly unaffected, although both complexes show increased luminescence in an acetonitrile (MeCN) solution (Figure S13). The luminescence of **1^2+^** and **2^2+^** was also examined in a frozen solution at 77 K (Figure S14) and display structured emission profiles that are blue-shifted (λ_em_= 717 and 713 nm, respectively) relative to those obtained in a fluid aqueous solution as a result of rigidochromic effects.

**Table 2:** Summarized Photophysical Data for **1^2+^** and **2^2+^**

	λ_abs_/nm (ε/mol^–1^ dm^3^ cm^–1^)^a^	λ_em_/nm^a^^,^^b^	λ_em_/nm^c^^,^^d^	Φ_em_/%^a^^,^^e^	Φ_em_/%^e^^,^^f^	τ/ns^a^	τ/ns^f^
**1**	665 (2450), 477 (17800), 414 (20,965), 279 (95155), 230 (54380)	761	717, 785 (sh)	0.29	0.33	38	38
**2**	665 (2400), 473 (17600), 415 (20300), 356 (21500), 271 (80000), 232 (53800)	740	713, 781 (sh)	0.24	0.23	37 (10%), 122 (36%), 843 (54%)	41 (10%), 131 (34%), 1073 (56%)

a Aerated aqueous solution.

b λ_ex_ = 500 nm.

c 4:1 ethanol/MeOH, 77 K.

d λ_ex_ = 480 nm.

e Relative to [Ru(bpy)_3_][PF_6_]_2_ in aerated MeCN; Φ_em_ = 1.8%.

f N_2_-equilibrated aqueous solution.

It has previously been observed that organic buffers can quench the emission of TAP-containing complexes of the form [Ru(bpy)_*n*_(TAP)_3*−n*_]^2+^ (*n* = 0–2).^55^ Interestingly, the emission of both complexes was observed to decrease under conditions of low pH achieved using organic buffers and also using hydrochloric acid (HCl). The absence of any change in the absorbance spectra under these conditions suggests that this is due to dynamic rather than static quenching effects (Figures S15 and S16 and Table S2).

### Computational Studies

The geometries of [Os(TAP)_2_(dppz)]^2+^ and [Os(TAP)_2_(dppp2)]^2+^ were optimized at the B3LYP level of theory with the def2-SD effective core potential and def2/j auxiliary basis set for the Os center and def2-svp basis sets for all other atoms (Tables S3 and S4). The COSMO-SMD model (MeCN) was applied for the optimization.

**Figure 3:**
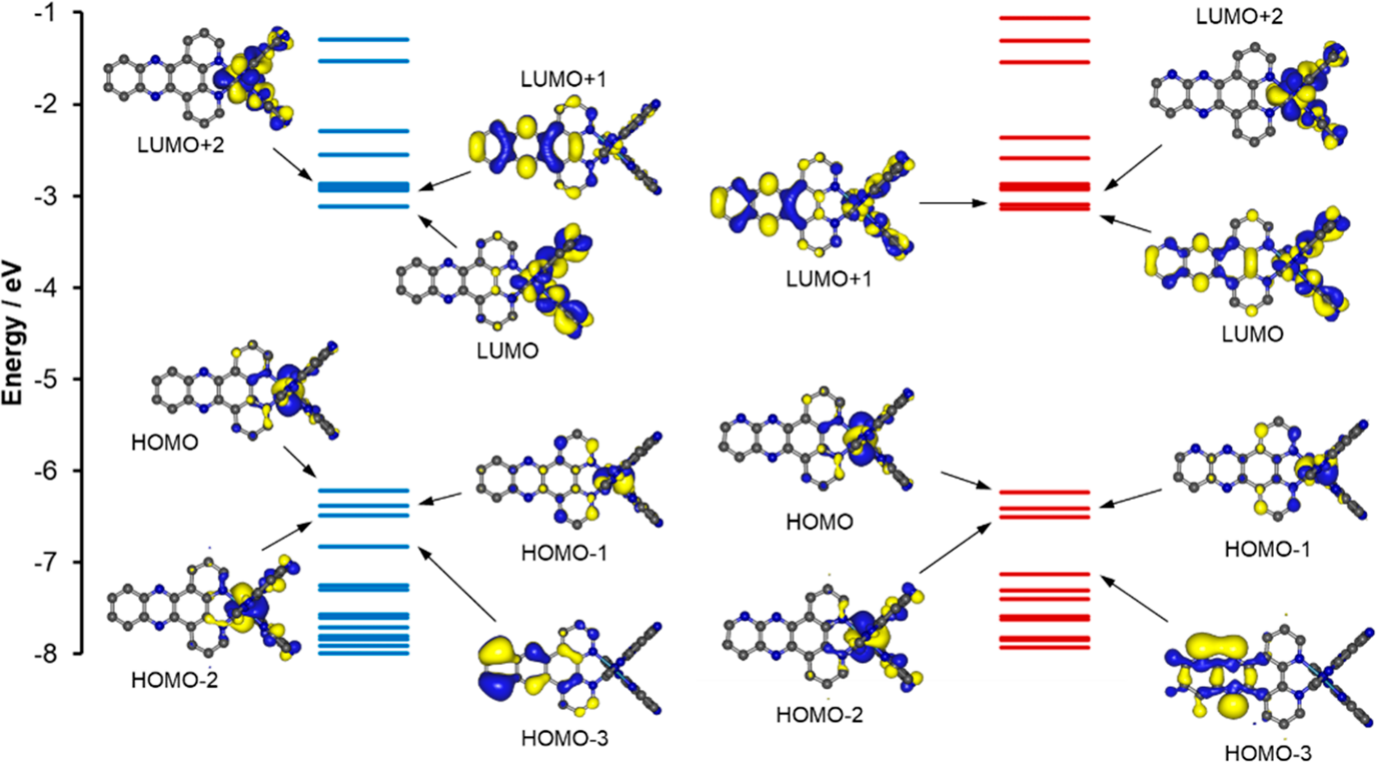
Plots of the energies for the frontier molecular orbitals for [Os(TAP)_2_(dppz)]^2+^ (blue) and [Os(TAP)_2_(dppp2)]^2+^ (red), with plots of HOMO–3 to LUMO+2 in both cases (isosurfaces set at 0.02 au). H atoms have been omitted for clarity.

The highest occupied molecular orbital (HOMO), HOMO–1 and HOMO–2 for both complexes are of predominantly Os d-orbital character with some additional contributions from the π systems of the ligand, in particular a contribution from the TAP ligands for HOMO–2. The higher-lying unoccupied orbitals have ligand-based π* character (Figure 3). In both cases, HOMO–3 is localized on the dppz or dppp2 ligand with π character for dppz; however, HOMO–3 primarily has lone-pair character for the noncoordinated N atoms for dppp2. The comparable lone-pair orbital for **1**^**2+**^ appears as HOMO–5 (Figure S17).

The LUMOs in both cases have significant contributions from the TAP ligands with a minor contribution from dppz for **1**^**2+**^ and a larger dppp2 contribution for **2**^**2+**^. LUMO+2, LUMO+3, and LUMO+4 are also predominantly localized on the TAP ligands in both cases. LUMO+1, on the other hand, is localized largely on the dppz ligand for **1**^**2+**^ and mostly on the phenazine portion of the ligand, while a similar localization of LUMO+1 is observed for **2**^**2+**^ but with additional TAP contributions. LUMO+5 and LUMO+6 are primarily localized on the dppz and dppp2 ligands. While there are slight changes to the compositions of the frontier orbitals for the two complexes, replacing dppz with the dppp2 ligand has only a minimal effect on their energies.

The UV–visible absorption spectra of [Os(TAP)_2_(dppz)]^2+^ and [Os(TAP)_2_(dppp2)]^2+^ in MeCN (COSMO-SMD) were calculated by TDDFT calculations and are in reasonable agreement with the experimental spectra; however, the energies of the absorption features are overestimated. In general, the lower-energy spin-allowed absorptions are dominated by TAP-localized ^1^MLCT transitions, with dppz-centered ^1^MLCT transitions appearing at higher energies (Figure S18). This pattern is repeated for the spin-forbidden ground state to ^3^MLCT state transitions at wavelengths longer than 500 nm. While the T_1_ transition for **1**^**2+**^ is of TAP-localized ^3^MLCT character, the T_1_ to T_3_ state transitions for **2**^**2+**^ are predominantly of HOMO–3 to LUMO, LUMO+1, LUMO+2, and LUMO+4 ligand-to-ligand (dppp2 to TAP) n → π* character. However, because these are formally spin-forbidden transitions, our TDDFT calculations do not provide relative oscillator strengths, and so the significance of the contribution to the experimental spectra cannot be judged. Because the spectral profiles of the two complexes are very similar in this region, this contribution may be inferred to be very small.

The lowest-lying triplet states of the two complexes were also optimized by U-DFT (MeCN). Examination of the singly occupied natural orbitals and localization of the spin densities reveal that the relaxed T_1_ states are dppz- and dppp2-based with π → π* character for **1**^**2+**^ and n → π* character for **2**^**2+**^ (Figure S19). This may explain the longer-lifetime components observed in the emission decay for **2**^**2+**^.

### DNA Binding Studies in Mixed-Sequence Natural DNA

#### UV–Visible Absorption

**Figure 4:**
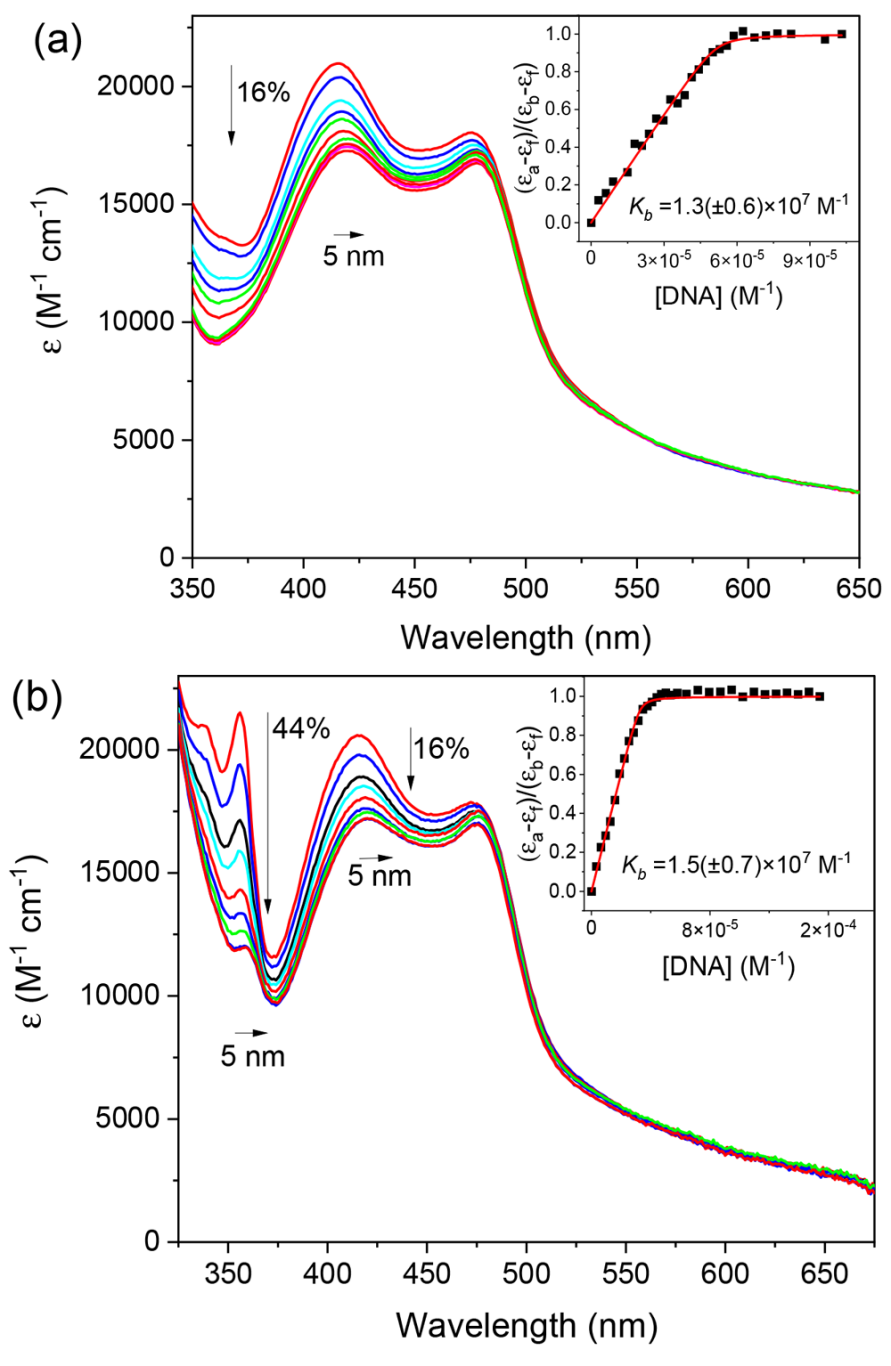
UV–visible absorbance spectra of (a) Δ-**1^2+^** (11.2 μM) and (b) Δ-**2^2+^** (12.2 μM) titrated against increasing concentrations of st-DNA (0 → 0.24 mM) in a 20 mM phosphate buffer at pH 7.0.

UV–visible titrations were performed to study the affinity of the enantiomers of **1** and **2** to salmon testes DNA (st-DNA). The addition of increasing DNA to Δ-**1** resulted in pronounced hypochromism at 415 nm associated with the Os^II^ dπ → π* MLCT dppz transition (16% reduction in intensity), which was accompanied by a slight red shift of ∼5 nm, with weaker hypochromism of the 465 nm band Os^II^ dπ → π* MLCT TAP transition (Figure 4a). These observations are characteristic of intercalation.^38^ The hypochromism at 415 nm was slightly lower (12%) in the case of Λ-**1**, which likely reflects a different binding interaction (Figure S20a). In the case of **2**, the well-resolved π–π* LC dppp2 transition at 355 nm allows direct reporting of the effects of intercalation on the ligand, which undergoes dramatic hypochromism (44%) for both Δ-**2** and Λ-**2** with a 5–7 nm red shift in the band position (Figures 4b and S20b). The DNA binding constants, *K*_b_, for the enantiomers of **1** and **2** were determined using the fitting model developed by Bard et al. (Table S5).^56^ In the case of **2**, the binding affinity was determined by monitoring the changes at both 355 and 415 nm, which yielded binding constants in excellent agreement, confirming that these bands are reporting on the same binding event (Table S5). As expected, the isostructural complexes were found to have a similar and strong affinity for st-DNA, with both enantiomers having *K*_b_ > 10^6^ M^–1^, and in both cases, the Δ enantiomer was found to bind more strongly.

#### Luminescence

**Figure 5:**
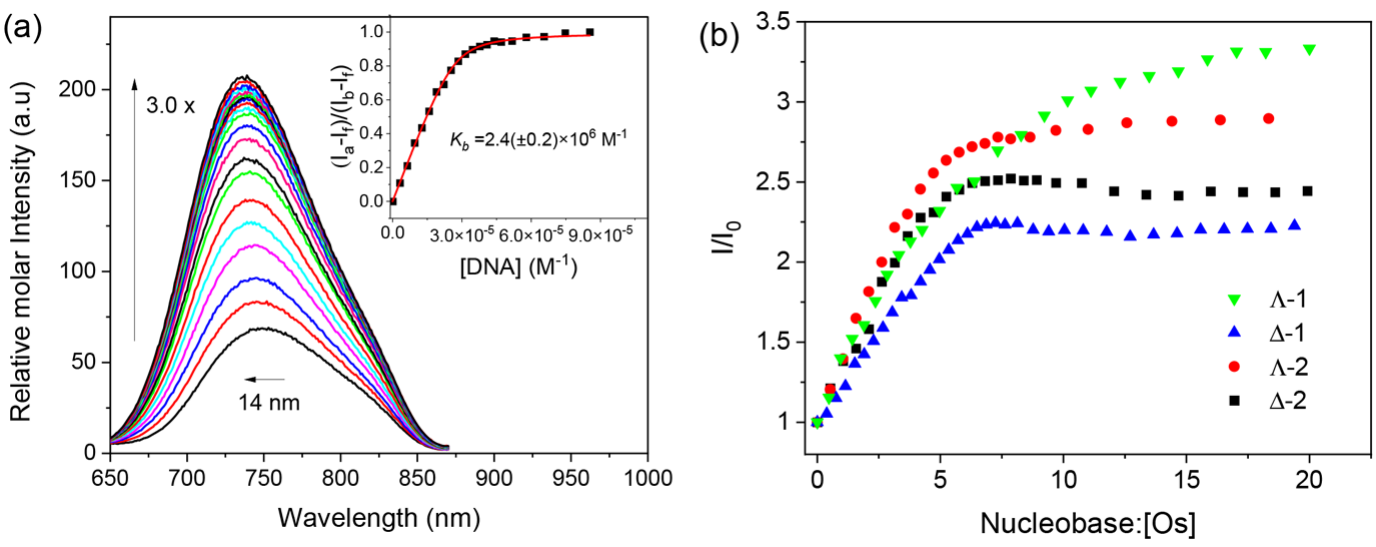
(a) Luminescence spectra of Λ-**2** (9.34 μM) titrated against increasing concentrations of st-DNA (0 → 0.24 mM). (b) Trend in luminescence enhancement upon increasing concentrations of st-DNA in the presence of enantiomers in a 20 mM phosphate buffer at pH 7.0. λ_ex_ = 465 nm.

The emission of **1** and **2** is enhanced upon increasing additions of natural DNA. Different degrees of enhancement were observed for the enantiomers: Λ-**1** (3.6) > Λ-**2** (3.0) > Δ-**2** (2.6) > Δ-**1** (2.0) (Figures 5 and S21). The emission enhancement in deaerated solutions (*I*_DNA_/*I*_0_) was found to be between 2 and 3.6. These differences are attributed to (i) differences in the sensitivity of the excited states of **1** and **2** to the environment and (ii) differences in the binding geometry of the two enantiomers. The Bard fitting model was again applied to determine binding constants from the luminescence data (Table 3).^56^ While greater enhancement was observed for the Λ enantiomers, the *K*_b_ values obtained by analysis of the emission changes indicated stronger binding for the Δ enantiomers, which is in good agreement with those calculated for the absorbance data (Table S5). The binding site size (*n*) ∼ 2 for the enantiomers is also similar to previously reported complexes in the literature.^57^ In the case of the Λ enantiomer, the emission profile is biphasic and continues to increase after this point. This increase is also coupled with a subtle hyperchromic shift in the absorption spectra, suggesting an additional binding mode at greater DNA concentrations.

**Table 3:** DNA Binding Constants (*K*_b_) and Binding Site Sizes (*s*) Determined for Enantiomers of **1** and **2** Using the Bard Treatment of the Emission Data at 750 nm for DNA Systems Titrated with a 20 mM Phosphate Buffer at pH 7.0

Λ	binding constant *K*_b_ (M^–1^)/binding site size (*s*)	Δ	binding constant *K*_b_ (M^–1^)/binding site size (*s*)
Λ-**1**/ST	1.6 (±0.4) × 10^6^ M^–1^/2.6 (±0.1)	Δ-**1**/ST	1.7 (±0.6) × 10^7^ M^–1^/1.8 (±0.02)
Λ-**1**/GC	2.0 (±0.4) × 10^6^ M^–1^/6.0 (±0.2)	Δ-**1**/GC	1.4 (±0.2) × 10^6^ M^–1^/2.7 (±0.1)
Λ-**1**/AT	1.1 (±0.3) × 10^7^ M^–1^ /5.0 (±0.1)	Δ-**1**/AT	8.5 (±0.5) × 10^6^ M^–1^/3.8 (±0.1)
Λ-**2**/ST	2.4 (±0.2) × 10^6^ M^–1^/1.5 (±0.02)	Δ-**2**/ST	1.3 (±0.7) × 10^7^ M^–1^/1.3 (±0.03)
Λ-**2**/GC	3.2 (±0.6) × 10^6^ M^–1^ /6.0 (±0.07)	Δ-**2**/GC	1.8 (±0.4) × 10^6^ M^–1^/3.4 (±0.1)
Λ-**2**/AT	1.4 (±0.4) × 10^7^ M^–1^/7.7 (±0.08)	Δ-**2**/AT	4.5 (±1.3) × 10^6^ M^–1^/4.6 (±0.1)

### DNA Binding Studies in GC and AT Oligodeoxynucleotides

We next investigated whether binding to a GC site or an AT site may influence the affinity of binding and the resulting luminescence enhancement. This was achieved by studying the behavior of **1** and **2** in the presence of the AT and GC self-complementary oligodeoxynucleotide sequences 5′-GCGCGCGCGC-3′ (GC) and 5′-ATATATATATAT-3′ (AT). The UV–visible spectra show changes similar to those observed in the presence of natural DNA (Figures S22 and S23). For both complexes, a modest red shift in the absorbance is accompanied by significant hypochromism of the MLCT band (11–15%), while for **2**, dramatic changes in the π–π* LC dppp2 transition at 355 nm were again observed (35–39%). These changes were also reflected in the binding constants obtained (Table S6).

**Figure 6:**
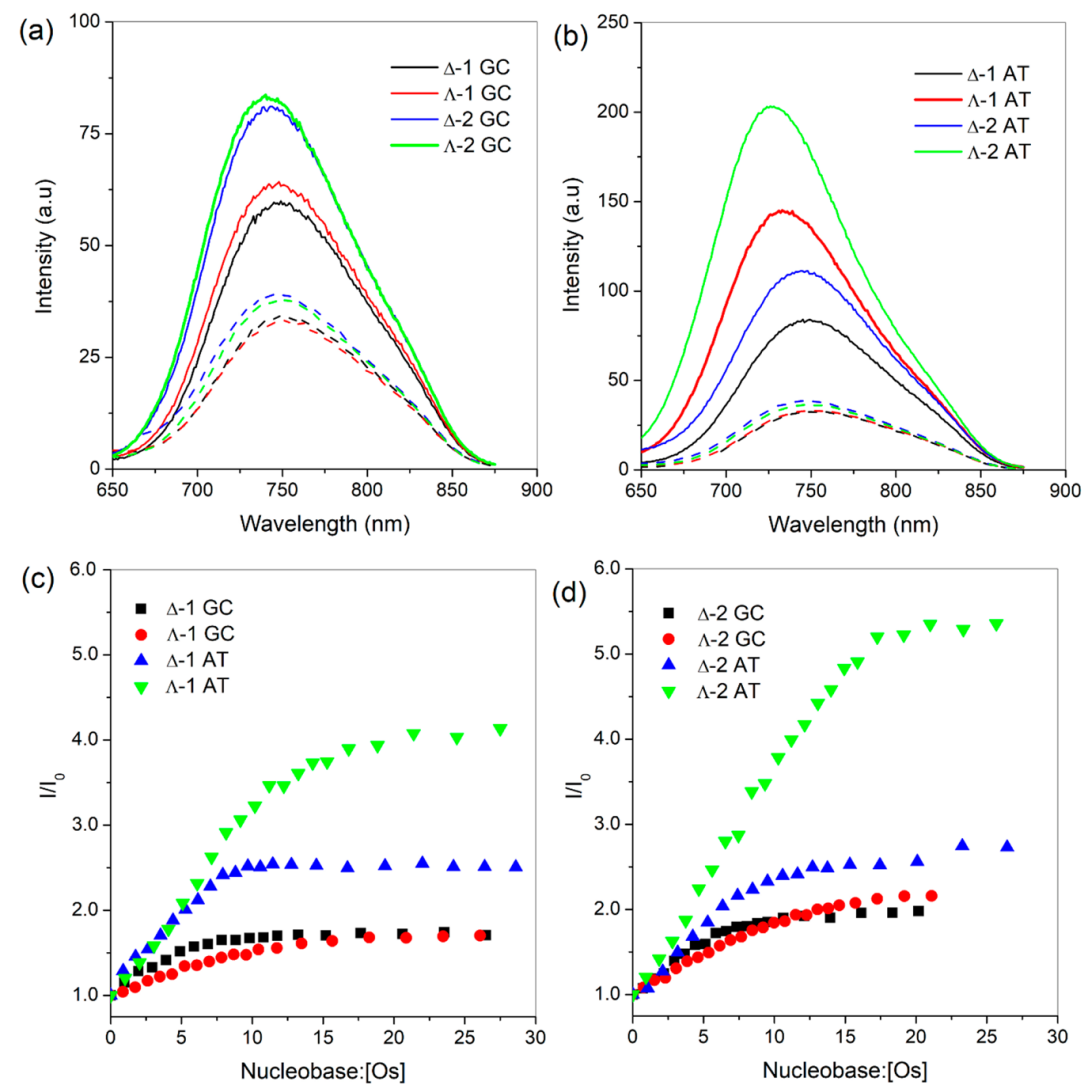
Change in the relative molar luminescence enhancement of **1** and **2** bound to (a) GC and (b) AT DNA ([nucleobase]:[Os] = 20; the initial relative molar luminescence of the complex without DNA is indicated by dashed lines). Trends in the luminescence enhancement observed for enantiomers of (c) **1** and (d) **2** upon increasing GC and AT, where the emission was recorded in a 20 mM phosphate buffer at pH 7.0. λ_ex_ = 465 nm.

In contrast, the emission spectra revealed greater sensitivity of the complex emission to the base composition (Figures 6 and S24–S26). In the case of both complexes, modest enhancement of the emission was observed in the presence of GC DNA, comparable to the enhancement observed for natural DNA, with greater enhancement observed for **2** (Figure 6a and Table S7). However, significantly greater enhancement was observed when both complexes were bound to AT DNA (Figure 6a–d). Strikingly, the enhancement of Λ-**2**^**2+**^ showed very distinct behavior compared to that of Δ-**2**^**2+**^ and also Δ-**1**^**2+**^ and Λ-**1**^**2+**^, displaying a 5.4-fold enhancement in the presence of AT DNA. This indicates the enhanced sensitivity of this complex to the DNA binding environment. A further indication of the different binding site environments is the shift in the position of the emission maximum. This blue shift was only seen for the Λ enantiomer of each complex when bound to the AT DNA sequence, with Λ-**2** exhibiting a slightly greater blue shift (25 nm from 750 to 725 nm) than Λ-**1**^**2+**^ (20 nm from 752 to 732 nm). In all titrations, the Λ enantiomers were observed to undergo greater enhancement in the emission (Figure 6). The binding constants obtained from the emission titrations are shown in Table 3 and compared to those from the absorbance titrations in Table S6.

Given the sensitivity of the emission enhancement observed by Λ-**2** in the presence of AT DNA, it was decided to investigate whether the complex displayed a preference for 5′-TA-3′ over 5′-AT-3′ in the model sequence (5′-CCGGXXCCGG)_2_.^58,59^ The results of the titrations of Λ-**2** with the model sequences are shown in Figures S27–S29 and Table S8. Significantly, a 3.8-fold enhancement of the emission was observed for Λ-**2** in the presence of (5′-CCGGTACCGG)_2_, suggesting binding to the central 5′-TA-3′ step. In contrast, a 2.3-fold enhancement was observed in the presence of (5′-CCGGATCCGG)_2_.

### CD and Linear Dichroism (LD)

**Figure 7:**
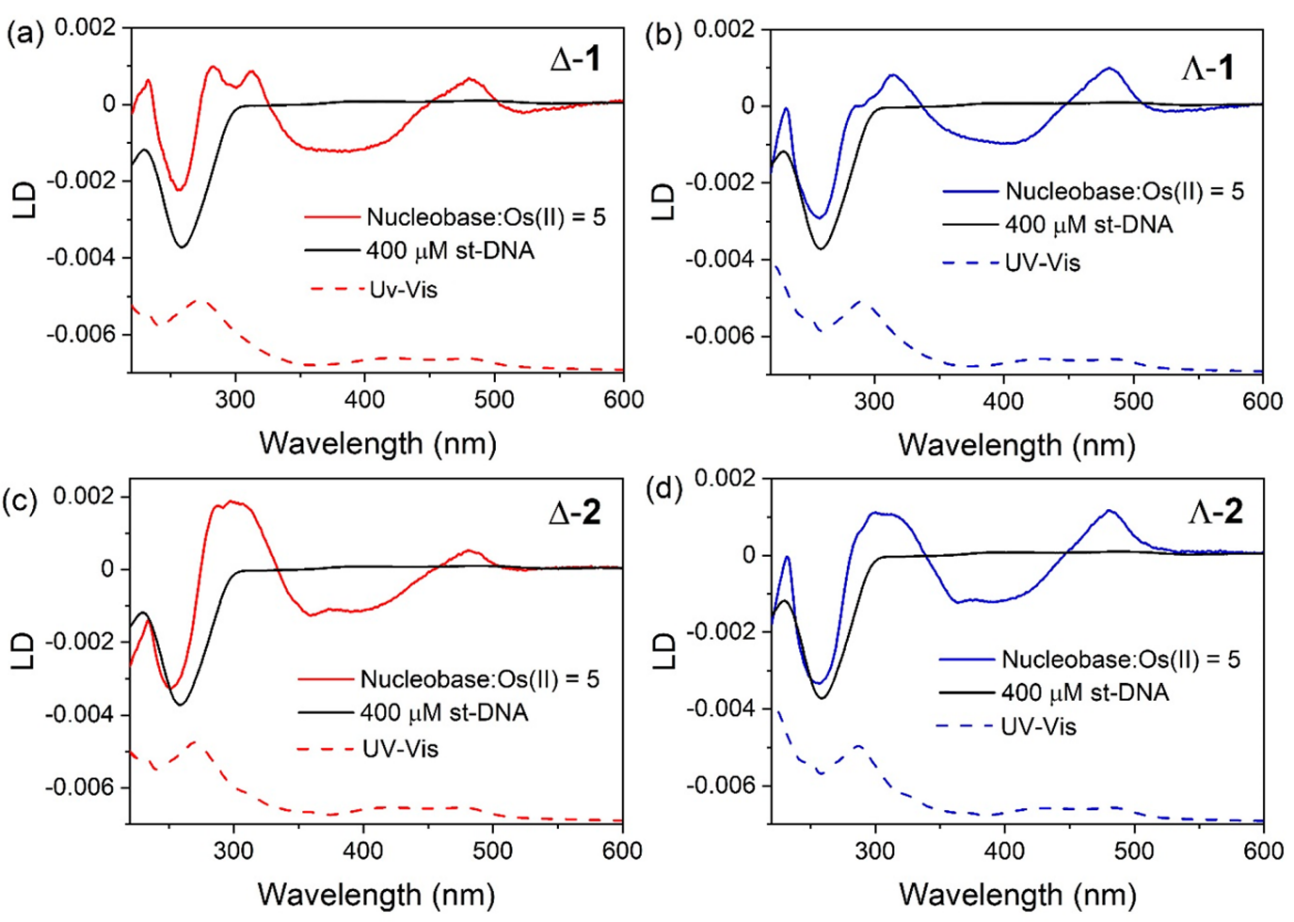
LD spectra of (a) Δ-**1**, (b) Λ-**1**, (c) Δ-**2**, and (d) Λ-**2** bound to st-DNA (400 μM) ([nucleobase]:[Os] = 5) in a 20 mM phosphate buffer at pH 7.0 (black line: 400 μM st-DNA).

CD and LD measurements were performed for the enantiomers of **1** and **2** in the presence of st-DNA. The CD measurements (Figure S30) revealed changes to the metal complex optical transitions in the presence of DNA, which reflected the shifts and hypochromism observed by visible absorption (Figures 4 and S20). Greater insight is provided by LD, which is a powerful technique for the study of DNA interactions because it exclusively reports on the bound complex without any contributions from the free complexes. In these measurements, a negative absorbance of the bound species indicates that the optical transition is aligned (parallel) with the DNA transitions, observed for intercalation, and a positive band suggests location in the groove. The interaction of Λ-**1** with flow-oriented st-DNA at a nucleotide-to-complex ratio of 5:1 yielded LD bands with opposite signs in the MLCT region of the spectrum at 400 nm (−) and 490 nm (+); additionally there is a positive band at 320 nm associated with ^1^LC transitions (Figure 7). As discussed earlier, TDDFT calculations for **1**^**2+**^ and **2**^**2+**^ indicate that the transitions between 450 and 500 nm are dominated by ^1^MLCT excitation involving charge transfer to the TAP ligands. On the other hand, transitions of between 350 and 450 nm involve charge-transfer excitation to the dppz and dppp2 ligands. The weaker negative band at wavelengths longer than 500 nm is calculated to correspond to excitations to ^3^MLCT states that have dppz or dppp2 localization. Changes in the LD signal of DNA at 260 nm arise due to a combination of ligand transitions in this region of the spectrum as well as a possible decrease in the orientation of the DNA sequence due to binding of the complex.^22^ Some differences are apparent in the LD spectrum obtained for Δ-**1**, which suggest a difference in the binding geometry. These trends were also observed for **2**. The data strongly support a DNA binding model involving intercalation of the dppz or dppp2 ligand, with the Os(TAP)_2_ unit residing in one of the grooves, as demonstrated previously for related ruthenium(II) intercalator complexes.^37,60^

### Thermal Denaturation

**Figure 8:**
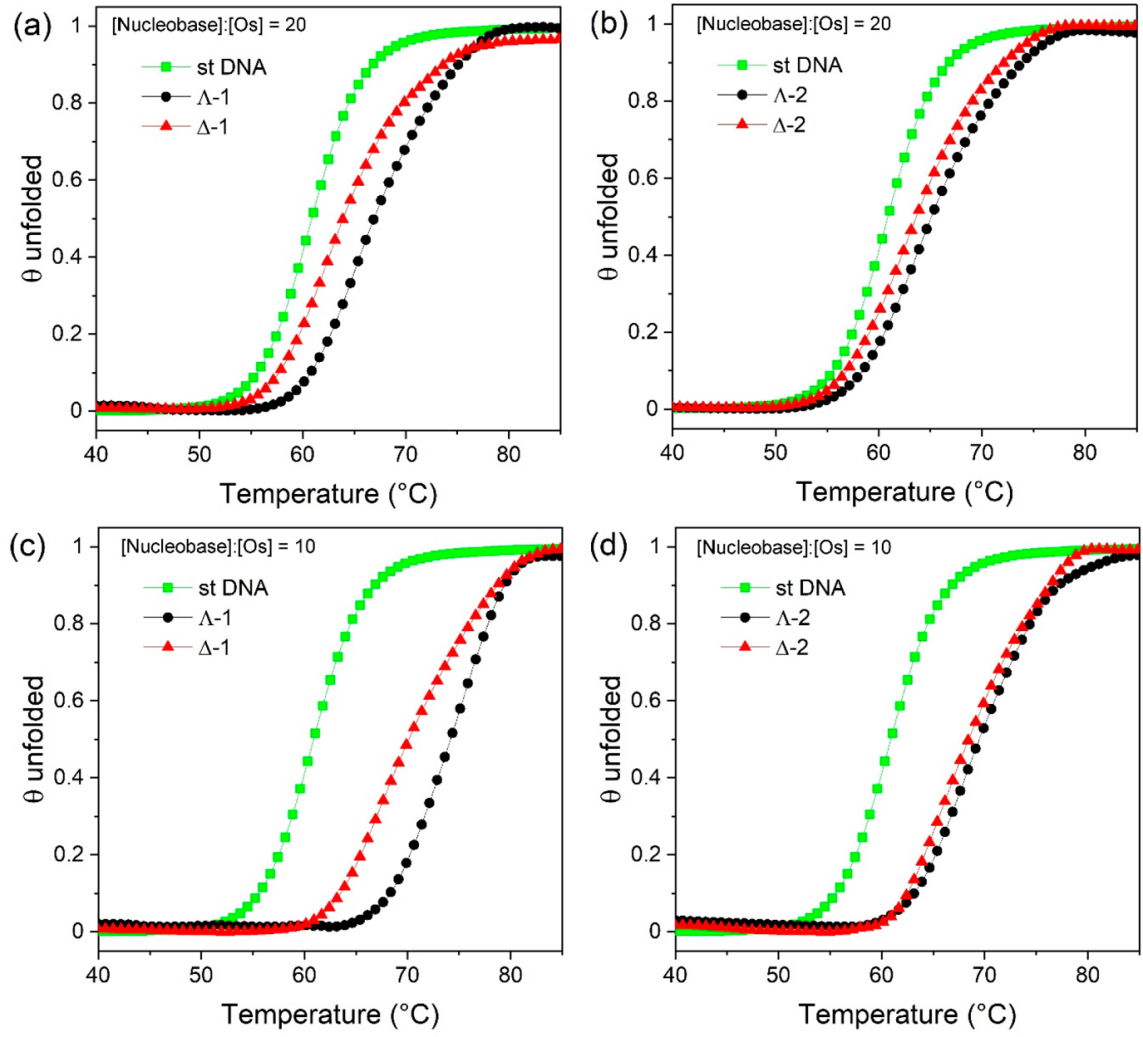
Fraction of DNA folded as a function of the temperature for Δ-**1** and Λ-**1** bound to st-DNA at (a) [nucleobase]:[Os] = 20 and (c) [nucleobase]:[Os] = 10 and for Δ-**2** and Λ-**2** bound to st-DNA at (b) [nucleobase]:[Os] = 20 and (d) [nucleobase]:[Os] = 10. For all, st-DNA (150 μM) in a 1 mM phosphate buffer and 2 mM NaCl at pH 7.0.

Next, the ability of the complexes to stabilize the DNA structure through binding interactions was investigated by determining the melting temperature (*T*_m_), which is the temperature needed to induce duplex dissociation. The change in the absorbance of 150 μM st-DNA at 260 nm was recorded in the absence and presence of the Δ and Λ enantiomers of **1** and **2** in a 1 mM phosphate buffer and 2 mM NaCl at pH 7.0. To examine the effect of different loadings of the complex on *T*_m_, these measurements were recorded at three different DNA nucleobase-to-osmium complex ratios of 50:1, 20:1, and 10:1. *T*_m_ for st-DNA alone was determined to be 60.9 ± 0.4 °C. Under conditions of the lowest loading (50:1), the complexes were found to cause a similar modest increase of 1–2 °C (Table S9 and Figure S31). However, at higher loadings, some differences emerged between the systems, which can be seen in the melting curves shown in Figure 8. Modest increases in *T*_m_ were observed at the 20:1 nucleobase/osmium(II) complex, with the greatest increase at 10:1 observed for Λ-**1**, which showed an increase of 13.4 ± 0.5 °C compared to the increase for the Δ enantiomer (Δ-**1**) of 9.3 ± 0.4 °C. Interestingly, complex **2** was found to be less stabilizing under 10:1 nucleobase/osmium(II) complex conditions, with an increase in *T*_m_ of 8.5 ± 0.4 °C for Δ-**2** and 7.9 ± 0.4 °C for Λ-**2**.

### Cellular Studies

**Figure 9:**
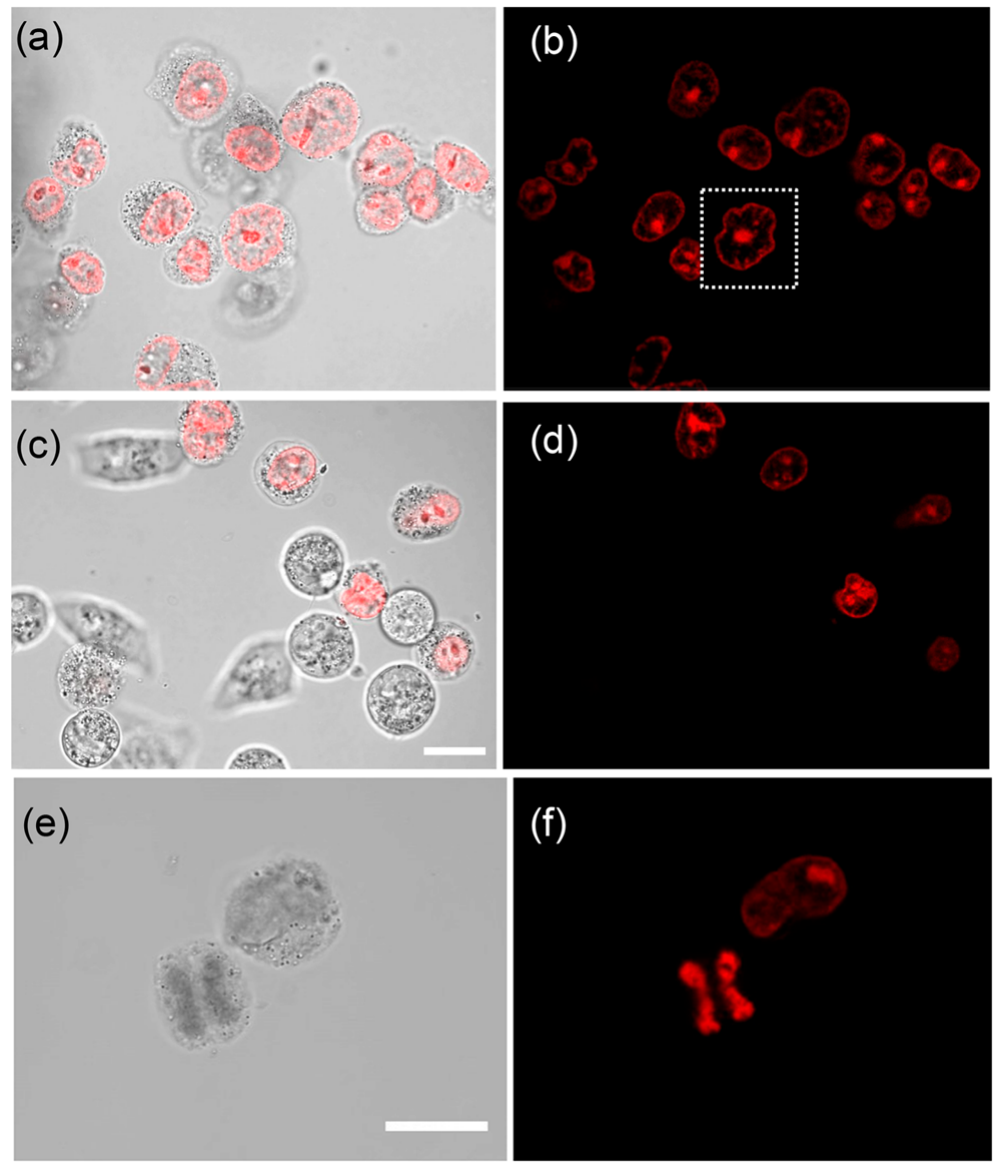
Live cell imaging. Transmission light and confocal phosphorescent images (λ_ex_ = 405 nm/λ_detection_ = 700–800 nm) for HeLa Kyoto cells incubated for 120 min with 50 μM of (a and b) **1** and (c and d) **2** and (e and f) 180 min for **1**. An example of a change in the nuclear envelope shape for cells incubated with **1** is highlighted in the white box. Scale bar = 20 μm.

Next, the cellular imaging potential of the complexes was investigated by examining the uptake of **1** and **2** by human cervical cancer cells (HeLa Kyoto cells). Incubation of the HeLa cells with complexes **1** and **2** alone did not result in internalization. Therefore, the complexes were encapsulated in polysorbate 80 (Tween 80), a nonionic surfactant that is recognized as safe and is commonly used in cell studies to aid uptake and allow cellular investigations.^61^ Formulation of the complexes was achieved using a film rehydration method that yields polymeric surfactant micelles containing the complexes.^61^ After 60 min of incubation with the cells, NIR emission indicated that both complexes were located exclusively in the nuclear envelope, and in addition there were typically two to three distinct subnuclear structures, resembling nucleoli (Figures S32 and S33). This is highlighted in Figure 9, which shows live cell images recorded for both complexes after 120 min of incubation. In order to confirm the nature of these subnuclear structures, HeLa cells were transfected with expression constructs encoding REXO4 fused to the enhanced yellow fluorescent protein (EYFP).^62^ REXO4, also known as HPMC2, is an exonuclease with DNA binding ability and has been previously localized to the nucleoli of cells.^63^ Imaging of HeLa cells that were expressing REXO4-EYFP and that had internalized either complex **1** or **2** showed a clear overlap between the two channels (Figure S34). Notably, there was no emission from the cytoplasm, which suggests that the complexes are not localized there.

Interestingly, in the case of **1**, a distinct change in the shape of the nuclear envelope was seen with a loss in the typical circular structure; this is highlighted in the white box in Figure 9. Uptake of the complex was not observed for all cells in the sample; this is attributed to the use of Tween 80 and is typical of systems where transfection agents are used.^61,64^ A particularly striking image was recorded for complex **1**, which showed the complex bound to two sets of daughter chromatids at the early anaphase step of mitosis (Figure 9e,f). Cell viability studies were performed using a method (Cell Titer-Glo) that measures the ATP content of cells, thereby acting as an indicator of their metabolic activity. A range of concentrations of the complexes, from 1 to 100 μM, was incubated with the cells for 3 h, followed by an assessment of their viability. The cells were seen to tolerate well the presence of both complexes at low concentrations; however, a decrease in the cell viability was seen to occur more sharply in the presence of complex **1** compared to complex **2**, with their respective IC50 values being determined at 45 and 90 μM, respectively (Figure S35).

Finally, we sought to explore the possible mechanisms by which the complexes become internalized into HeLa cells. The endocytic pathways used by cells are extremely diverse and in many cases are dictated by the cargo that needs to be internalized. As a first attempt to understand how the complexes enter, cells were treated with commonly used endocytic inhibitors targeting the three primary endocytic mechanisms. These were chlorpromazine (CPZ, an inhibitor of clathrin-mediated endocytosis), 5-(*N*-ethyl-*N*-isopropyl)amiloride (EIPA, an inhibitor of actin-mediated mechanisms, in particular macropinocytosis), and genistein (an inhibitor of caveolin-mediated endocytosis). Consistent with previous experiments, we noticed that, in general, complex **1** was internalized more readily than complex **2**. However, none of these inhibitors were able to abrogate the uptake of the complexes (Figure S36), as would have been expected if any single mechanism was used. Interestingly, the treatment of cells with EIPA resulted in a slight increase in the uptake of the complexes, perhaps suggesting the upregulation of other nonactin-mediated endocytic mechanisms in response to this drug. Overall, however, the results suggest that the conventional endocytic mechanisms used by the cells to internalize macromolecules are not the ones used in this case. Given that the complexes were only internalized when encapsulated with Tween 80, this is also suggestive that the mechanism is more likely to involve direct fusion with the plasma membrane, akin to the process of lipid-mediated transfection. Further, more detailed studies, using, for example, RNA interference tools targeting the regulation of plasma membrane lipids, will likely be needed to understand this.

## Discussion

We are interested in developing new osmium complexes capable of targeting DNA in complex environments. While there are significantly fewer studies of osmium polypyridyl complexes compared to their ruthenium counterparts, they show significant promise as cellular imaging agents.^7−11,13,14,32,40,65^ The current study reveals the interesting photophysical properties of Os-TAP complexes containing dppz (**1**) and dppp2 (**2**) intercalating ligands. DNA titrations show that the MLCT and LC transitions in the UV–visible absorption spectra are sensitive to the presence of DNA and undergo significant hypochromism. In particular, in the case of **2**, the LC band at 355 nm is a highly sensitive reporter of DNA binding (Figure 4b).

The architype light-switch complexes [M(phen)_2_(dppz)]^2+^, where M = Ru and Os, are emissive in MeCN but almost non-emissive in water.^20,27^ This arises through a mechanism involving a switch in the nature of the lowest-energy triplet excited state from an emissive “bright” ^3^MLCT state in MeCN to a non-emissive “dark” state associated with the dppz ligand in water due to hydrogen-bonding interactions with water molecules.^25^ The complexes detailed here also exhibit solvent-dependent NIR emission of ca. 750 nm, where the modest emission in water is significantly enhanced in MeCN. As a consequence, the emission observed in aqueous solution is attributed to ^3^MLCT states localized on the TAP ligands (however, computational results suggest that additional dppz/dppp2 ^3^MLCT and ^3^LC states will be relatively close in energy). This difference in character arises because of stabilization of the TAP-based orbitals relative to those of phen, which will favor population of ^3^MLCT states localized on TAP over those localized on dppz or dppp2. Furthermore, the emission of **1** is red-shifted from that observed for the related [Os(phen)_2_(dppz)]^2+^ light-switch complex (738 nm),^27^ while the low quantum yields observed for **1** and **2** are consistent with other NIR osmium(II)-based emitters (e.g., [Os(bpy)_3_]^2+^ and [Os(phen)_3_]^2+^).^15,66,67^

The enhanced luminescence observed for **1** when bound to DNA contrasts the behavior of the [Ru(TAP)_2_(dppz)]^2+^ counterpart, whose emission is quenched when binding to guanine-containing DNA.^38,57,68^ This is explained by the lower oxidation potential osmium(II/III) observed for **1** (+1.39 V vs Ag/AgCl) compared to that for the ruthenium(II) analogue (+1.82 V vs Ag/AgCl).^69^ The small increase in the emission of complex **1** in deaerated over aerated aqueous solutions suggests that protection from dissolved oxygen is a factor in the emission enhancement. The relative energies of the triplet excited states of differing character will also be strongly influenced by the solvent and DNA binding environment. Intercalation will shield the dppz or dppp2 ligand while leaving the TAP ligand exposed to water and hence subjected to hydrogen bonding to the noncoordinating N atoms. This will stabilize ^3^MLCT states associated with the TAP ligands and enhance the emission quantum yield and lifetime, while states associated with the intercalating ligand, which can undergo nonradiative deactivation, are comparatively destabilized and thus less accessible.

Interestingly, Λ-**1** is found to undergo greater emission enhancement in the presence of st-DNA than Δ-**1** (Table S8). This is in contrast to previous isostructural complexes, which have shown a greater increase in the quantum yield with the Δ enantiomer over the Λ enantiomer.^23^*In vivo* studies of [Os(phen)_2_(dppz)]^2+^ have also shown that Δ-[Os(phen)_2_(dppz)]^2+^ had enhanced emission in the nuclei of live cells compared to Λ-[Os(phen)_2_(dppz)]^2+^.^32^ In the case of both Λ enantiomers, a biphasic profile is observed in the change in the emission with increasing DNA concentration, with a similar biphasic behavior having been reported for a number of other complexes, and indicates that more than one binding site is possible and also indicates the sensitivity of the emission to the nature of the binding environement.^19,28,70−72^

The emission enhancement observed for the [Ru(phen)_2_(dppz)]^2+^ light-switch complex is known to be sensitive to the DNA composition and to emit more strongly when bound to AT polynucleotides than with GC polynucleotides.^24,73^ This is attributed to the degree of protection from the aqueous environment due to the difference in the binding site. For each complex, the greatest light-switch effect is observed when the Λ enantiomer binds to AT DNA, which contains the AT/AT step. This effect is greatest for **2**, where a 5.4-fold enhancement is observed. Interestingly, when bound to natural DNA (42% GC), the enhancement is reduced, which suggests that the complex does not bind at this AT/AT step. In the case of **2**, it should be noted that the enhancement observed in the presence of AT DNA is comparable to that observed in MeCN (Figure S13). DNA binding studies reveal that the enantiomers bind with high affinity to DNA (*K*_b_ of ca. 10^6^ M^–1^), in line with previous observations of related dppz complexes,^38^ and the general trend of a larger binding site size for the Λ enantiomer further indicates the different binding sites.

Previously, crystallography revealed a remarkable sensitivity of the structurally similar Λ-[Ru(phen)_2_(dppz)]^2+^ to the AT step sequence in a model oligonucleotide (5′-CCGGXXCCGG)_2_, where the central base step XX is either 5′-TA-3′ or 5′-AT-3′. It was observed that the reversal of a single base-pair step was found to impact the intercalation site, where the Λ-[Ru(phen)_2_(dppz)]^2+^ complex was found to intercalate at a central 5′-TA-3′ in the model sequence (5′-CCGGTACCGG)_2_ but bind to GC DNA in the (5′-CCGGATCCGG)_2_ sequence, where the central step was reversed.^58^ A follow-up study on the Λ-[Ru(TAP)_2_(dppz)]^2+^ complex confirmed that this preference was also observed in solution.^59^ This phenomenon was explored for the Λ-**2** complex, which had shown the greater sensitivity to AT versus GC binding (Figure 6d). The results of the titrations of Λ-**2** with the model sequences were found to demonstrate a similar sequence preference (Figures S27–S29), where a 3.8-fold emission enhancement was observed for Λ-**2** in the presence of (5′-CCGGTACCGG)_2_ compared to solution, suggesting binding to the central 5′-TA-3′ step. In contrast, a 2.3-fold emission enhancement of Λ-**2** was observed in the presence of (5′-CCGGATCCGG)_2_ compared to solution, which is characteristic of that observed for Λ-**2** in the presence of GC DNA (Figure 6d).

The LD spectra recorded for flow-oriented st-DNA in the presence of the complexes at a nucleobase-to-complex ratio of 5:1 indicate binding interactions in both the groove and through intercalation, which is to be expected for these complexes. Further insight regarding the different binding interactions was provided by thermal denaturation studies. Intercalation by dppz-type complexes typically results in an increase in the observed *T*_m_ due to stabilization of the double-stranded structure by stacking interactions of the planar ligands with the nucleobases.^74,75^ It is therefore not surprising that complexes **1** and **2** are found to increase the *T*_m_ even at low loadings with a nucleobase-to-complex ratio of 50:1 (Figure S31). What is interesting is the different behaviors observed for the two complexes, which are illustrated in the denaturation profiles shown in Figure 8. In the case of **1**, a clear difference is observed between the enantiomers at nucleobase-to-complex ratios of 20:1 and 10:1, where in both cases the Λ enantiomer shows the greatest stabilization. This mirrors observations for the emission of the complex in the presence of st-DNA. However, in contrast, the enantiomers of **2** are found to stabilize DNA to a similar extent. While the double-stranded DNA structure is stabilized by both stacking and hydrogen-bonding interactions, the stacking interactions provide the greater contribution.^76^ This suggests that the stacking interactions of **2** may be less optimal for stabilization. One reason that this may arise is if the complex participates in hydrogen bonding through the additional terminal N on the dppp2 ligand, which may induce a different stacking interaction. The role of hydrogen bonding to this N atom has previously been invoked to explain the photophysical properties of the related [Ru(bpy)_2_dppp2]^2+^ complex.^77^ Indeed, it would be interesting to see if a different binding is observed by X-ray crystallography.

A significant focus of this research is to develop cellular probes and explore the possibility for photoactive therapeutics. The results of the cellular studies are particularly interesting in that the simple modification of the intercalating ligand results in notably different cellular behavior of complexes **1** and **2** when incubated in the presence of the Tween 80 agent. The cells are highly tolerant of both complexes up to a concentration of 20 μM; however, at higher concentrations, a loss in cell viability is observed that is more stark with complex **1**. Both complexes are internalized by the cells, and monitoring of the NIR emission (700–800 nm) reveals that, after 60 min of incubation, they are exclusively localized in the nuclear membrane and nucleoli of the HeLa cells. A similar observation has been made for the uptake of the isostructural Os(phen)_2_dppz^2+^ complex by live cells.^32^ In particular, the complexes are found to stain the nuclear envelope and appear to label the nuclear lamina, which is composed of intermediate filament proteins, called lamins, that contribute to the structural integrity of the nucleus. Strikingly, in the case of **1**, the nuclear envelope was often seen to be severely disformed, a phenotype that is known to be a consequence of nuclear lamina breakdown (Figure 9, upper). This loss of structural integrity is the possible origin of the observed reduction in the cell viability.^78^ In this way, the clarity of the localization observed for complex **1** greatly aids in understanding the possible mechanism of cell death. Our preliminary studies using pharmacological inhibitors of different endocytic mechanisms were unable to identify a single mechanism for the uptake of these complexes into cells, and so further studies using a wider range of more specific perturbation tools, such as RNA interference reagents, will be needed in the future.

## Conclusion

Tuning the polypyridyl scaffold to modulate the optical properties of transition-metal complexes continues to prove an attractive strategy to develop new therapeutic and diagnostic agents. In this study, we report two intercalating osmium complexes comprising TAP ancillary ligands and show how subtle changes to the intercalating ligand, dppz versus dppp2, can result in differentiated enhanced emission to AT compared to GC DNA oligonucleotide sequences. Furthermore, we demonstrate the ability to distinguish binding to a 5′-TA-3′ versus a 5′-AT-3′ step in a model DNA system. Notably, we also show the impact on the cellular viability. Future work will continue to explore the origins of the different cellular behavior and to investigate how substituents on the intercalating ligand may tune the DNA affinity, cellular uptake, and localization.

## Experimental Section

The ancillary TAP was prepared using a known literature procedure.^41^ The dppz and dppp2 ligands were synthesized from 1,10-phenanthroline-5,6-dione using literature procedures.^42,79^ The purity of all ligands was confirmed by ^1^H NMR. All other chemicals employed were of reagent-grade quality from commercial sources and were used without further purification. The oligonucleotides were synthesized, desalted, and purified (by gel filtration) by Eurogentec (Liege, Belgium). Natural st-DNA was purchased from Sigma-Aldrich. Oligonucleotide and DNA concentrations were determined spectrophotometrically.

### Instrumental Methods

^1^H NMR spectra were obtained on a Varian VnmrS 400 MHz spectrometer. All electrospray ionization mass spectrometry studies were performed using an Agilent 6546 Q-TOF series LC/MS system. Cyclic voltammetry measurements in a dry, deaerated MeCN solution were carried out using a PalmSens EmStat3 potentiostat. The working electrode was a glassy carbon disk, and a platinum wire was employed as the counter electrode, while the reference electrode was Ag/AgCl. UV–visible absorption spectra were recorded on a Varian Cary 200 or a Varian Cary 50 spectrophotometer. Steady-state luminescence spectra were recorded on Varian Cary Eclipse and Horiba Fluoromax-4 spectrophotometers, and emission lifetime measurements were performed on an Edinburgh Instruments Mini-τ. CD measurements were recorded on a Jasco J-810 spectropolarimeter, and LD measurements were recorded on a Jasco J-810-150S CD spectropolarimeter.

### DNA Titrations

The concentration of DNA was determined using the molar absorbance at 260 nm for st-DNA (6600 M^–1^ cm^–1^/nucleotide), 5′-GCG-CGC-GCG-CGC-3′ (190300 M^–1^ cm^–1^/double strand), 5′-CCG-GAT-CCG-G-3′ (91800 M^–1^ cm^–1^/single strand), 5′-CCG-GTA-CCG-G-3′ (92200 M^–1^ cm^–1^/single strand), and 5′-ATA-TAT-ATA-TAT-3′ (190100 M^–1^ cm^–1^/double strand). UV–visible and emission titrations were carried out at [**1**] and [**2**] = 1.5 (±0.5) × 10^–5^ M at 298 K by monitoring changes in the absorption and emission spectra of the complexes upon successive additions of aliquots of DNA in a sodium phosphate buffer (20 mM, pH 7.0). The results are quoted using the concentration of DNA expressed as a concentration of the nucleobase [DNA]-to-Os ratio ([DNA]:Os ratio).

### Thermal Denaturation Studies

Thermal denaturation experiments were performed using a Cary 3500 UV–visible spectrophotometer. The temperature in the cell was ramped from 20 to 95 °C at a rate of 1 °C min^–1^, and the absorbance at 260, 355, 415, and 800 nm was measured every 0.2 °C data intervals. The data were fitted with a baseline correction in the single-stranded upper region (75–95 °C) using eq 1 to present the data in terms of fractional unfolded θ_T_.^80,81^
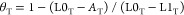
1

The data *T*_m_ was then determined from this corrected data by analyzing the 0.5 = θ_T_ value, which equates to *T*_m_.^80,81^ The melting data were repeated in at least duplicate, with the average of these runs plotted.

### pH Study

[**1**^2+^] (22.6 μM) and [**2**^2+^] (23.5 μM) were studied in various pH environments by the addition of 0.1 M HCl and 0.1 M NaOH in a 1 M NaCl buffer. The pH of the final solution was determined by a Mettler-Toledo pH microelectrode.

#### Synthesis of [Os(TAP)_2_(Cl)_2_]

[OsCl_6_][NH_4_]_2_ (250 mg, 0.57 mmol, 1 equiv), TAP (207 mg, 1.14 mmol, 2 equiv), and ethylene glycol (30 mL) were charged to a round-bottom flask under an N_2_ atmosphere. The solution was left at reflux for 1 h and then cooled to room temperature. A sodium hydrosulphite aqueous solution (50 mL, 1 M) was then added, with further cooling in an ice bath for 1 h. The resultant precipitate was collected by filtration and washed with cold H_2_O (100 mL), followed by diethyl ether (Et_2_O; 100 mL) to yield a dark-purple solid (347 mg, 0.55 mmol, 97%). ^1^H NMR (400 MHz, (CD_3_)_2_SO): δ 8.20 (d, *J* = 3.3 Hz, 2H), 8.21 (d, *J* = 3.3 Hz, 2H), 8.46 (d, *J* = 9.3 Hz, 2H), 8.56 (d, *J* = 9.3 Hz, 2H), 9.12 (d, *J* = 3.1 Hz, 2H), 9.95 (d, *J* = 3.1 Hz, 2H).

#### Synthesis of [Os(TAP)_2_(dppz)][PF_6_]_2_ [**1**^**2+**^·(PF_6_)_2_]

[Os(TAP)_2_(Cl)_2_] (250 mg, 0.400 mmol, 1 equiv), dppz (113 mg, 0.400 mmol, 1 equiv), and ethylene glycol (30 mL) were charged to a round-bottom flask under an N_2_ atmosphere. The reaction was left at reflux for 4.5 h, cooled to room temperature, and then treated with a NH_4_PF_6_ aqueous solution (300 mg in 20 mL, 1.84 mmol, excess) before being left to stir for 45 min. The resultant solids were collected by filtration and washed with cold water (30 mL) followed by Et_2_O (30 mL). Purification was achieved by column chromatography (SiO_2_, 10:1:1 MeCN/H_2_O/saturated aqueous KNO_3_) with collection of the third (brown) band. Subsequent counterion metathesis with NH_4_PF_6_ followed by recrystallization from MeCN/Et_2_O afforded the pure product as a dark-brown powder (215 mg, 0.191 mmol, 48%). ^1^H NMR (400 MHz, CD_3_CN): δ 7.82 (dd, *J* = 5.5 and 8.3 Hz, 2H), 8.11 (dd, *J* = 1.1 and 5.5 Hz, 2H), 8.14−8.19 (m, 2H), 8.22 (d, *J* = 3.0 Hz, 2H), 8.28 (d, *J* = 3.0 Hz, 2H), 8.47−8.53 (m, 2H), 8.62 (s, 4H), 8.80 (d, *J* = 3.0 Hz, 2H), 8.83 (d, *J* = 3.0 Hz, 2H), 9.60 (dd, *J* = 1.1 and 8.3 Hz, 2H). ^13^C NMR (101 MHz, CD_3_CN): δ 128.82, 130.66, 132.18, 133.73, 133.75, 133.91, 135.96, 140.63, 143.86, 145.91, 146.05, 146.93, 146.99, 148.33, 149.14, 151.44, 151.61, 152.48, 155.51. HRMS (ES). Calcd for [OsC_38_H_22_N_12_]^2+^: *m*/*z* 419.0847. Found: *m*/*z* 419.0845 (M^2+^).

#### Synthesis of [Os(TAP)_2_(dppp2)][Cl]_2_ [**2**^**2+**^·(Cl)_2_]

The dppp2 ligand was suspended with Os(TAP)_2_Cl_2_ (1 equiv) in an ethylene glycol mixture (4 mL) in a microwave vial. The mixture was irradiated under an inert atmosphere for 45 min at 473 K and then filtered to remove any unreacted [Os(TAP)_2_]Cl_2_. The reaction solution was then cooled to room temperature, before the addition of a saturated aqueous solution of NH_4_PF_6_ (2 mL). The suspension was collected by filtration and washed with deionized H_2_O and Et_2_O. Purification was achieved by column chromatography (Alumina, 40:4:1 MeCN/H_2_O/saturated aqueous NaNO_3_). The PF_6_ salt of the complex was reformed. This was converted to the water-soluble chloride salt by swirling of the PF_6_ complex in methanol (MeOH; 20 mL) in the presence of Amberlite ion-exchange resin (Cl form) for 1 h. The suspension was filtered and the solvent removed under reduced pressure. Further purification was then performed using a CM-Sephadex C25 column and a salt gradient between 0.01 and 0.1 M. The solvent was removed under reduced pressure and the excess NaCl removed by dissolution in chilled MeCN and filtering, the supernatant was isolated, and the solvent was removed under reduced pressure (successful counterion exchange was confirmed using ^19^F and ^31^P NMR spectra) to isolate the pure product [Os(TAP)_2_(dppp2)]·2Cl (45 mg, 16% yield). ^1^H NMR (400 MHz, D_2_O): δ 9.56 (ddd, *J* = 12.8, 8.3, and 1.3 Hz, 2H), 9.34 (dd, *J* = 4.1 and 1.9 Hz, 1H), 8.87 (dd, *J* = 8.6 and 1.9 Hz, 1H), 8.72 (d, *J* = 3.1 Hz, 2H), 8.70 (d, *J* = 3.0 Hz, 2H), 8.54 (s, 4H), 8.33 (dd, *J* = 3.1 and 1.6 Hz, 2H), 8.21 (d, *J* = 3.1 Hz, 2H), 8.09–7.98 (m, 3H), 7.77 (ddd, *J* = 8.3, 5.5, and 4.4 Hz, 2H). ^13^C NMR (126 MHz, CD_3_OD): δ 157.45, 154.25, 154.09, 152.36, 152.10, 150.68, 150.47, 149.38, 147.53, 147.51, 146.96, 146.07, 145.99, 144.99, 144.80, 142.23, 141.08, 139.23, 138.94, 135.70, 135.43, 132.81, 131.07, 130.85, 128.06, 128.01, 127.58. HRMS (ES). Calcd for [OsC_37_H_21_N_13_Cl]^+^: *m*/*z* 874.1331. Found: *m*/*z* 874.1339 ([OsC_37_H_21_N_13_Cl]^+^).

#### Counterion Metathesis

[Os(TAP)_2_(dppz)][1^2+^][PF_6_]_2_ (100 mg, 0.089 mmol) and 5 equiv wt of Amberlite Cl form ion-exchange resin were combined in MeOH (50 mL) and stirred at room temperature in the dark overnight. The resin was removed by filtration and the filtrate evaporated to dryness. The residue was subjected to flash column chromatography (Al_2_O_3_, 7% MeOH/CH_2_Cl_2_), with collection of the brown band and subsequent recrystallization from MeCN/Et_2_O, affording the pure chloride salt of **1^2+^** as a dark-brown powder. Yield: 68 mg, 84%. ^1^H NMR (400 MHz, CD_3_CN, 298 K): δ 7.82 (dd, *J* = 5.6 and 8.1 Hz, 2H), 8.12 (dd, *J* = 1.2 and 5.5 Hz, 2H), 8.14–8.19 (m, 2H), 8.23 (d, *J* = 3.0 Hz, 2H), 8.28 (dd, *J* = 3.0 Hz, 2H), 8.47–8.52 (m, 2H), 8.62 (s, 4H), 8.80 (d, *J* = 3.0 Hz, 2H), 8.83 (d, *J* = 3.0 Hz, 2H), 9.60 (dd, *J* = 1.2 and 8.3 Hz, 2H). ^13^C NMR (101 MHz, CD_3_OD, 298 K): δ 129.24, 130.90, 132.85, 133.81, 134.19, 136.61, 141.11, 144.32, 146.24, 146.45, 147.38, 147.45, 148.33, 148.95, 151.90, 152.09, 153.09, 154.99. HRMS (ES). Calcd for [OsC_38_H_22_N_12_]^2+^: *m*/*z* 419.0847. Found: *m*/*z* 419.0847 (M^2+^). Calcd for [OsC_38_H_22_N_12_Cl]^+^: *m*/*z* 873.1388. Found: *m*/*z* 873.1382 [([M][Cl])^+^]. Successful counterion metathesis was confirmed through the complete absence of resonances in both ^19^F and ^31^P NMR spectra.

### Formulation in Polysorbate 80

Compounds **1** and **2** (2 mg) were dissolved in an organic phase containing 2 mL of acetone and 0.05 mL of dichloromethane, and Polysorbate 80 (Tween 80) was added. The organic solvent was removed under reduced pressure at 40 °C to form a brown film and resuspended in a 5 mL aqueous phase.^61^ The volume was further reduced to 1 mL and stored at 4 °C prior to being added to the imaging media.

### Cell Culture

HeLa Kyoto (CVCL_1922) cells were cultured in Dulbecco’s modified Eagle medium, with 10% fetal bovine serum (FBS) and 1% l-glutamine (complete medium) (ThermoFisher) at 37 °C with 5% CO_2_. Cells were plated into 35 mm glass-bottomed dishes (MatTek Corp.; 120000 cells in 2 mL of the medium). The next day cells were treated with formulated complexes as follows. Cells were first washed briefly with a FluoroBrite medium (ThermoFisher) supplemented with 1% FBS (together termed the imaging medium). The Tween 80-formulated complexes **1** and **2** were diluted in the imaging medium to a final concentration of 50 μM and added to the cells. The cells were incubated in the dark at 37 °C with 5% CO_2_ until imaging.

### Cell Viability Assay

A total of 7500 HeLa Kyoto cells were plated into 96-well white-walled luminescence plates (PerkinElmer) in the complete medium. Prior to performance of the cell viability assay, the Cell Titer-Glo reagent (Promega) was allowed to thaw at room temperature. A total of 24 h after plating, the cells were washed briefly with the imaging medium. The Tween 80-formulated complexes **1** and **2** were diluted in the imaging medium to a range of concentrations of 1–100 μM, added to the cells, and incubated at 37 °C for 2.5 h. Following this incubation, Cell Titer-Glo was added in a 1:1 ratio to the cell culture medium, and incubation was continued for 30 min at room temperature. The plates were agitated for 2 min, followed by a further 10 min incubation at room temperature. Luminescence was recorded on a SPARK 10 M plate reader (Tecan), and the results were analyzed. Background luminescence readings were taken from the wells without cells but containing the cell culture medium and Cell Titer-Glo reagent, and these were subtracted from the values for all of the other wells. Luminescence was determined from three replicate wells for each condition, and the experiment was repeated twice.

### Cell Imaging

Images were acquired using an Olympus FV3000 confocal microscope fitted with a live cell environmental chamber. All images were acquired using a 60×/1.4 NA oil immersion objective. At 1 h prior to imaging, the climate control system was turned on to obtain an optimal temperature of 37 °C for live cell imaging. The Tween 80-formulated complexes **1** and **2** were excited using a 405 nm laser line and detected with the spectral detection window set to 700–800 nm at various times after addition of the complexes. Transmitted light images were acquired using the backscatter from the 405 nm laser illumination. For the colocalization experiments, the cells were transfected the day prior to addition of the complexes. For transfection, 1 μg of an expression plasmid encoding EYFP-REXO4 was transfected using 3 μL of Lipofectamine 3000 (ThermoFisher) in the OptiMEM medium according to the manufacturer’s instructions. The EYFP was excited was using a 514 nm laser line and detected between 520 and 570 nm. For the endocytic inhibitor study, the cells were plated into 35 mm glass-bottomed dishes (MatTek Corp.), as described above. The following day cells were treated with various endocytic inhibitors, CPZ (1 μg/mL), EIPA (300 μM), or genistein (300 μM). After 1 h, the complete medium was removed, and the cells were briefly washed with the imaging medium. Both complexes were then diluted to a concentration of 50 μM in the imaging medium and incubated for 2 h at 37 °C prior to imaging. Image acquisition from the live cells was performed using an Olympus FV3000 laser scanning confocal microscope as described above. Following image acquisition, the number of cells containing internalized complex were counted and expressed as a percentage of the total number of cells in the field of view. A minimum of 150 cells were counted for each condition.

## Supporting Information Available

Additional experimental details, materials, and methods, including spectroscopic data from titrations and cellular studies (PDF)
Data for the BVS analysis (XLSX)
