# Supplementary material for: Photophysical Properties and DNA Binding of Two Intercalating Osmium Polypyridyl Complexes Showing Light-Switch Effects
Source: Inorg Chem. 2022 Sep 12;61(38):14947–61. doi: 10.1021/acs.inorgchem.2c01231 (PMC9516684; doi:10.1021/acs.inorgchem.2c01231)
Supplement: Supplementary file 1 — ic2c01231_si_001.pdf [file ic2c01231_si_001.pdf]

Supporting Information

Photophysical Properties and DNA Binding of two Intercalating Osmium Polypyridyl Complexes Showing Light Switch Effects

Mark Stitch,[a] Rayhaan Z. Boota,[b] Alannah S. Chalkley,[c] Tony D. Keene,[a] Jeremy C. Simpson,[c*] Paul A. Scattergood,[b*] Paul I. P. Elliott[b*] and Susan J Quinn[a*]

[a]*School of Chemistry, University College Dublin, Dublin 4, Ireland.*

[b]*Department of Chemical Sciences, School of Applied Sciences University of Huddersfield, Queensgate, Huddersfield, UK*

[c]*Cell Screening Laboratory, School of Biology & Environmental Science, University College Dublin, Dublin 4, Ireland.*

Experimental

X-ray Crystallography

The single crystal structure of compound **A** was collected on an Oxford Diffraction Supernova diffractometer using Cu-Kα radiation (λ = 1.54184 Å) at 120 K. The sample was mounted using a home-built dry ice cold mounting stage to prevent solvent loss from the crystals. Data was collected and integrated using CrysAlisPro[1] with a numerical absorption correction made using the same software. The structure was solved using SHELXT[2] and refined using SHELXL-2016[2] in Olex2.[3] Hydrogen atoms were generated in geometric positions and refined in riding mode on the parent atom.

Chiral Resolution of [Os(TAP)$_2$(dppz)] and [Os(TAP)$_2$(dppp2]

The column was loaded with a slurry of cation exchange CM Sephadex C-25 (GE healthcare), previously soaked overnight in water and then thoroughly degassed for 1 h, and allowed to settle overnight before passing water through the column for 2 h. A solution of (10 mg) the racemic [**1**$^{2+}$][Cl]$_2$ and [**2**$^{2+}$][Cl]$_2$ was dissolved in a 10 mL water prior to loading onto the Sephadex. Nanopure water was passed through the column until the complex was adsorbed fully onto the Sephadex and the water above was colourless. Additional CM Sephadex C-25 slurry was then added to top of the column to protect the loading bed from being disturbed and water was further passed through the column for 1 h to ensure all the complex was electrostatically bound. The mobile phase was then changed to aqueous (−)-O,O′-dibenzoyl-L-tartrate (Sigma-Aldrich) (0.1 M) and the pump speed was gradually increased to 1 mL min^{-1}. The column was kept in darkness when being used. Additional Sephadex slurry was added during the running of the column to maintain the height of the Sephadex, as the increased ionic strength of the mobile phase caused the Sephadex to shrink during the resolution. During recycling, the speed of the pump was reduced to 0.1 mL min^{-1} Clear separate bands were observed with the lambda enantiomer eluting first. The enantiopure species were isolated by gentle shaking with amberlite beads for a period of 8 h, the removal of tartrate was monitored via UV vis spectroscopy. The solvent was then removed under a reduced pressure to remove the excess NaCl the complex was dissolved in MeCN and left in the fridge overnight. The enantiomers were further collected as their PF_6^- salt using a concentrated solution of NH_4PF_6. The precipitate was collected via centrifugation and the precipitate was further washed with water 3 times. The chloride form was regenerated by shaking in methanol with amberlite IRA 402 (Sigma-Aldrich) beads at 5°C. The enantiomeric purity of the fractions was then analysed using circular dichroism and the Δε were recorded to ensure enantiomeric purity.

X-ray Crystallography Results

Complex **1** crystallises in the monoclinic space group *I*2/*a* (no. 15) and the asymmetric unit consists of one osmium(II) cation, one 1,4,5,8-tetraazaphenanthrene (TAP) ligand, half of a dipyrido[3,2-a:2',3'-c]phenazine (Dppz) ligand, one hexafluorophosphate anion and a diethyl ether molecule (Figure 1 and Figure S2a). The osmium cation is approximately octahedrally coordinated through chelation by two TAP and one DPPZ ligand (average Os−N = 2.060 Å). The Os1 cation and Dppz ligands lie along a 2-fold rotation axis, which also replicates the symmetry equivalent TAP ligand to complete the coordination sphere of Os1. This same rotation also generates the other PF_6^- anion and second diethyl ether molecule to complete the formula unit. The DPPZ ligand displays a slight torsion along the length of the molecule with a 7.4(3)° dihedral between the phenanthroline and terminal phenyl mean planes. Complex **1** take part in van der Waal's interactions with the diethylether molecules to form chain-like structures in the *a*-axis (Figure S2b). The diethyl ether and hexafluorophosphate groups have large ellipsoids, but an improved model was not obtained through modelling with disorder on these sites.

Bond valence sum analysis

To date, there are no bond valence sum parameters recorded for Os(II) or Os(III). To confirm the oxidation state of Os in compound A, we undertook a survey of the Cambridge Crystallographic Database (2021 version, data given in ESI) of Os(II)N_6 and Os(III)N_6 coordination compounds. From the list of extracted bond lengths, we were able to determine r_0 parameters for OsN_6 in both oxidation states.

$$V = \sum \exp\left(\frac{r_0 - r}{B}\right)$$

B was fixed to 0.37 and r_0 adjusted to give the closest fit to the expected oxidation state, giving Os(II)N_6 r_0 = 1.646 Å and Os(III)N_6 r_0 = 1.850 Å. Os has an unusual lengthening of bonds on oxidation from +2 to +3 (average Os(II)−N = 2.054 Å and Os(III)−N = 2.107 Å), so using the wrong r_0 does not give the correct oxidation state, as would normally occur. Analysis of the Os−N bond lengths of compound **A** give a BVS of 2+ (1.97) using the r_o = 1.646 Å for Os(II).

Table S1: Bond valence sum results using r_0 values obtained from the CSD.

	Os(II) r_o = 1.646 Å	Os(III) r_o = 1.850 Å
Os(II)−N	2.00(11)	3.48(19)
Os(III)−N	1.73(4)	3.00(7)

Crystallographic parameters for 1

Empirical formula	$C_{46}H_{42}F_{12}N_{12}O_2OsP_2$
Formula weight	1275.05
Temperature / K	120(1)
Crystal system	monoclinic
Space group	*I*2/*a* (no. 15)
a / Å	16.0831(3)
b / Å	17.8317(6)
c / Å	16.5931(5)
α / °	90
β / °	97.854(2)
γ / °	90
Volume / $Å^3$	4714.1(2)
Z	4
ρ_{calc} g/cm^3	1.797
μ / mm^{-1}	6.668
F(000)	2528.0
Crystal size / mm^3	0.17 × 0.04 × 0.015
Radiation	Cu-$K\alpha$ (λ = 1.54184)
2θ range for data collection / °	7.314 to 133.162
Index ranges	$-19 \le h \le 13$, $-21 \le k \le 21$, $-19 \le l \le 19$
Reflections collected	12736
Independent reflections	4152 [R_{int} = 0.0389, R_{sigma} = 0.0380]
Data/restraints/parameters	4152/0/341
Goodness-of-fit on F^2	1.060
Final *R* indexes [$I > 2\sigma(I)$]	R_1 = 0.0592, wR_2 = 0.1472
Final *R* indexes [all data]	R_1 = 0.0763, wR_2 = 0.1633
Largest diff. peak/hole / e $Å^{-3}$	2.51/−1.79

DNA Binding Constant Calculations

The intrinsic binding method described by Bard *et al.*[4] in the equation shown below (**1**) was used to fit both the absorption and emission data. The binding constant of the [**1**]$^{2+}$ and [**2**]$^{2+}$ enantiomers binding to DNA structures was determined by fitting equation (**1)** to a non-linear plot of ($\varepsilon_a - \varepsilon_f$)/($\varepsilon_b - \varepsilon_f$) vs [DNA] for the molar absorption and (**2**) ($I_a - I_f$)/($I_b - I_f$) vs [DNA], for the molar emission (with [DNA] concentration in nucleotides, fitted using Origin 2021 software).

$$\frac{(\varepsilon_a - \varepsilon_f)}{(\varepsilon_b - \varepsilon_f)} = (b - (b^2 - 2{K_b}^2 C_t[DNA]/s)^{\frac{1}{2}})/2K_b C_t \qquad (1)$$

$$\frac{(I_a - I_f)}{(I_b - I_f)} = (b - (b^2 - 2{K_b}^2 C_t[DNA]/s)^{\frac{1}{2}})/2K_b C_t \qquad (2)$$

$$b = 1 + K_b C_t + K_b[DNA]/2s \qquad (3)$$

Where; K is the equilibrium binding constant in M^{-1}, C_t the total metal complex concentration (changes to this are only due to dilution by the addition of DNA), *s* the binding site size and [DNA] the concentration of DNA in nucleotides. ε_f is the extinction coefficient of free metal complex ($\varepsilon_f = A_{initial}/C_t$), ε_a the apparent extinction coefficient ($\varepsilon_a = A_{observed}/C_t$) and ε_b the extinction coefficient of the metal complex in the fully bound form ($\varepsilon_b = A_{final}/C_t$), A = Absorbance. I_f is the molar emission of free metal complex (I_f = initial E_m /Ct), I_a the apparent molar emission (I_a = apparent E_m/C_t) and I_b the molar emission of the metal complex in the fully bound form (I_b = Final E_m/C_t), E_m = emission.

Figures and Schemes

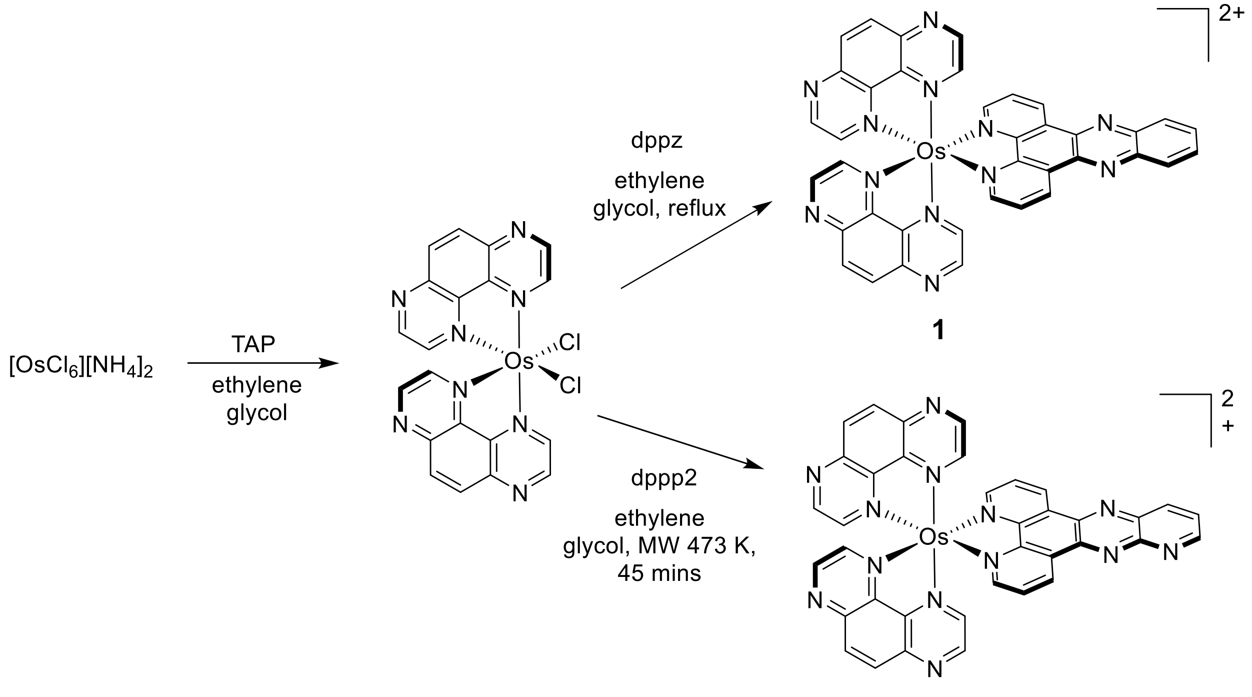

1

2

Scheme 1 Synthesis of **1**$^{2+}$ and **2**$^{2+}$ (prepared as both the chloride and PF_6^- salts).

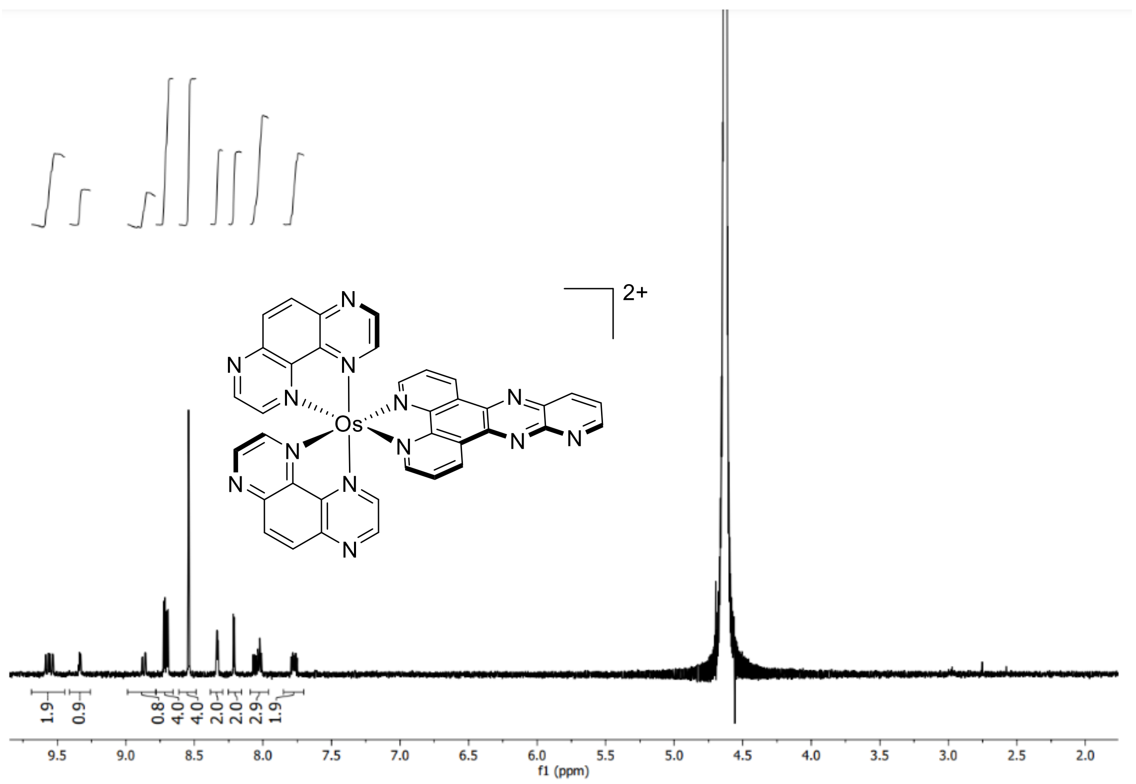

Figure S1: ^{1}H NMR spectra of [Os(TAP)$_2$(dppp2)].2Cl (400 MHz, D_2O).

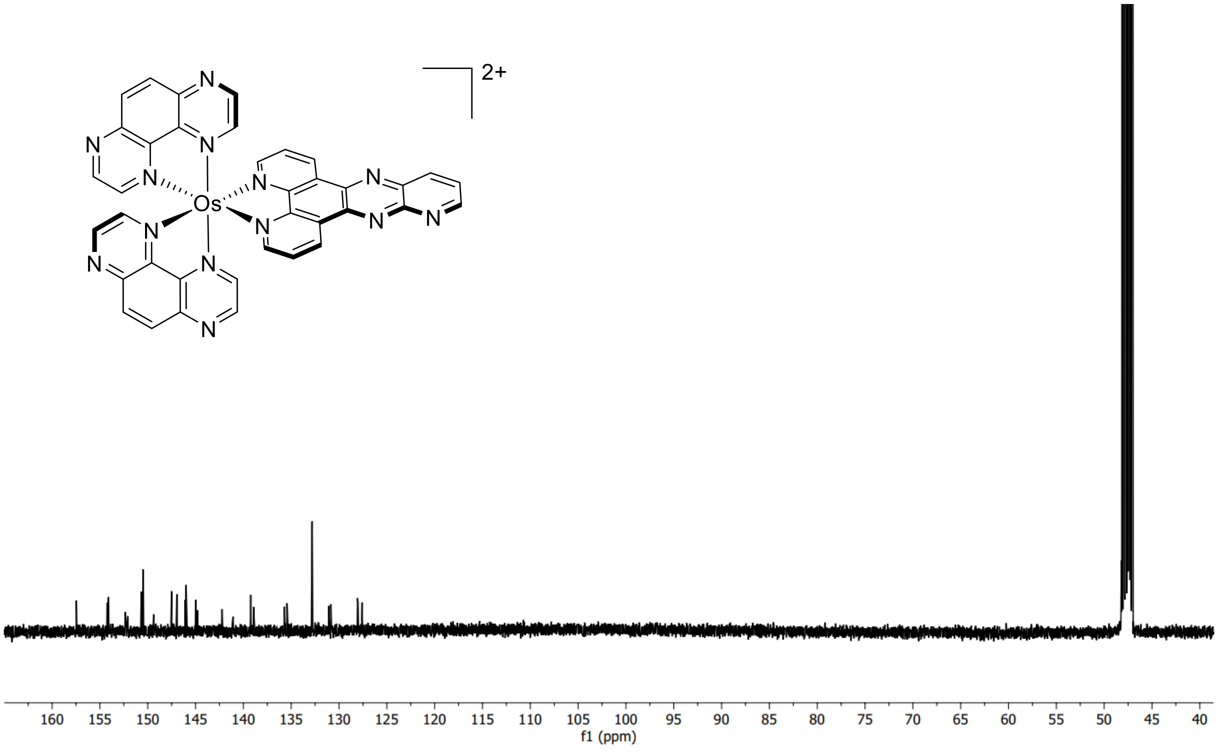

Figure S2: ^{13}C NMR spectra of [Os(TAP)$_2$(dppp2)].2Cl (126 MHz, CD_3OD)

Spectrum Peaks

m/z	Z	Abund	Diff (ppm)	Height %	Height % (Calc)	Ion Species	Formula
870.1284	1	2608	-0.06	30.74	26.99	M+[-Cl]	C37H21Cl2N13Os
871.1307	1	3610	0.01	42.55	42.31	M+[-Cl]	C37H21Cl2N13Os
872.1315	1	5556	0.68	65.49	71.99	M+[-Cl]	C37H21Cl2N13Os
873.1317	1	2815	-0.25	33.18	37.88	M+[-Cl]	C37H21Cl2N13Os
874.1339	1	8484	0.95	100.00	100.00	M+[-Cl]	C37H21Cl2N13Os
875.1360	1	3751	0.25	44.22	42.18	M+[-Cl]	C37H21Cl2N13Os
876.1327	1	2651	-0.56	31.25	32.93	M+[-Cl]	C37H21Cl2N13Os
878.1345	1	294	-2.77	3.47	2.49	M+[-Cl]	C37H21Cl2N13Os

Compound Spectra

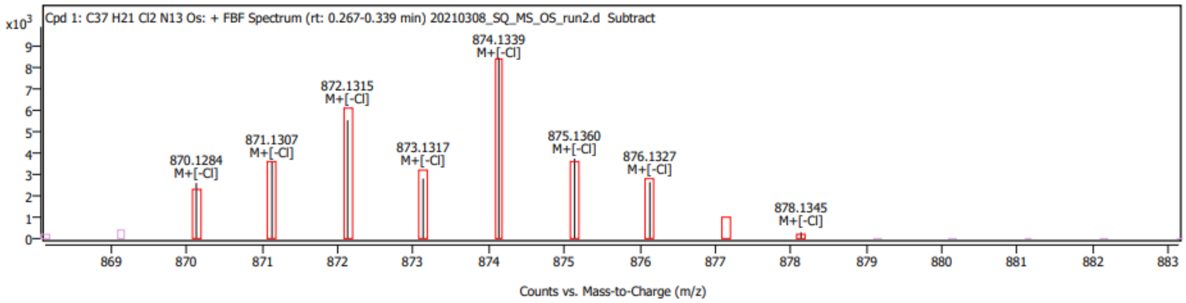

Figure S3: HRMS data for $[Os(TAP)_2(dppp2)].2Cl$ ($([M][Cl])^+$)

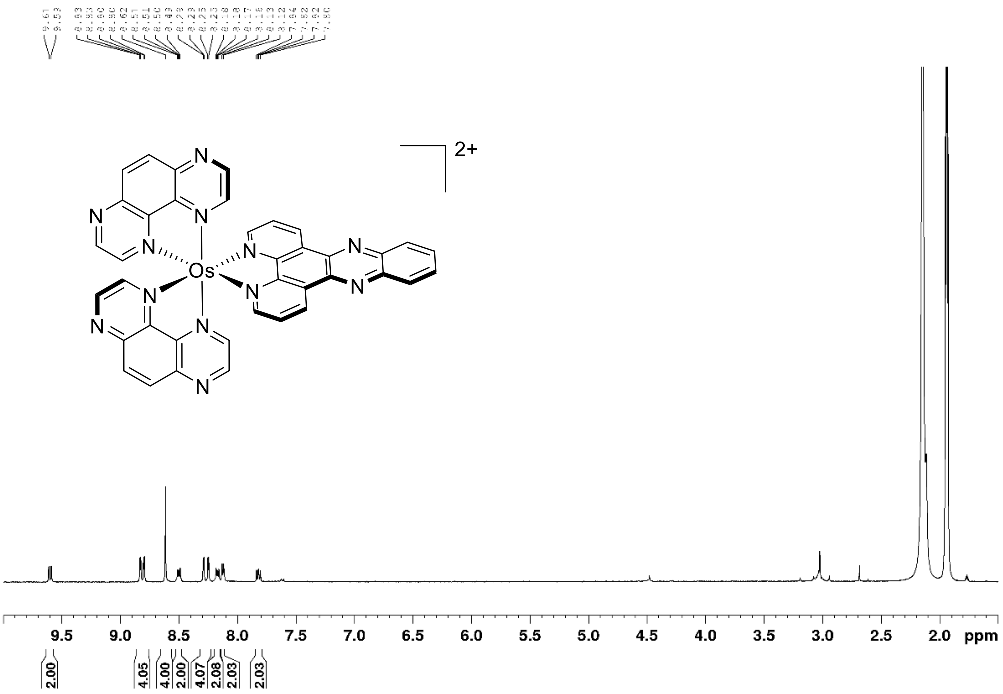

Figure S4: ^{1}H NMR spectra of [Os(TAP)$_2$(dppz)].2Cl (400 MHz, CD_3CN).

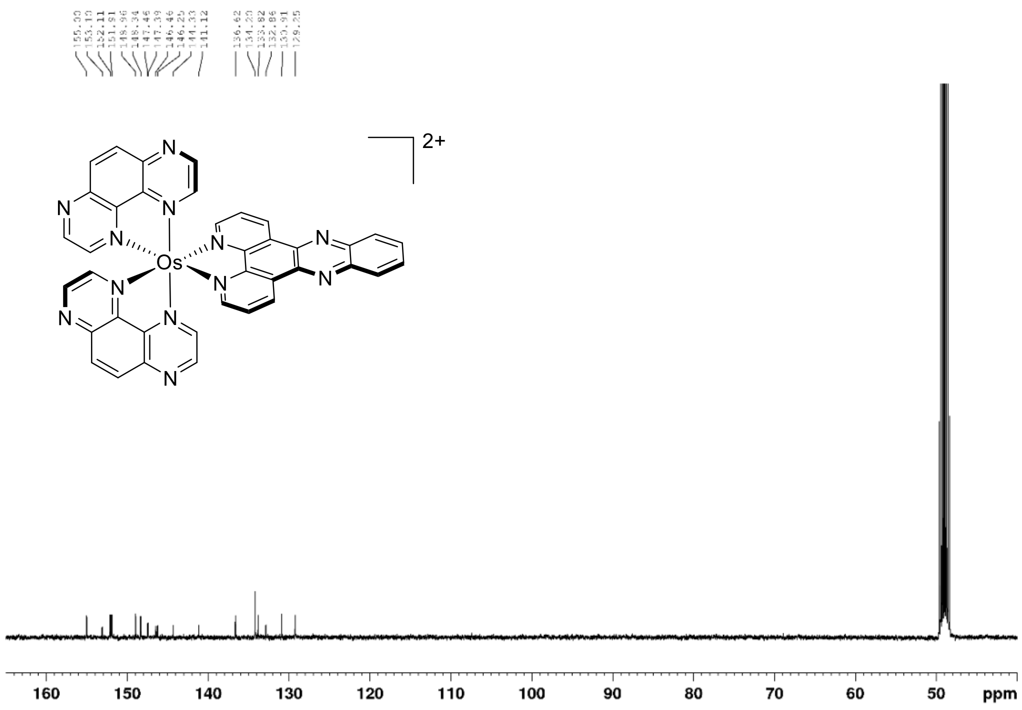

Figure S5: ^{13}C NMR spectra of [Os(TAP)$_2$(dppz)].2Cl (101 MHz, CD_3OD)

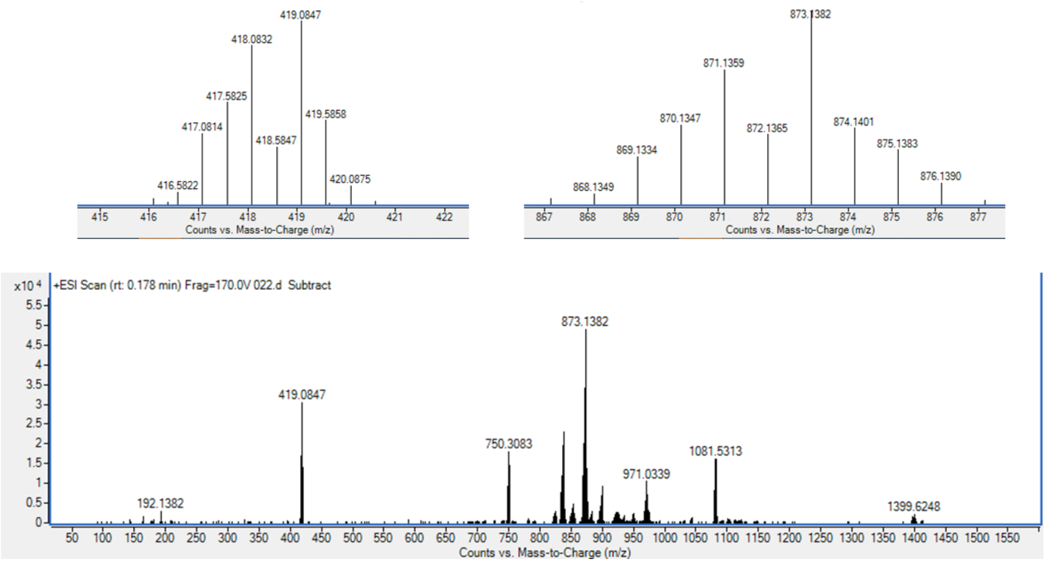

Figure S6: HRMS data for [Os(TAP)$_2$(dppz)].2Cl ([M][Cl])$^+$

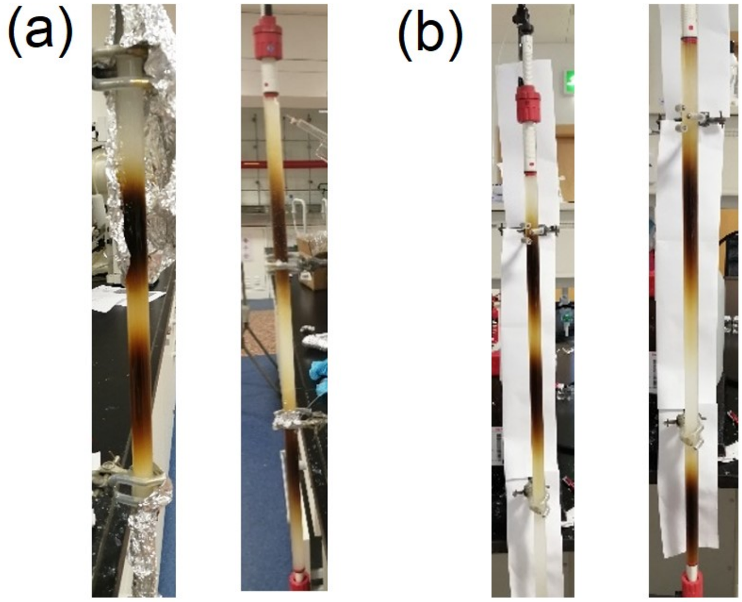

Figure S7: The two bands after (a) 2 passes for complex **1** (b) three passes for complex **2**.

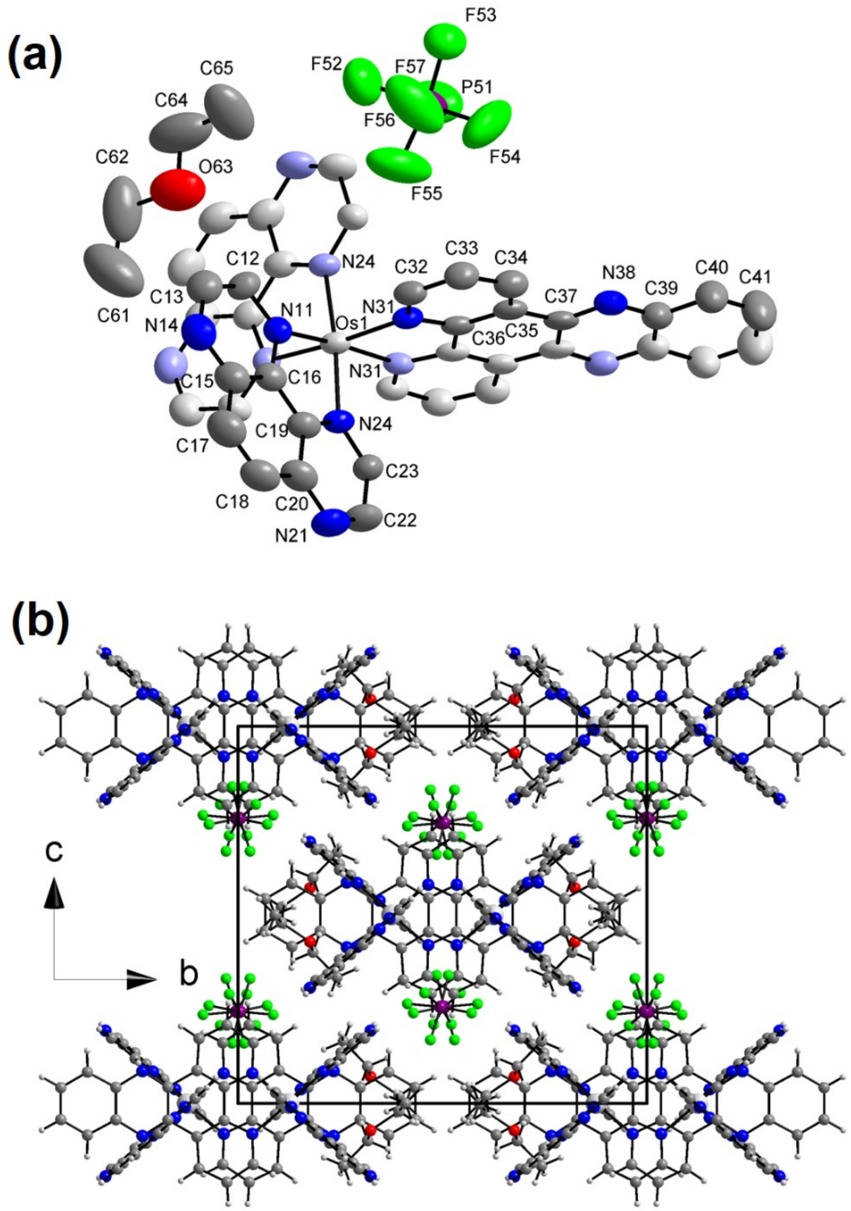

Figure S8: (a) Asymmetric unit and selected symmetry equivalents of complex **1**. Symmetry equivalents are greyed out (i: 1.5−x, y, 1−z) and hydrogen atoms removed for clarity. Thermal ellipsoids are at the 50% probability level. (b) Packing diagram of **1** looking down the a-axis, illustrating the chain-like interactions of the compound in that axis.

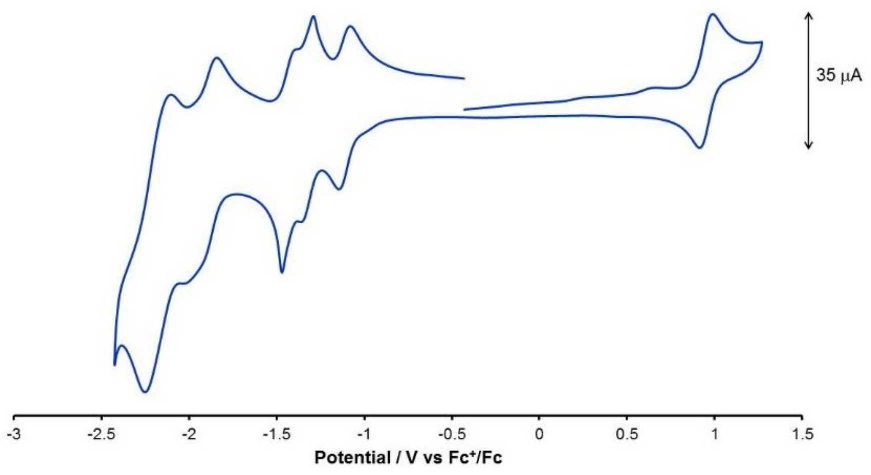

Figure S9: Cyclic voltammogram recorded at r.t. at 100 mVs^{-1} for a 1.5 $mmol^{-1}dm^{3}$ MeCN solution of [$\mathbf{1}^{2+}$][PF_6]$_2$. NBu_4PF_6 was employed as a supporting electrolyte, with a solution concentration of 0.2 $mol^{-1}dm^{3}$. All potentials are shown against the Fc^{+}/Fc couple.

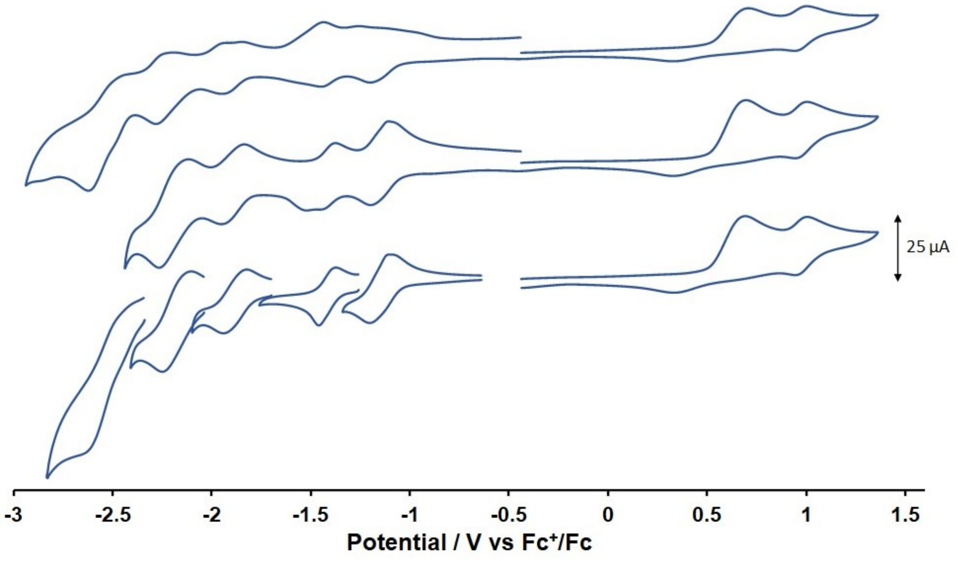

Figure S10: Cyclic voltammograms recorded at 100 mVs^{-1} for a 1.5 $mmoldm^{-3}$ MeCN solution of [$\mathbf{2}^{2+}$][PF_6]$_2$. Scans were also performed for each reduction wave in turn to assist in resolving electrochemical couples (bottom). NBu_4PF_6 was employed as a supporting electrolyte, with a solution concentration of 0.2 $mol^{-1}dm^{3}$. All potentials are shown against the Fc^{+}/Fc couple.

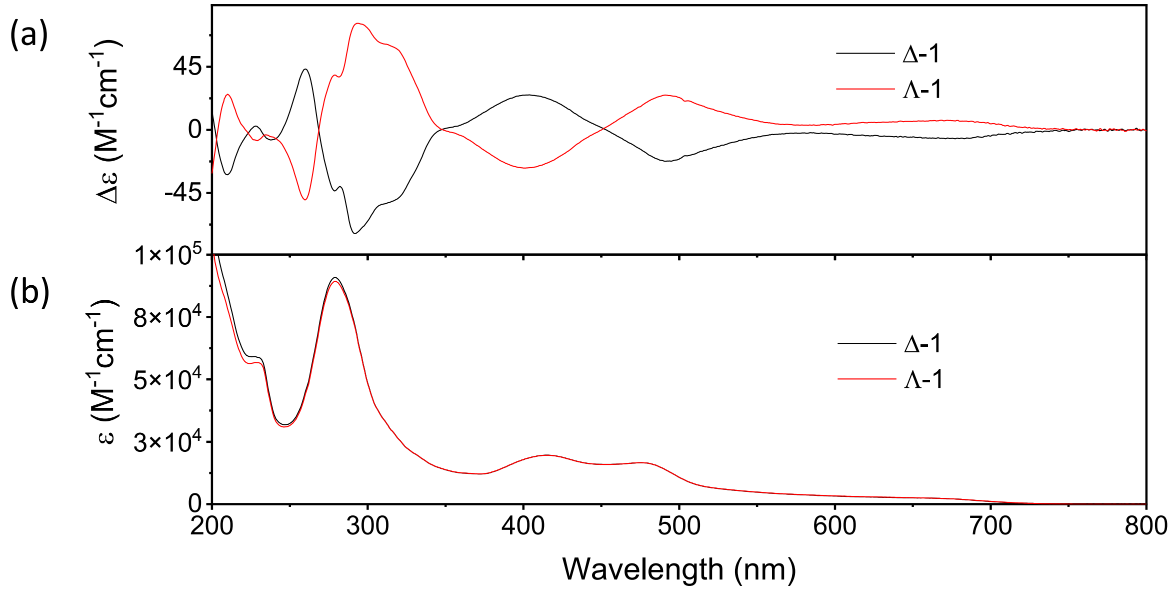

(c)

Δε M^{-1} cm^{-1}	Δε(292 nm)	Δε(401 nm)	Δε(492 nm)	Δε(673 nm)
Δ	-75	26	-23	-6
Λ	76	-27	24	6

Figure S11: (a) UV-Vis spectrum of the Δ-1 and Λ-1 enantiomers in water. (b) CD spectrum of the enantiomers (c) summary of the optical properties.

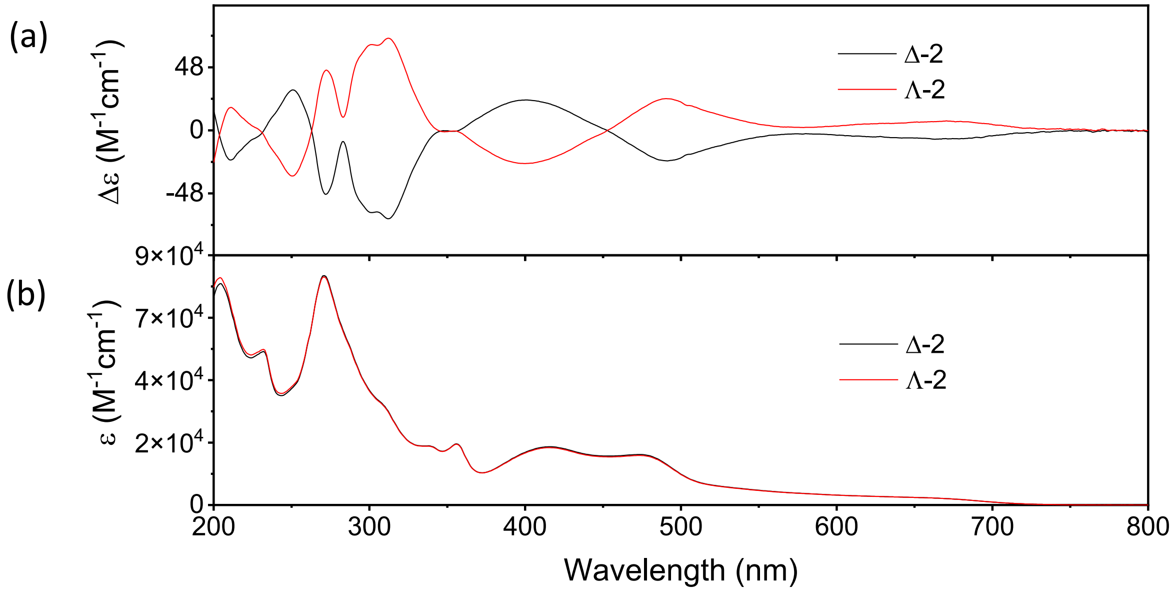

(c)

Δε M^{-1} cm^{-1}	Δε(272 nm)	Δε(312 nm)	Δε(401 nm)	Δε(491 nm)
Δ	-49	-67	24	-23
Λ	46	70	-25	24

Figure S12: (a) UV-Vis spectrum of the Δ-2 and Λ-2 enantiomers in water. (b) CD spectrum of the enantiomers. (c) summary of the optical properties.

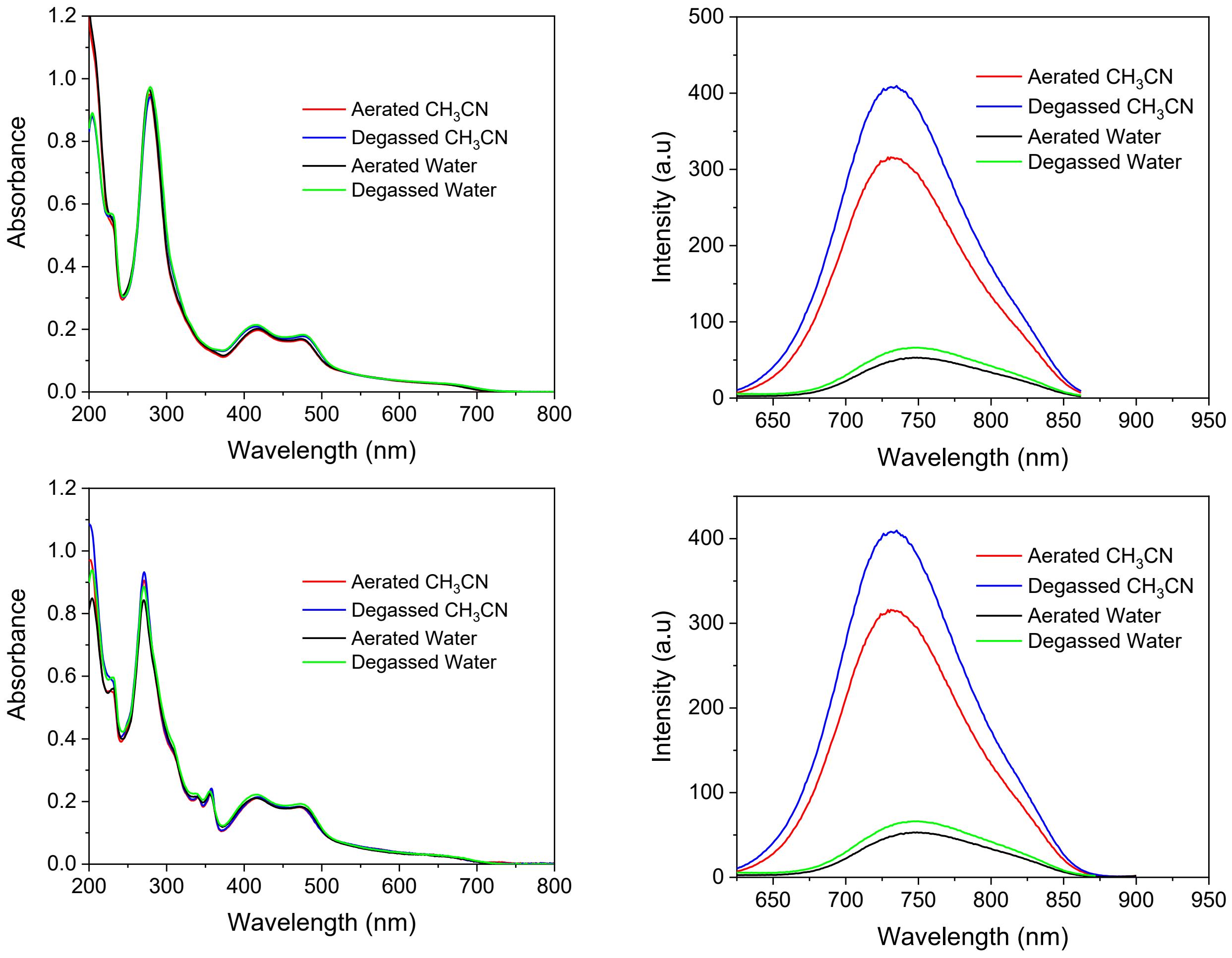

Figure S13: UV-Vis absorbance and emission spectra of (upper) 9.6 μM [1^{2+}] and (lower) 10.8 μM [2^{2+}] in water and MeCN at 298 K (λ_{ex} = 465 nm).

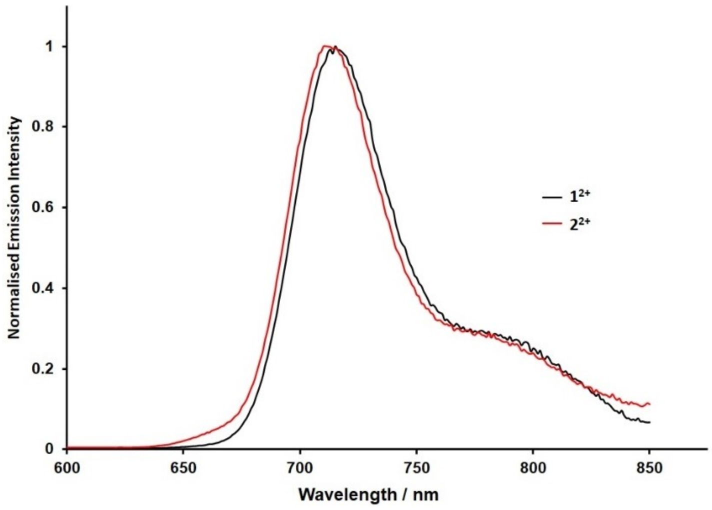

Figure S14: Normalized photoluminescence spectrum recorded for [**1**$^{2+}$][PF$_6$]$_2$ and [**2**$^{2+}$][Cl]$_2$ in a 4:1 EtOH/MeOH glass at 77K (λ_{ex} = 480 nm).

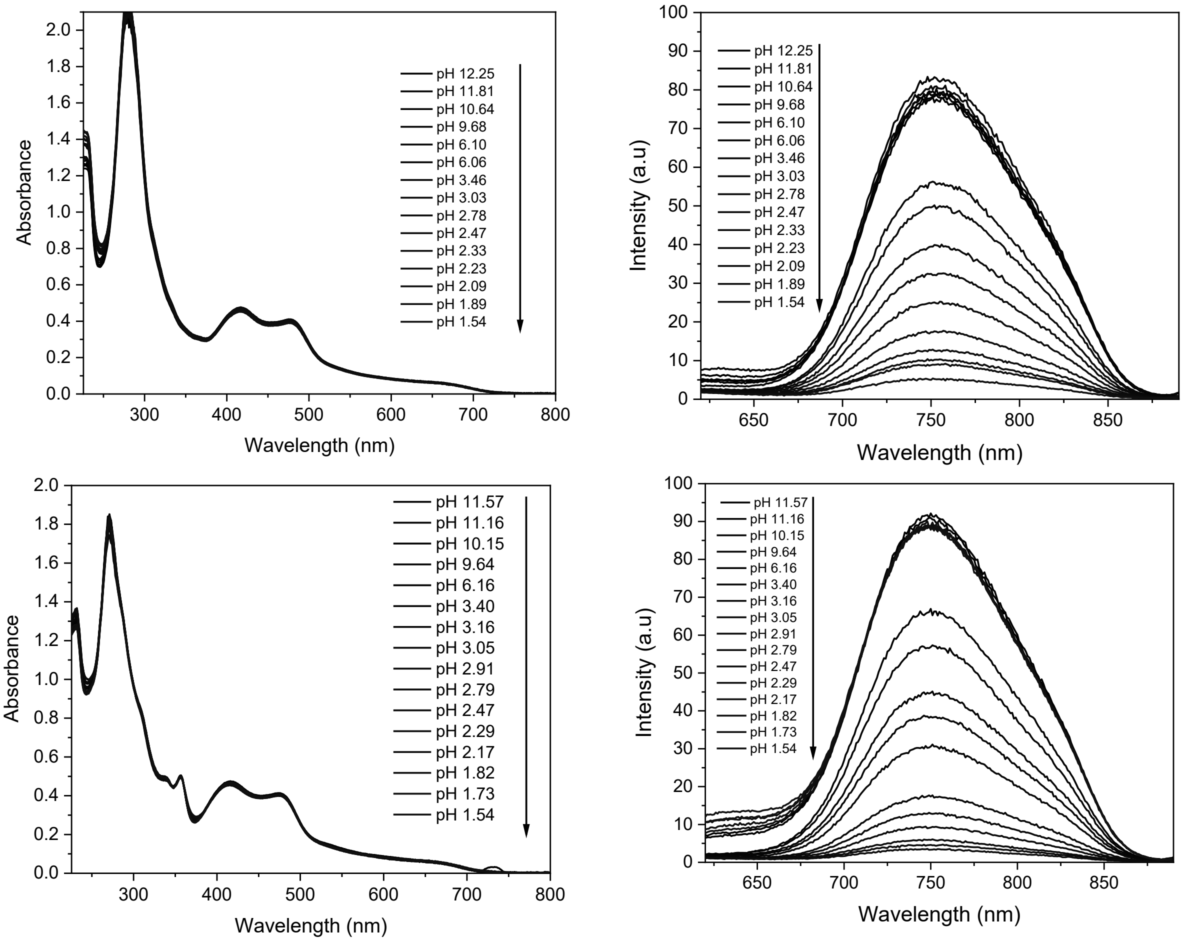

Figure S15: UV-Vis absorbance and emission spectra of [**1**$^{2+}$] (upper) and [**2**$^{2+}$] (lower) at various pH conditions in 1M NaCl buffer at 298 K (λ_{ex} = 465 nm).

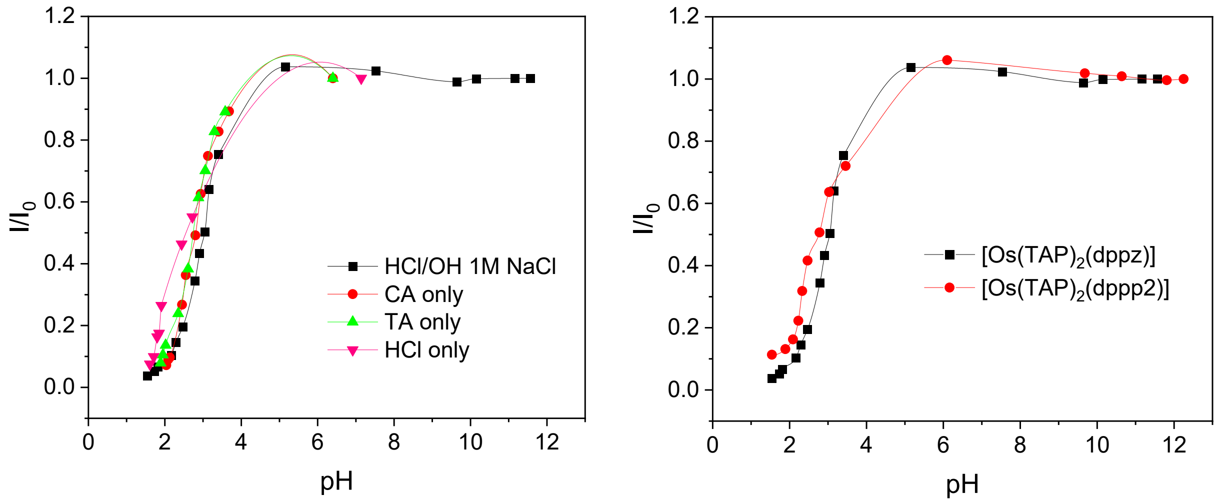

Figure S16: Comparative changes in emission spectra of [1^{2+}] and [2^{2+}] at various pH conditions in 1M NaCl buffer at 298 K (λ_{ex} = 465 nm). TA = tartaric acid, CA = citric acid

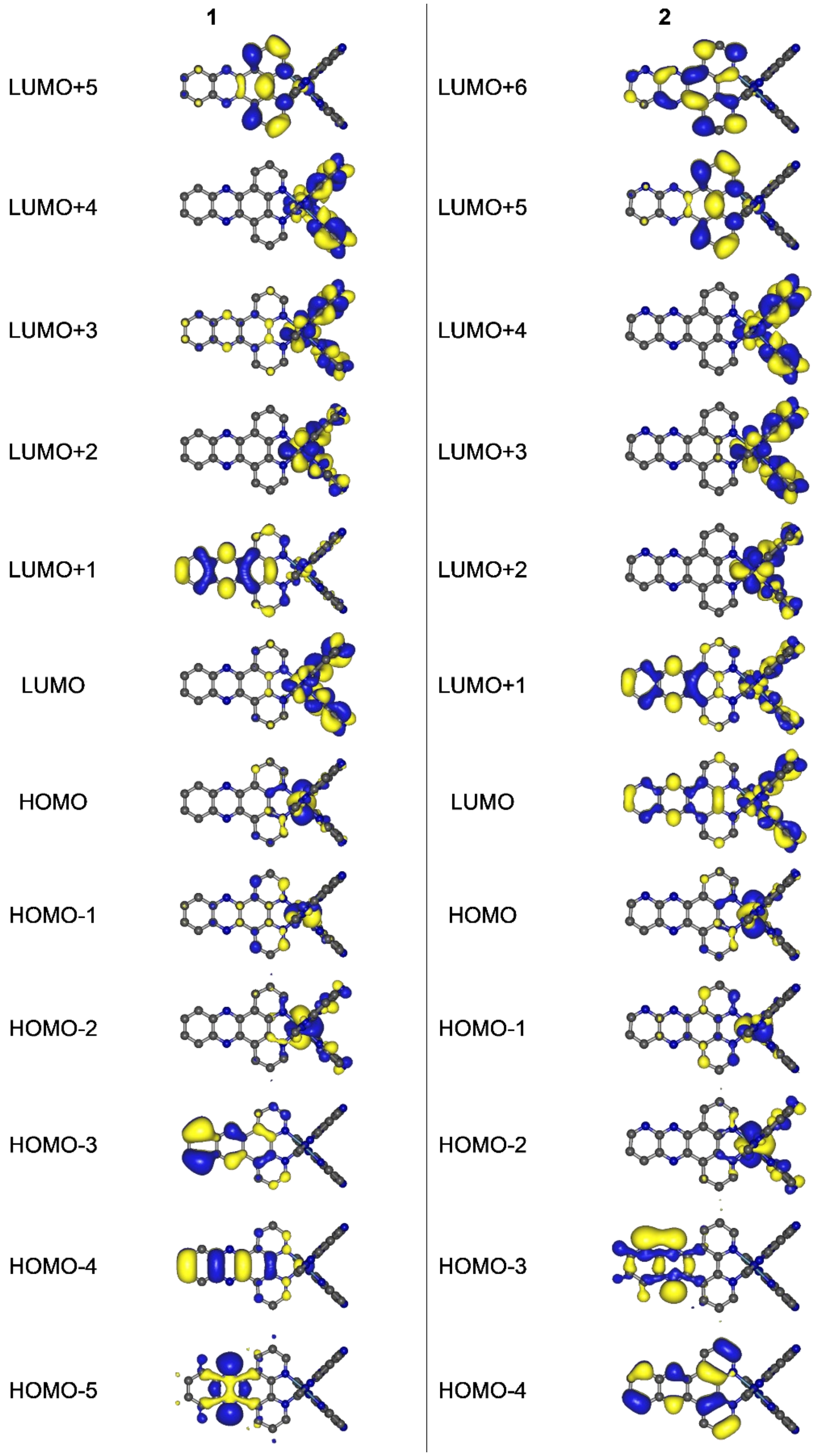

Figure S17: Plots of ground state molecular orbitals of $[Os(TAP)_2(dppz)]^{2+}$ and $[Os(TAP)_2(dppp2)]^{2+}$ in acetonitrile (COSMO-SMD) (isosurfaces set at 0.02 a.u).

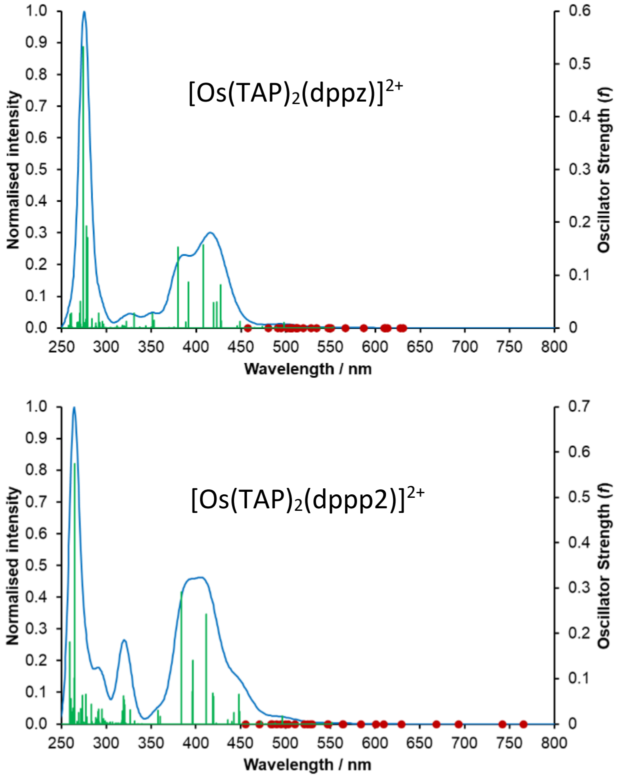

Figure S18: TDDFT calculated UV-visible absorption spectra from singlet state vertical excitations for $[Os(TAP)_2(dppz)]^{2+}$ and $[Os(TAP)_2(dppp2)]^{2+}$ in acetonitrile (COSMO-SMD) (green line) with Guassian line broadening set to 0.1 eV FWHM (blue line). Spin-forbidden ground state to triplet state vertical excitations at wavelengths longer than 450 nm indicated with red circles.

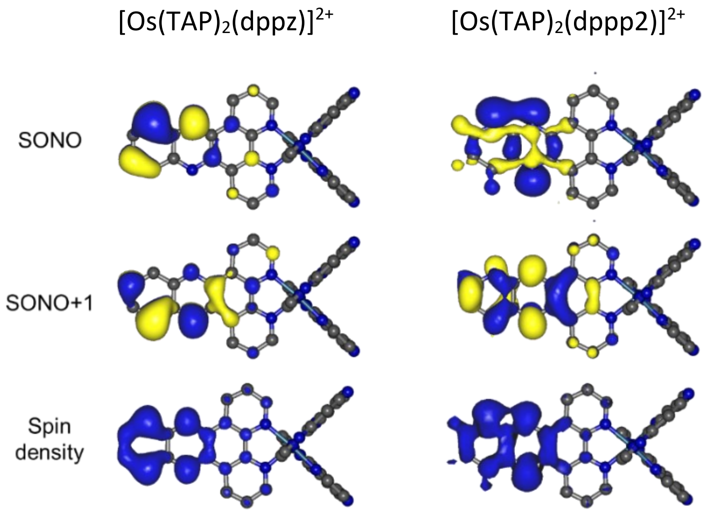

Figure S19: Plots of singly occupied natural orbitals of $[Os(TAP)_2(dppz)]^{2+}$ and $[Os(TAP)_2(dppp2)]^{2+}$ in acetonitrile (COSMO-SMD) (isosurfaces set at 0.02 a.u) and spin density for the two complexes (isosurfaces set at 0.001 a.u.).

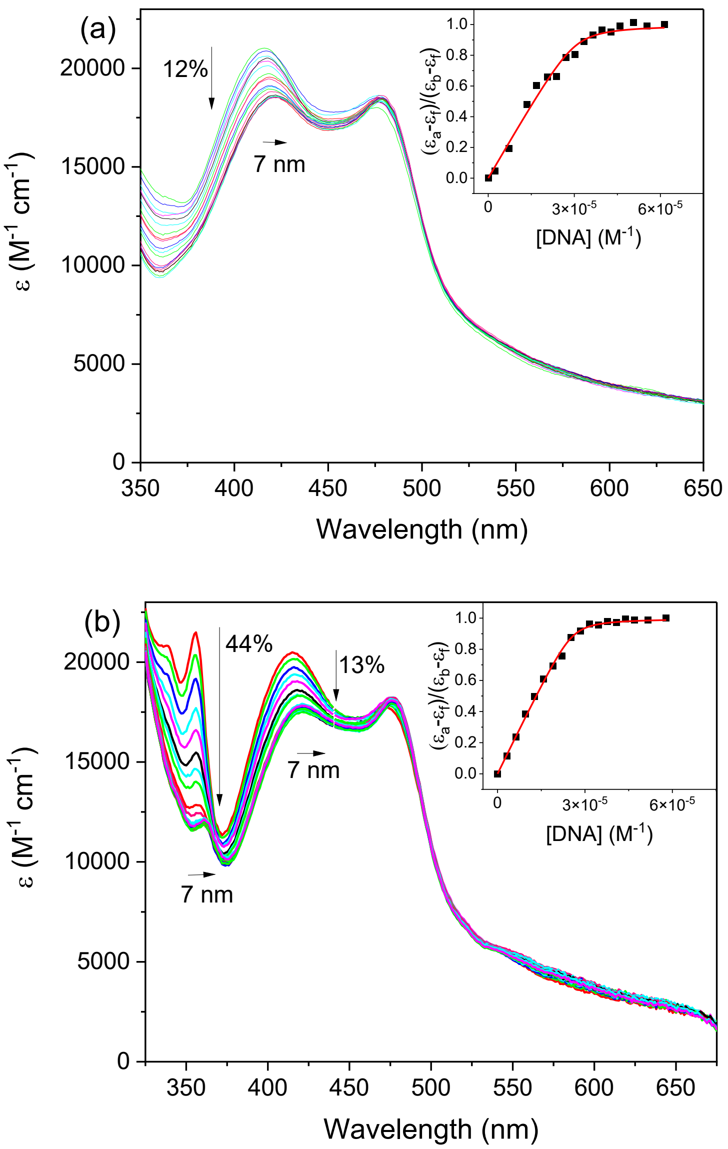

Figure S20: Absorbance spectra of (a) Λ–1 (9.5 μM) and (b) Λ-2 (9.34 μM) titrated against increasing concentrations of st-DNA (0 → 0.24mM) in 20 mM phosphate buffer at pH 7.0 (λ_{ex} = 465 nm). Insets show Bard binding fits of absorbance band at 415 nm.

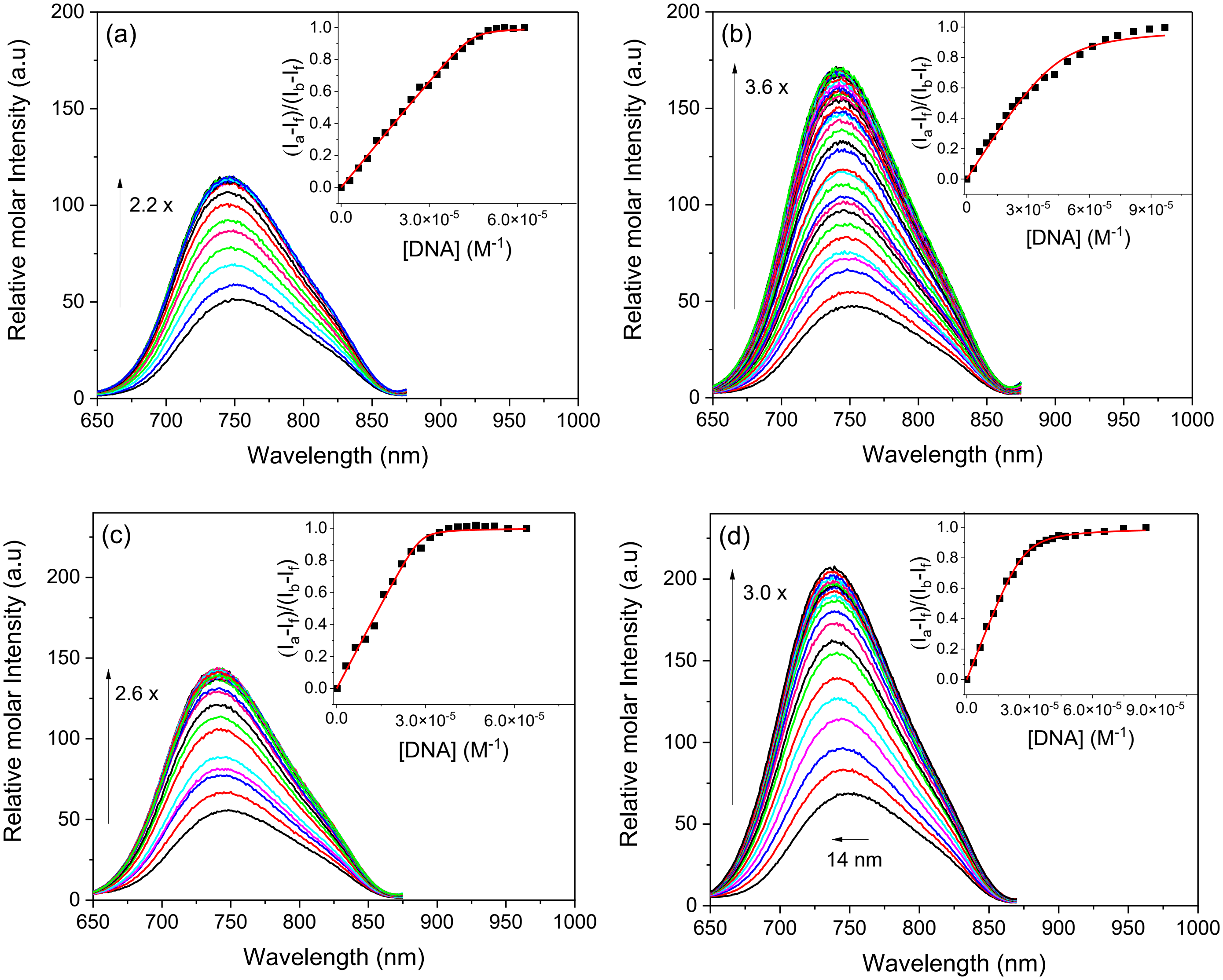

Figure S21: luminescence spectra of (a) Δ–**1**$^{2+}$ (11.2 μM), (b) Λ–**1** (9.5 μM), (c) Δ–**2**$^{2+}$ (12.2 μM) and (d) Λ-**2** (9.34 μM) titrated against increasing concentrations of st-DNA (0 → 0.24 mM) in 20 mM phosphate buffer at pH 7.0. Insets showing Bard binding fits to emission band at 750 nm.

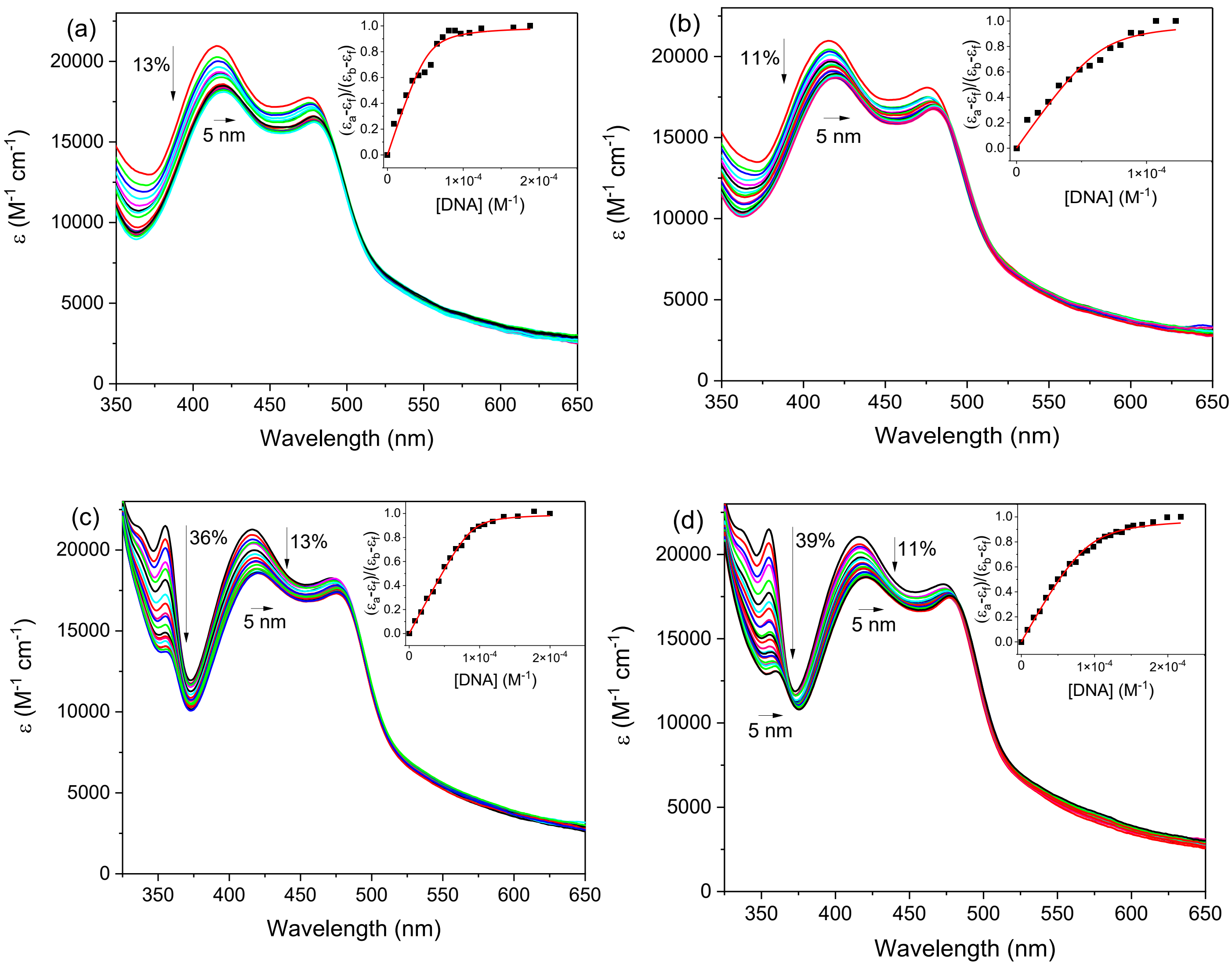

Figure S22: Absorbance spectra of (a) Δ-**1** (8.4 μM) (b) Λ-**1** (9.4 μM) (c) Δ-**2** (11.5 μM) and (d) Λ-**2** (10.9 μM) titrated against increasing concentrations of **GC** DNA (0 → 0.42 mM) in 20 mM phosphate buffer at pH 7.0 λ_{ex} = 465 nm. Bard fitting to 415 nm for (a) and (b) and 355 nm for (c) and (d).

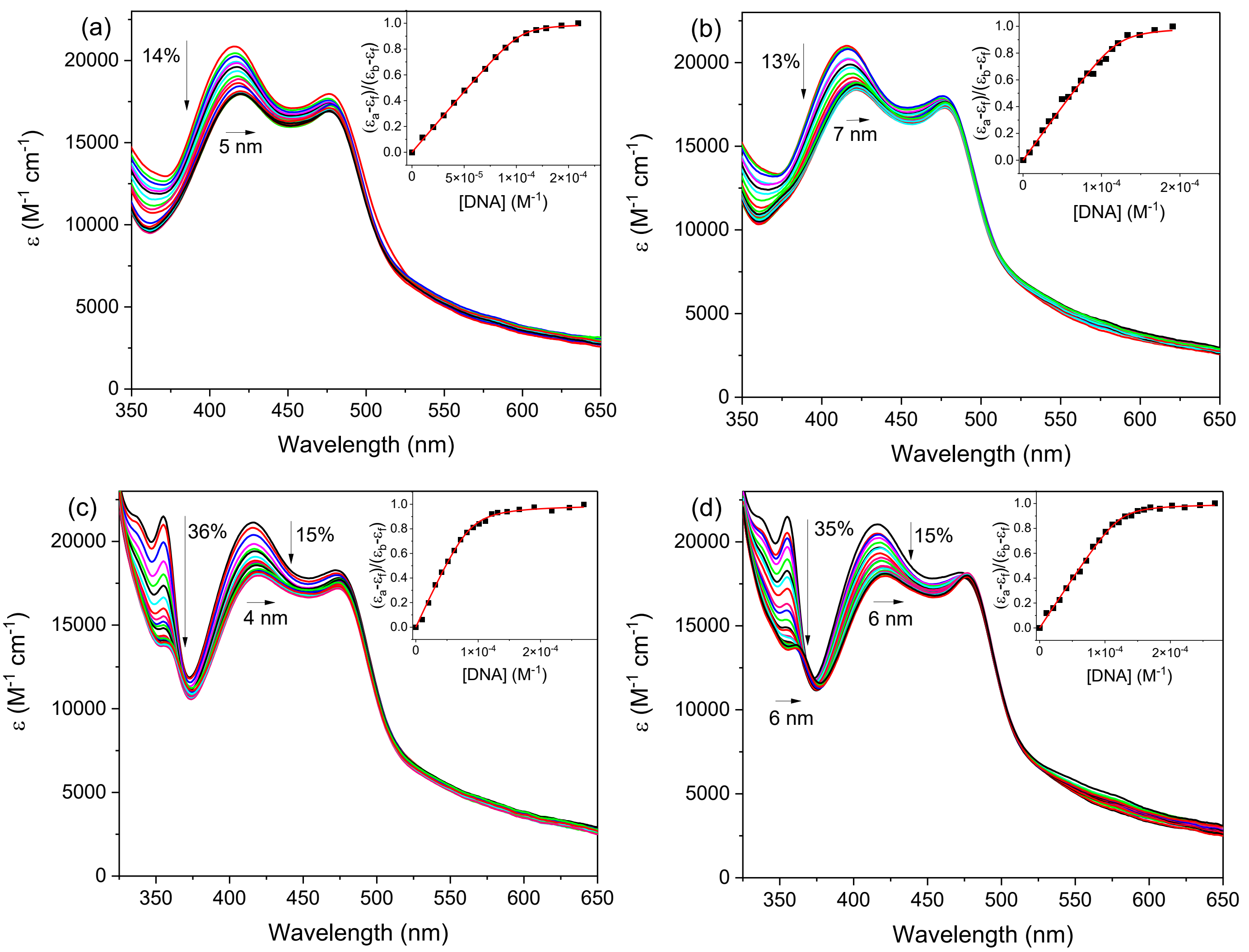

Figure S23: Absorbance spectra of (a) Δ-**1** (11.6 μM) (b) Λ-**1** (10.1 μM) (c) Δ-**2** (9.8 μM) and (d) Λ-**2** (11.1 μM) titrated against increasing concentrations of **AT** DNA (0 → 0.52 mM) in 20 mM phosphate buffer at pH 7.0 λ_{ex} = 465 nm. Bard fitting to 415 nm for (a) and (b) and 355 nm for (c) and (d).

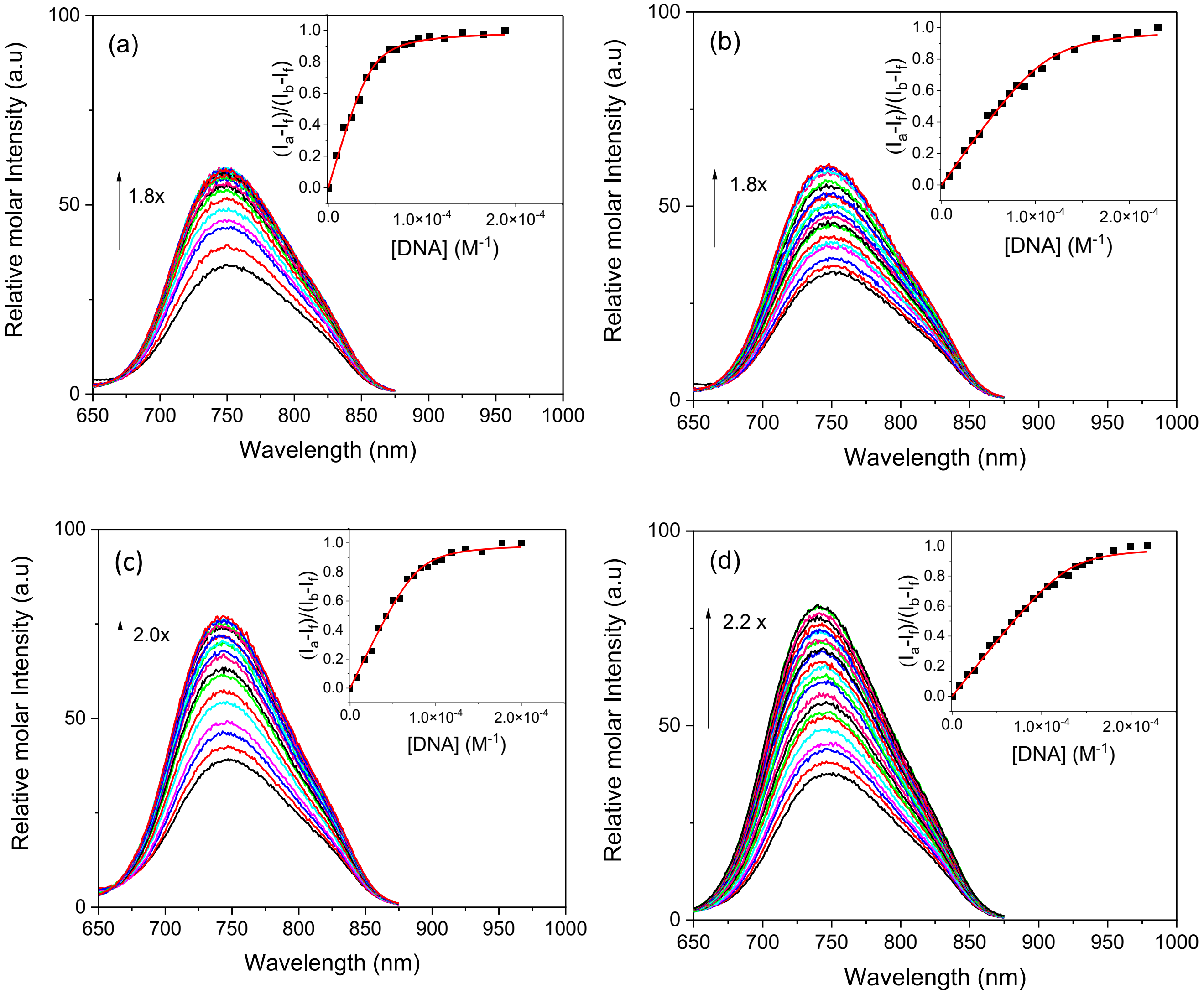

Figure S24: luminescence spectra of (a) Δ-**1** (8.4 μM) (b) Λ-**1** (9.4 μM) (c) Δ-**2** (11.5 μM) and (d) Λ-**2** (10.9 μM) titrated against increasing concentrations of GC DNA(0 → 0.42 mM) in 20 mM phosphate buffer at pH 7.0 (λ_{ex} = 465 nm). Insets showing Bard binding fits to emission band at 750 nm.

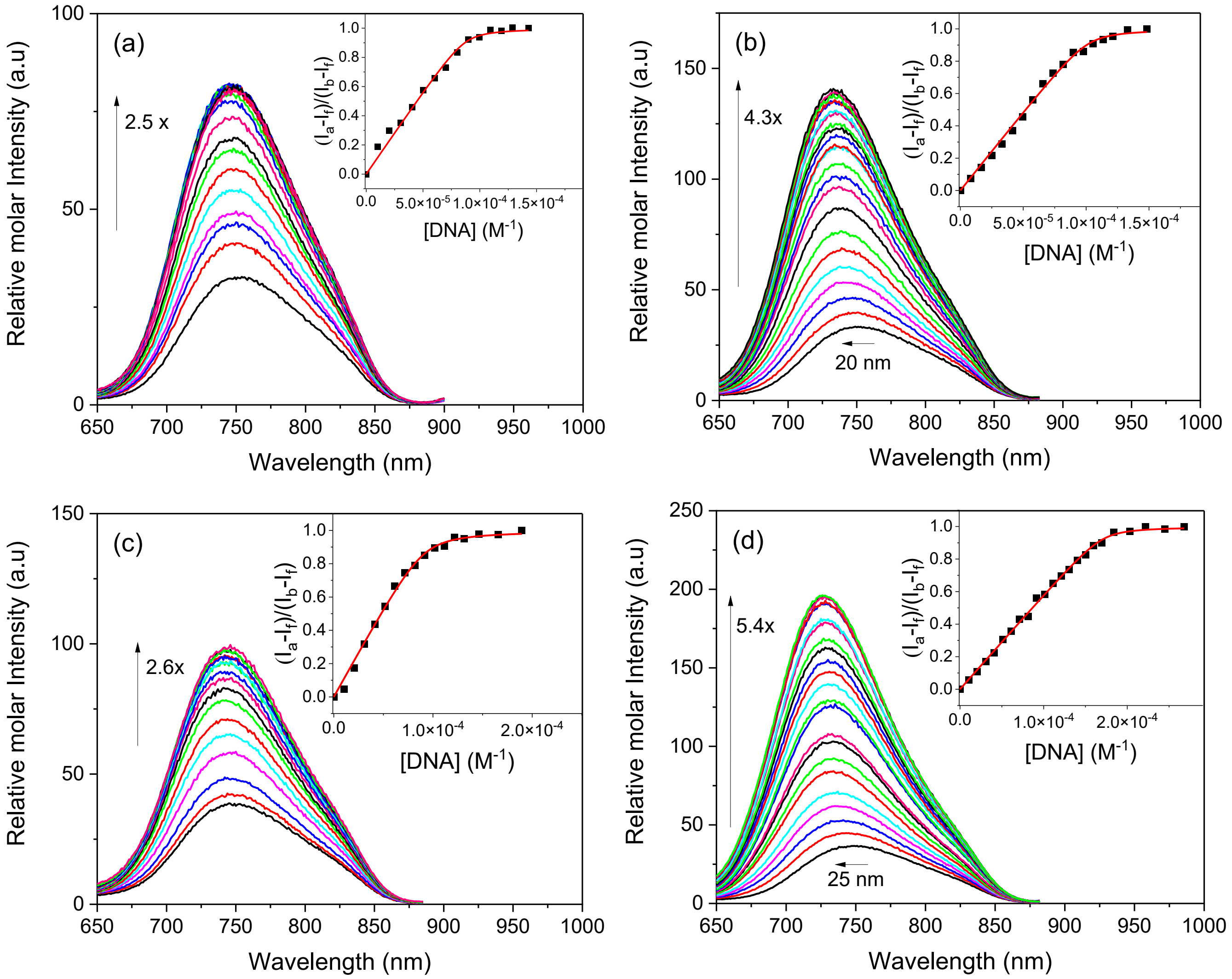

Figure S25: luminescence spectra of (a) Δ-**1** (11.6 μM) (b) Λ-**1** (10.1 μM) (c) Δ-**2** (9.8 μM) and (d) Λ-**2** (11.1 μM) titrated against increasing concentrations of AT DNA (0 → 0.52 mM) in 20 mM phosphate buffer at pH 7.0 (λ_{ex} = 465 nm). Insets showing Bard binding fits to emission band at 750 nm.

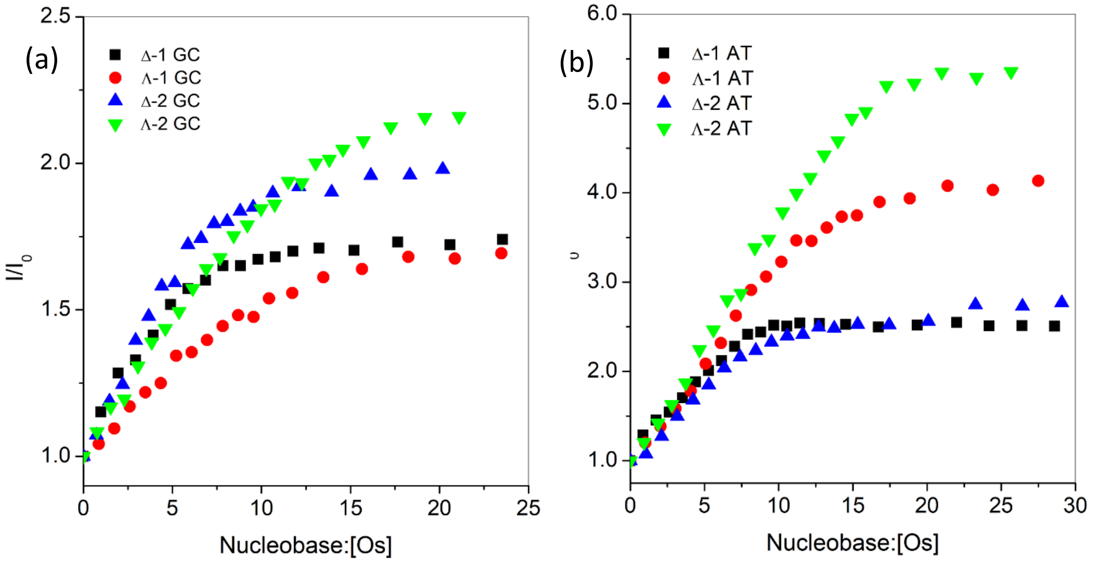

Figure S26: Luminescence trends observed for complexes **1** and **2** in the presence of increasing concentrations of (a) GC and (b) AT DNA in 20 mM phosphate buffer at pH 7.0 λ_{ex} = 465 nm.

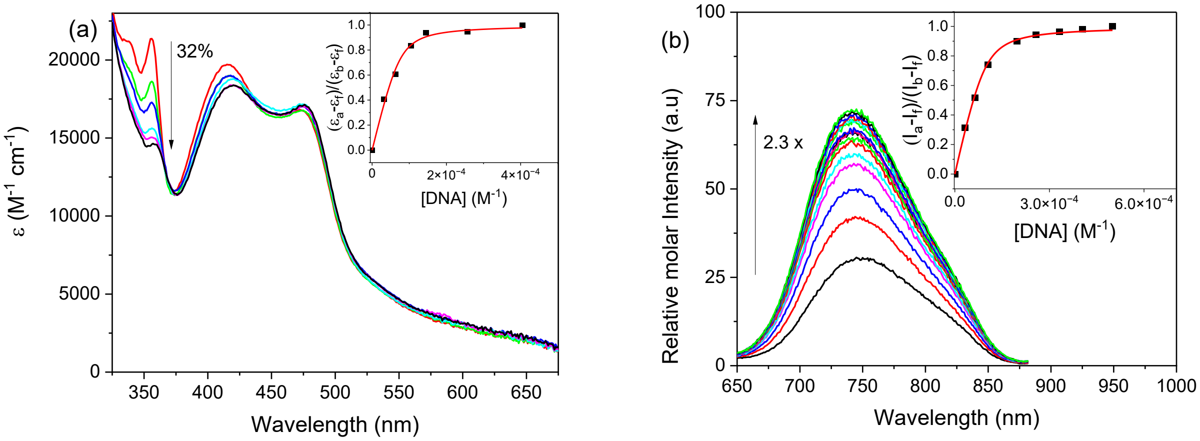

Figure S27: (a) UV absorbance and (b) luminescence spectra of Λ-**2** (8.7 μM) titrated against increasing concentrations of CCG-GAT-CCG-G DNA (0 → 0.82 mM) in 20 mM phosphate buffer at pH 7.0 (λ_{ex} = 465 nm). Insets showing Bard binding fits.

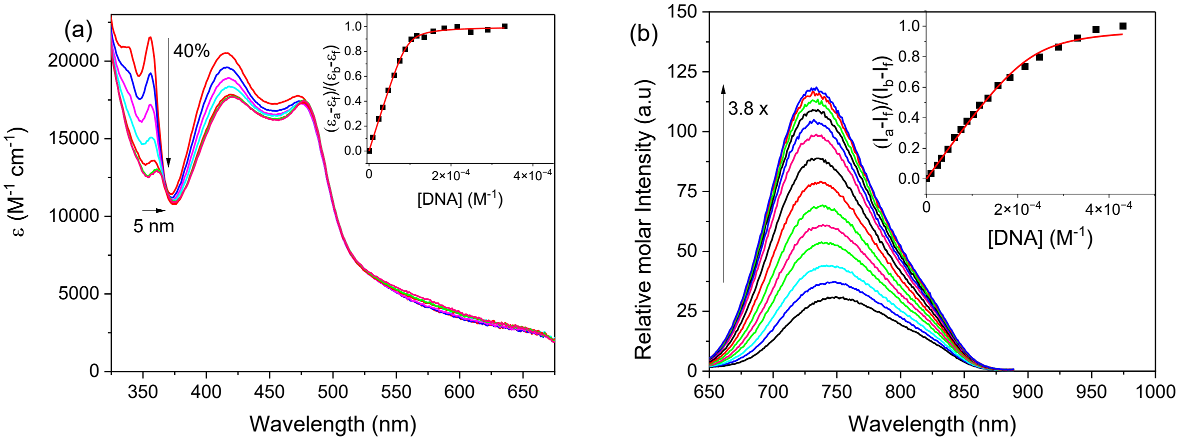

Figure S28: (a) UV absorbance and (b) luminescence spectra of Λ-**2** (14.7 μM) titrated against increasing concentrations of CCG-GTA-CCG-G DNA (0 → 0.61 mM) in 20 mM phosphate buffer at pH 7.0 (λ_{ex} = 465 nm). Insets showing Bard binding fits.

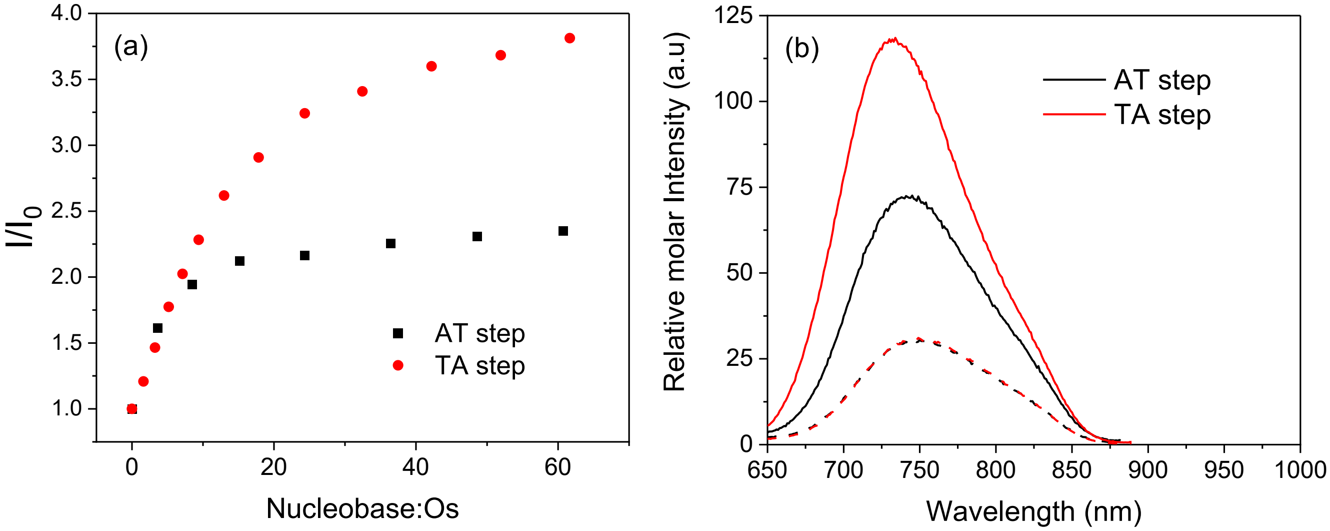

Figure S29: Comparative luminescence trends observed for complex Λ-**2** (a) in the presence of increasing concentrations of CCG-G**TA**-CCG-G and CCG-G**AT**-CCG-G and (b) emission recorded at 60:1 [Nucleobase]:[Os] in 20 mM phosphate buffer at pH 7.0 λ_{ex} = 465 nm.

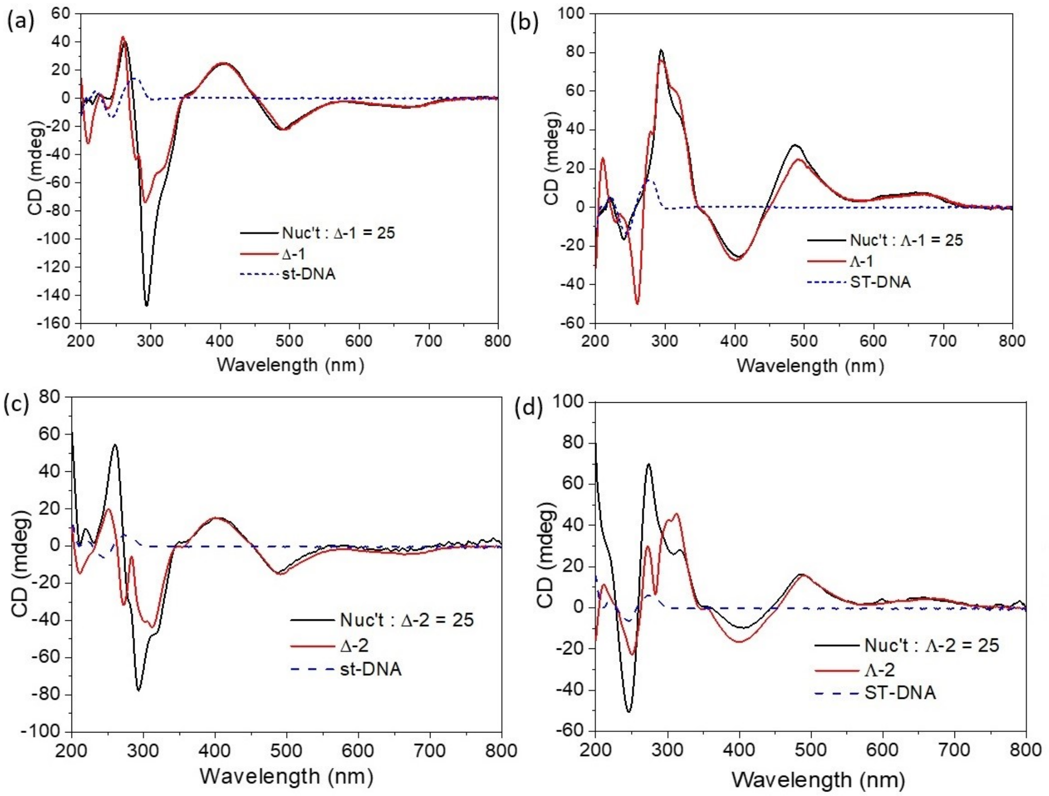

Figure S30: Circular dichroism of (a) Δ-1 and (b) Λ-1 (c) Δ-2 and (d) Λ-2 bound to st-DNA (400 μM) ([Nucleobase]:[Os]=5) in 20 mM phosphate buffer at pH 7.0 (black line 400 μM st-DNA).

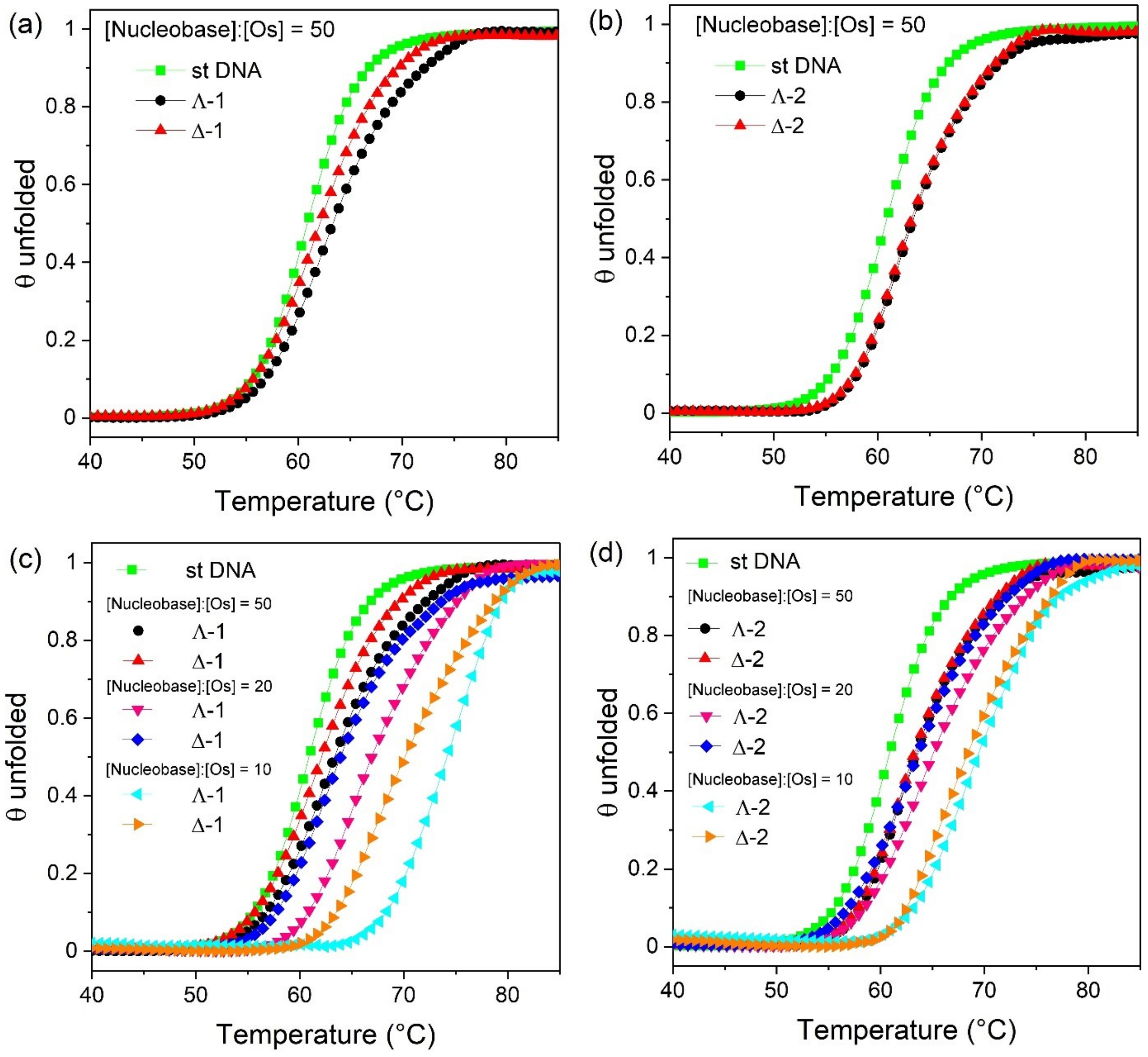

Figure S31: Fraction of DNA folded as a function of temperature for enantiomers for (a) **1** and (b) **2** bound to st-DNA at [Nucleobase]:[Os] = 50. Comparison of fraction of DNA folded as a function of temperature for enantiomers of (c) **1** and (d) **2** at different [Nucleobase]:[Os] ratios of 50, 20 and 10. For all, st-DNA (150 μM) in 1 mM phosphate buffer and 2 mM NaCl pH 7.0.

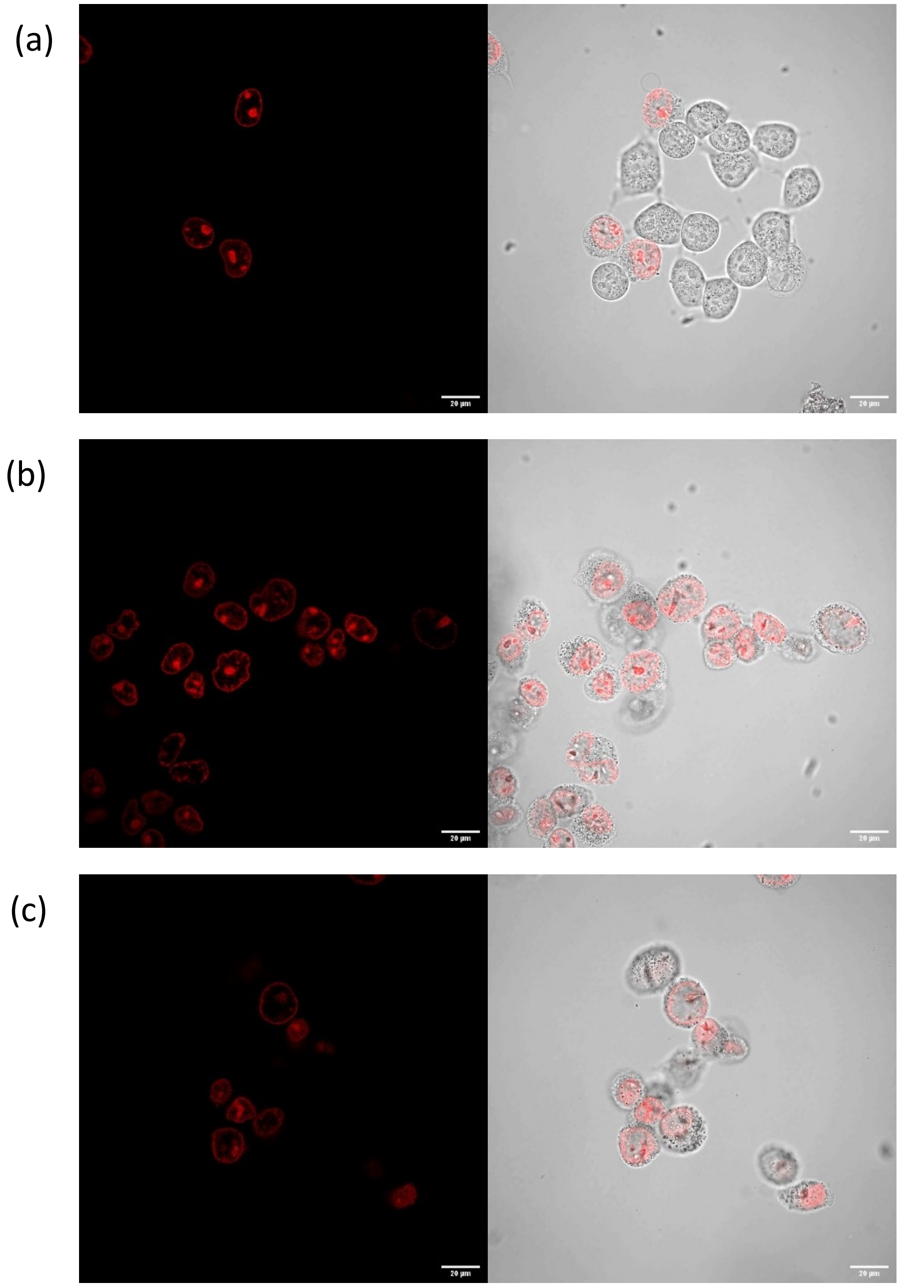

Figure S32: Confocal fluorescence (λ_{ex} = 405 nm/ $\lambda_{detection}$ = 700-800 nm) and transmission light images for HeLa Kyoto cells incubated with **1** recorded at (a) 60 min, (b) 120 min and (c) 180 min after complex addition. Scale bars are 20 µm.

(a)
(b)
(c)

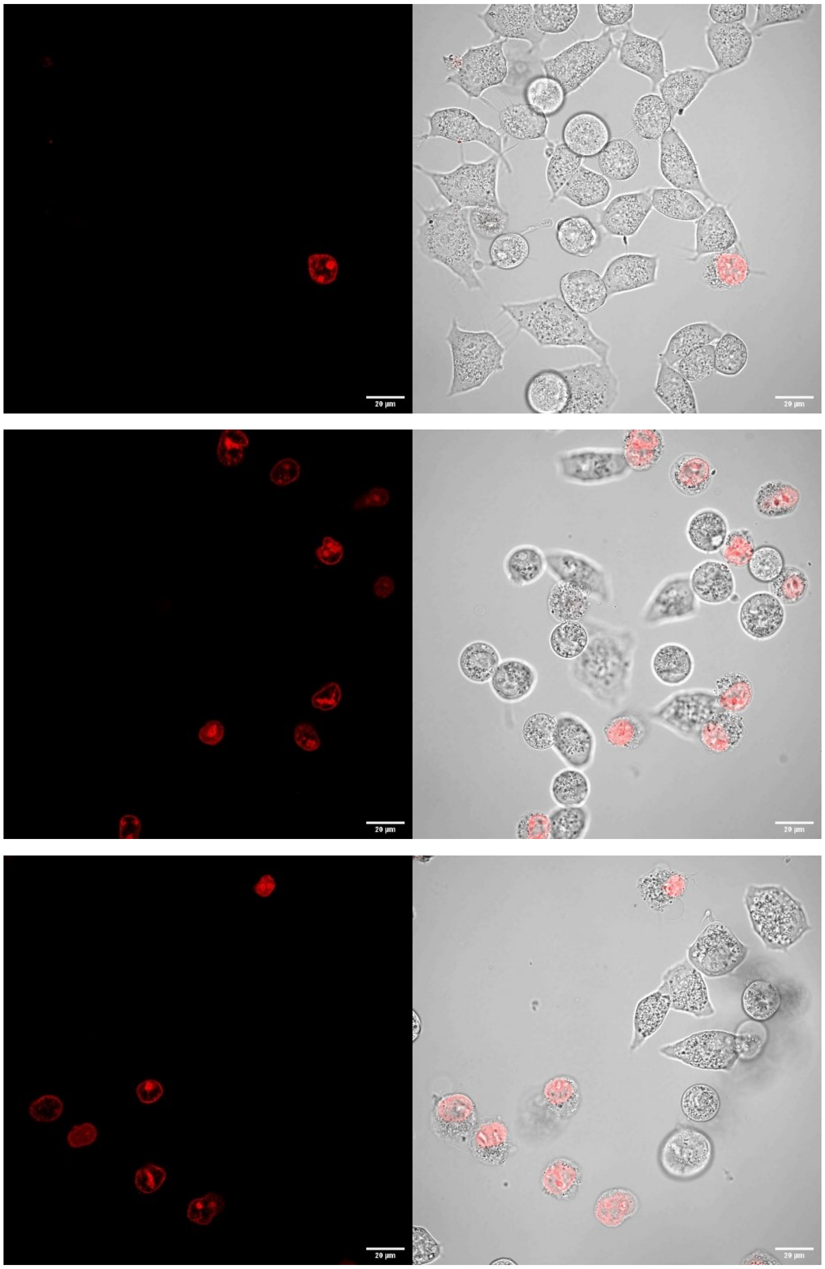

Figure S33: Confocal fluorescence (λ_{ex} = 405 nm/ $\lambda_{detection}$ = 700-800 nm) and transmission light images for HeLa Kyoto cells incubated with **2** recorded at (a) 60 min, (b) 120 min and (c) 180 min after complex addition. Scale bars are 20 µm.

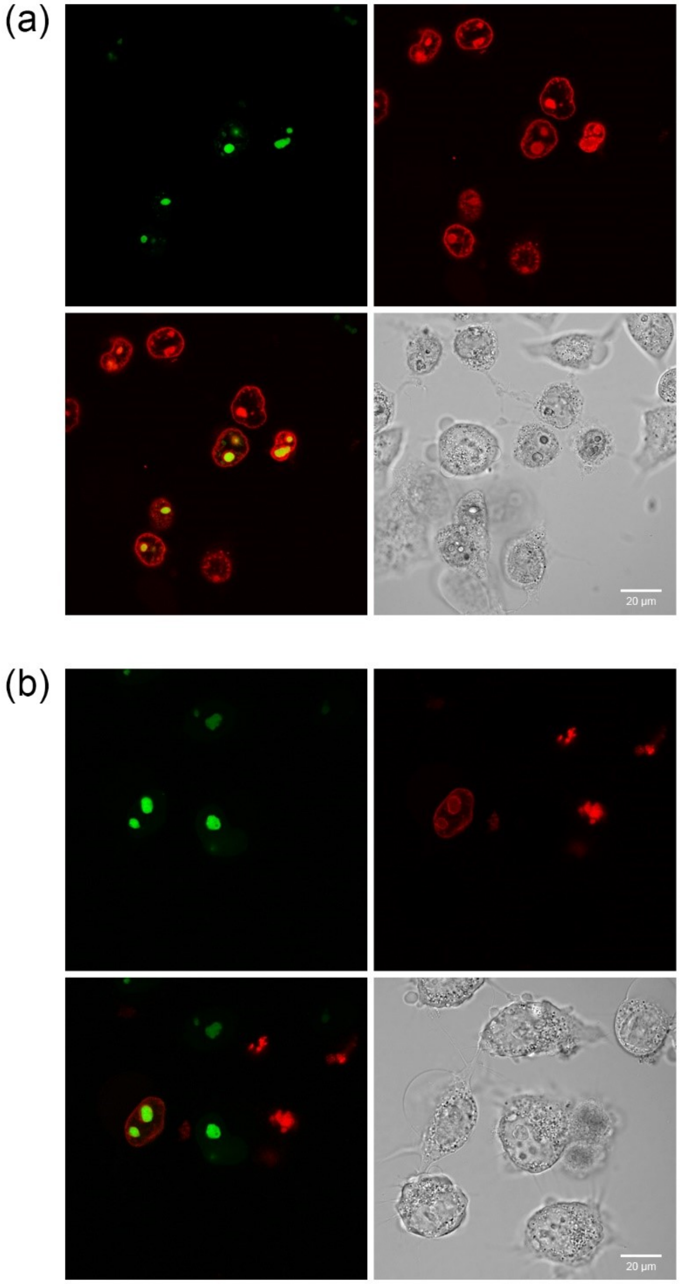

Figure S34: Confocal fluorescence and transmission light images for HeLa Kyoto cells incubated with (a) **1** or (b) **2** for 120 min. Cells were transfected with REXO4-EYFP the day prior to addition of complexes. REXO4-EYFP is shown in green, and complexes in red. Also shown is the merged image of REXO4-EYFP and complexes, as well as the transmitted light image.

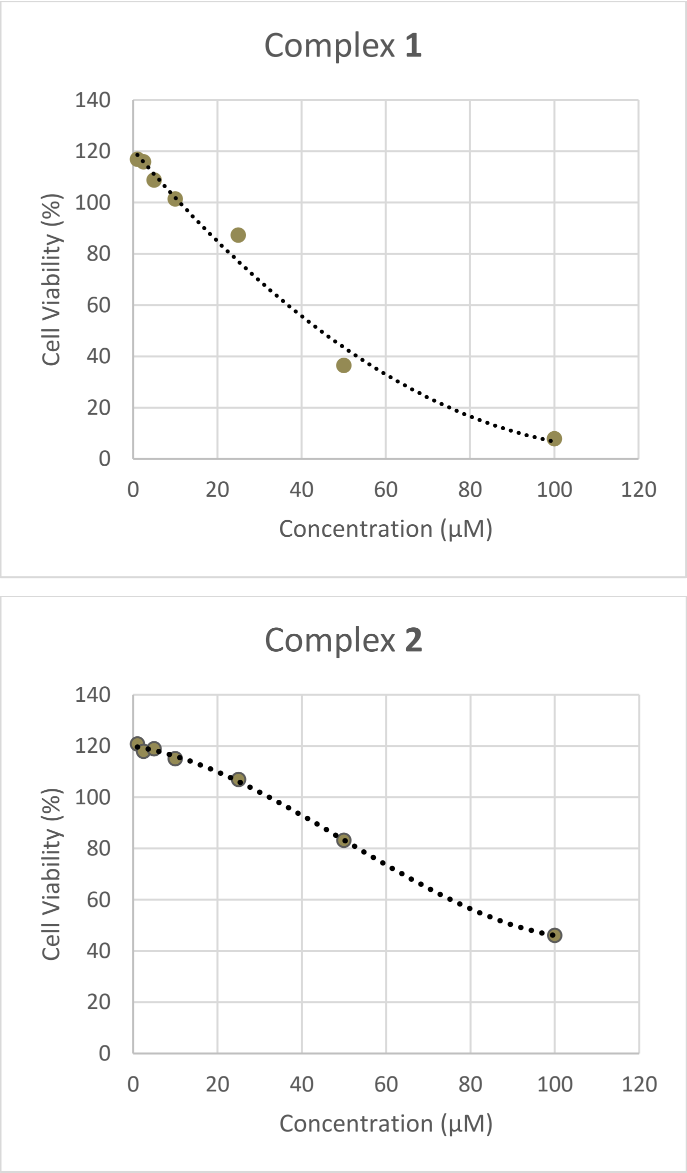

Figure S35: Cell Viability assay. HeLa Kyoto cells were incubated for 2.5 h with various concentrations of **1** or **2** and then incubated with CellTiter-Glo reagents. Luminescence, as a measure of cell viability, was recorded in a plate reader. Results are shown as % cell viability compared to control cells, not incubated with complexes. IC50 values were determined to be 45 µM for **1** and 90 µM for **2**.

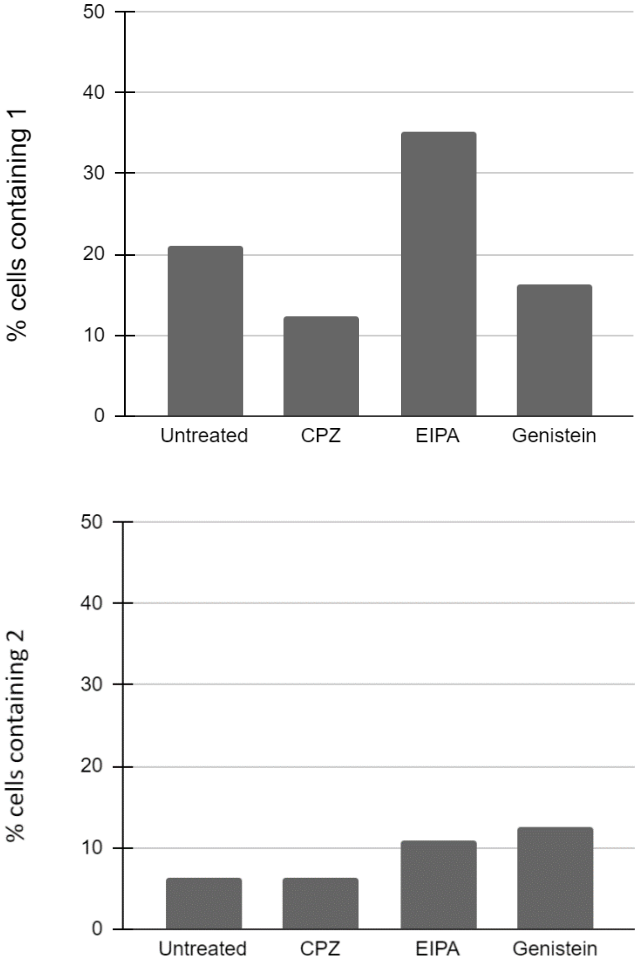

Figure S36: Endocytic inhibitor assay. HeLa Kyoto cells were incubated for 1 h with various inhibitors of endocytosis, specifically chlorpromazine (CPZ) (1 µg/ml), 5-(N-Ethyl-N-isopropyl)-Amiloride (EIPA) (300 µM) or genistein (300 µM). After this, 50 µM of **1** or **2** were added and then incubated for a further 2 h. Cells were imaged live by confocal microscopy and the % cells in the population containing internalized **1** or **2** was determined.

Additional Tables

Table S2. Photoluminescence pH Studies for rac-[Os(Tap)$_2$(dppp2)][Cl]$_2$

pH	Measured pH	Φ_{em} (%)	τ_{em} (ns)	Fit (χ^2)
3	3.00	0.03	6.5 (18%)<br>377 (82%)	1.006
5	5.04	0.06	13.2 (40%)<br>29 (36%)<br>368 (24%)	1.052
5.5	5.54	0.07	17 (49%)<br>41 (46%)<br>400 (5%)	1.070
7	7.08	0.14	20 (13%)<br>66 (54%)<br>262 (33%)	1.108
8	7.90	0.21	28 (9%)<br>97 (46%)<br>519 (45%)	1.004
9	8.94	0.29	38 (10%)<br>122 (44%)<br>807 (46%)	1.036

All data for aerated aqueous, buffered, solutions at room temperature.

Table S3. Data for selected vertical excitations of $[Os(TAP)_2(dppz)]^{2+}$ in acetonitrile (COSMO-SMD) from TDDFT calculations.

State	Energy / cm^{-1}	λ / nm	f_{osc}	Composition	Character
S_1	18089	553	0.0023	HOMO→LUMO (96 %)	$MLCT_{TAP}$
S_2	18846	531	0.00023	HOMO→LUMO+2 (81 %)	$MLCT_{TAP}$
S_3	20093	498	0.014	HOMO-1→LUMO (85 %)	$MLCT_{TAP}$
S_8	22264	449	0.016	HOMO-1→LUMO+4 (72 %)	$MLCT_{TAP}$
S_{10}	23364	428	0.018	HOMO→LUMO+5 (54 %)<br>HOMO→LUMO+1 (36 %)	$MLCT_{dppz}$
S_{11}	23411	427	0.11	HOMO-1→LUMO+1 (50 %)<br>HOMO-1→LUMO+3 (37 %)	$MLCT_{dppz}$/<br>$MLCT_{TAP}$
S_{12}	23646	423	0.029	HOMO-2→LUMO+4 (49 %)<br>HOMO-2→LUMO+2 (18 %)	$MLCT_{TAP}$
S_{13}	23647	423	0.066	HOMO-2→LUMO+1 (38 %)<br>HOMO-2→LUMO+5 (20 %)	$MLCT_{dppz}$
S_{14}	23851	419	0.064	HOMO-2→LUMO+3 (52 %)<br>HOMO-2→LUMO+1 (15 %)	$MLCT_{dppz}$/<br>$MLCT_{TAP}$
S_{15}	24509	408	0.21	HOMO-2→LUMO+3 (30 %)<br>HOMO-2→LUMO (18 %)<br>HOMO-2→LUMO+1 (17 %)<br>HOMO-1→LUMO+2 (15 %)	$MLCT_{dppz}$/<br>$MLCT_{TAP}$
S_{16}	25543	392	0.12	HOMO-1→LUMO+5 (47 %)<br>HOMO→LUMO+6 (18 %)<br>HOMO-1→LUMO+1 (13 %)	$MLCT_{dppz}$
S_{17}	25736	389	0.014	HOMO-2→LUMO+5 (68 %)<br>HOMO-2→LUMO+1 (23 %)	$MLCT_{dppz}$
S_{18}	26359	379	0.21	HOMO→LUMO+6 (70 %)	$MLCT_{dppz}$
S_{22}	28316	353	0.019	HOMO-2→LUMO+6 (84 %)	$MLCT_{dppz}$
S_{23}	28498	351	0.036	HOMO-3→LUMO+1 (52 %)<br>HOMO-1→LUMO+6 (23 %)	LC_{dppz}/$MLCT_{dppz}$
S_{28}	30243	331	0.037	HOMO-4→LUMO+1 (48 %)<br>HOMO-3→LUMO+6 (42 %)	LC_{dppz}
S_{31}	31037	322	0.017	HOMO-3→LUMO+5 (81 %)	LC_{dppz}
S_{56}	33869	295	0.017	HOMO-1→LUMO+7 (46 %)<br>HOMO-11→LUMO (23 %)	$MLCT_{TAP}$/LC_{TAP}
S_{60}	34220	292	0.013	HOMO-7→LUMO+3 (24 %)<br>HOMO-6→LUMO+4 (15 %)<br>HOMO-6→LUMO+2 (23 %)	LC_{TAP}
S_{61}	34303	292	0.015	HOMO-7→LUMO+4 (32 %)<br>HOMO-6→LUMO+3 (32 %)	LC_{TAP}
S_{62}	34375	291	0.038	HOMO-8→LUMO+2 (32 %)	LC_{TAP}
S_{65}	34706	288	0.012	HOMO-9→LUMO+2 (28 %)<br>HOMO-10→LUMO (19 %)<br>HOMO-2→LUMO+7 (16 %)	LC_{TAP}/$MLCT_{TAP}$
S_{68}	35223	284	0.023	HOMO-1→LUMO+8 (34 %)	$MLCT_{TAP}$/

				HOMO-8→LUMO+3 (50 %)	$LLCT_{dppz \rightarrow TAP}$
S_{69}	35237	284	0.017	HOMO-8→LUMO+3 (49 %)<br>HOMO-1→LUMO+8 (35 %)	$MLCT_{TAP}$/<br>$LLCT_{dppz \rightarrow TAP}$
S_{71}	35869	279	0.23	HOMO-9→LUMO+3 (37 %)<br>HOMO-9→LUMO+1 (25 %)	LC_{TAP}/<br>$LLCT_{TAP \rightarrow dppz}$
S_{72}	35970	278	0.097	HOMO-9→LUMO+4 (35 %)<br>HOMO-9→LUMO+2 (13 %)	LC_{TAP}
S_{73}	36053	277	0.26	HOMO-10→LUMO+4 (42 %)<br>HOMO-2→LUMO+8 (13 %)	LC_{TAP}/$MLCT_{TAP}$
S_{74}	36070	277	0.022	HOMO-4→LUMO+6 (46%)<br>HOMO-8→LUMO+5 (17 %)<br>HOMO-6→LUMO+1 (11 %)	LC_{dppz}/<br>$LLCT_{TAP \rightarrow dppz}$
S_{76}	36250	276	0.013	HOMO-12→LUMO+2 (47 %)<br>HOMO-13→LUMO (19 %)	$LC_{TAP(n \rightarrow \pi^*)}$
S_{80}	36547	274	0.71	HOMO-3→LUMO+6 (27%)<br>HOMO-7→LUMO+5 (17 %)<br>HOMO-4→LUMO+1 (15 %)	LC_{dppz}/<br>$LLCT_{TAP \rightarrow dppz}$
S_{83}	36951	271	0.067	HOMO-7→LUMO+5 (56 %)<br>HOMO-2→LUMO+8 (15 %)	$LLCT_{TAP \rightarrow dppz}$/<br>$MLCT_{TAP}$
S_{84}	36978	270	0.037	HOMO-6→LUMO+5 (35%)<br>HOMO-10→LUMO+3 (18 %)<br>HOMO-9→LUMO+4 (12 %)	$LLCT_{TAP \rightarrow dppz}$/<br>$LC_{TAP}$
S_{86}	37299	268	0.016	HOMO-12→LUMO+4 (35%)<br>HOMO-13→LUMO+3 (18 %)<br>HOMO-2→LUMO+8 (12 %)	$LC_{TAP(n \rightarrow \pi^*)}$/<br>$MLCT_{TAP}$
S_{87}	37365	268	0.010	HOMO-12→LUMO+3 (26 %)<br>HOMO-13→LUMO+4 (22 %)	$LC_{TAP(n \rightarrow \pi^*)}$
S_{88}	37434	267	0.014	HOMO-12→LUMO+4 (21%)<br>HOMO-11→LUMO+3 (10 %)<br>HOMO-2→LUMO+8 (10 %)	$LC_{TAP(n \rightarrow \pi^*)}$/<br>$MLCT_{TAP}$
S_{95}	38313	261	0.037	HOMO→LUMO+11 (59 %)<br>HOMO→LUMO+12 (22 %)	$MLCT_{TAP}$
S_{98}	38482	260	0.035	HOMO-10→LUMO+5 (22 %)	$LLCT_{TAP \rightarrow dppz}$
T_1	15855	631	-	HOMO→LUMO (88 %)	$MLCT_{TAP}$
T_2	15940	627	-	HOMO-3→LUMO+4 (92 %)	$LLCT_{dppz \rightarrow TAP}$
T_3	16310	613	-	HOMO-3→LUMO+2 (94 %)	$LLCT_{dppz \rightarrow TAP}$
T_4	16363	611	-	HOMO→LUMO+2 (76 %)<br>HOMO→LUMO+4 (10 %)	$MLCT_{TAP}$
T_5	16403	610	-	HOMO-5→LUMO (89 %)	$LLCT_{dppz \rightarrow TAP(n \rightarrow \pi^*)}$
T_6	17052	587	-	HOMO-1→LUMO (38 %)<br>HOMO-2→LUMO+2 (26 %)<br>HOMO-2→LUMO+4 (10 %)	$MLCT_{TAP}$
T_7	17660	566	-	HOMO→LUMO+4 (51 %)<br>HOMO-2→LUMO+2 (32 %)	$MLCT_{TAP}$
T_8	18168	550	-	HOMO→LUMO+4 (67 %)	$MLCT_{TAP}$

				HOMO-1→LUMO+2 (11 %)	
T_9	18227	549	-	HOMO-2→LUMO+1 (40 %)<br>HOMO-2→LUMO+5 (35 %)	$MLCT_{dppz}$
T_{10}	18272	547	-	HOMO-1→LUMO (42 %)<br>HOMO-2→LUMO+2 (28 %)	$MLCT_{TAP}$
T_{11}	18723	534	-	HOMO-1→LUMO+2 (48 %)<br>HOMO-2→LUMO (36 %)	$MLCT_{TAP}$
T_{12}	18949	528	-	HOMO-2→LUMO+5 (44 %)<br>HOMO-2→LUMO+1 (27 %)	$MLCT_{dppz}$
T_{13}	19264	519	-	HOMO→LUMO+1 (32 %)<br>HOMO→LUMO+5 (21 %)<br>HOMO-5→LUMO+1 (21 %)	$MLCT_{dppz}$
T_{14}	19477	513	-	HOMO→LUMO+4 (28 %)<br>HOMO→LUMO+5 (28 %)<br>HOMO-3→LUMO+1 (24 %)	LC_{dppz}/$MLCT_{TAP}$/<br>$MLCT_{dppz}$
T_{15}	19540	512	-	HOMO→LUMO+1 (26 %)<br>HOMO→LUMO+5 (26 %)<br>HOMO-3→LUMO+1 (10 %)	$MLCT_{dppz}$/<br>LC_{dppz}
T_{16}	19557	511	-	HOMO-1→LUMO+4 (52 %)<br>HOMO-2→LUMO+3 (24 %)	$MLCT_{TAP}$
T_{17}	19736	507	-	HOMO-1→LUMO+3 (24 %)<br>HOMO-2→LUMO+4 (20 %)<br>HOMO-1→LUMO+1 (15 %)	$MLCT_{dppz}$/<br>$MLCT_{TAP}$
T_{18}	19763	506	-	HOMO-1→LUMO+1 (39 %)<br>HOMO-1→LUMO+5 (20 %)	$MLCT_{dppz}$
T_{19}	19800	505	-	HOMO-3→LUMO+3 (20 %)	$LLCT_{dppz\rightarrow TAP}$
T_{20}	19819	505	-	HOMO→LUMO+3 (40 %)<br>HOMO-3→LUMO+1 (35 %)	LC_{dppz}/$MLCT_{TAP}$
T_{21}	19946	501	-	HOMO-1→LUMO+5 (24 %)<br>HOMO-1→LUMO+1 (16 %)	$MLCT_{dppz}$

Table S4. Data for selected vertical excitations of $[Os(TAP)_2(dppp2)]^{2+}$ in acetonitrile (COSMO-SMD) from TDDFT calculations.

State	Energy / cm^{-1}	λ / nm	f_{osc}	Composition	Character
S_1	18126	552	0.0023	HOMO→LUMO (60 %)<br>HOMO→LUMO+1 (38 %)	$MLCT_{TAP/dppn2}$
S_2	18898	529	0.00022	HOMO→LUMO+2 (81 %)<br>HOMO→LUMO+4 (17 %)	$MLCT_{TAP}$
S_3	20141	497	0.015	HOMO-1→LUMO (57 %)<br>HOMO-1→LUMO+1 (35 %)	$MLCT_{TAP/dppn2}$
S_7	21151	473	0.0043	HOMO→LUMO+4 (62 %)<br>HOMO-2→LUMO+2 (16 %)	$MLCT_{TAP}$
S_8	22295	449	0.023	HOMO-1→LUMO+4 (58 %)	$MLCT_{TAP}$
S_9	22312	448	0.055	HOMO-1→LUMO+1 (33 %)<br>HOMO-1→LUMO (25 %)	$MLCT_{TAP/dppn2}$
S_{10}	22615	442	0.021	HOMO-1→LUMO+3 (77 %)<br>HOMO-1→LUMO+4 (18 %)	$MLCT_{TAP}$
S_{14}	23848	419	0.051	HOMO-3→LUMO+1 (41 %)<br>HOMO-3→LUMO (30 %)	$LLCT_{dppn2 \rightarrow TAP}$
S_{15}	23878	419	0.057	HOMO-2→LUMO+3 (51 %)<br>HOMO-3→LUMO+1 (11 %)	$MLCT_{TAP}$
S_{16}	24288	412	0.20	HOMO-2→LUMO+5 (46 %)<br>HOMO-2→LUMO+6 (27 %)	$MLCT_{TAP/dppn2}$
S_{17}	25197	397	0.12	HOMO-1→LUMO+5 (46 %)<br>HOMO→LUMO+6 (26 %)	$MLCT_{dppn2}$
S_{18}	25250	396	0.060	HOMO-2→LUMO+5 (77 %)	$MLCT_{dppn2}$
S_{19}	26079	383	0.24	HOMO→LUMO+6 (63 %)	$MLCT_{dppn2}$
S_{20}	27752	360	0.014	HOMO-1→LUMO+6 (89 %)	$MLCT_{dppn2}$
S_{21}	27962	358	0.025	HOMO-2→LUMO+6 (89 %)	$MLCT_{dppn2}$
S_{25}	30629	327	0.026	HOMO-4→LUMO+1 (56 %)<br>HOMO-4→LUMO (29 %)	$LLCT_{dppn2 \rightarrow TAP}$
S_{27}	31178	321	0.032	HOMO-5→LUMO (23 %)<br>HOMO-10→LUMO (19 %)<br>HOMO-10→LUMO+1 (14 %)	LC_{dppn2}/<br>$LLCT_{TAP \rightarrow dppn2}$
S_{28}	31200	321	0.035	HOMO-5→LUMO (35 %)<br>HOMO-12→LUMO (10 %)	LC_{dppn2}/<br>$LLCT_{dppn2 \rightarrow TAP}$
S_{30}	31284	320	0.044	HOMO-5→LUMO+1 (17 %)<br>HOMO-5→LUMO (10 %)<br>HOMO-12→LUMO (10 %)<br>HOMO-10→LUMO+3 (10 %)<br>HOMO-4→LUMO+2 (10 %)	LC_{dppn2}/LC_{TAP}/<br>$LLCT_{dppn2 \rightarrow TAP}$
S_{31}	31343	319	0.051	HOMO-5→LUMO+1 (36 %)<br>HOMO-4→LUMO+2 (16 %)	LC_{dppn2}/<br>$LLCT_{dppn2 \rightarrow TAP}$
S_{35}	31466	318	0.024	HOMO-4→LUMO+2 (57 %)<br>HOMO-5→LUMO+1 (19 %)	LC_{dppn2}/<br>$LLCT_{dppn2 \rightarrow TAP}$
S_{54}	33688	297	0.0094	HOMO-1→LUMO+7 (30 %)	$MLCT_{TAP}$/LC_{TAP}

				HOMO-10→LUMO+3 (21 %)<br>HOMO-10→LUMO (12 %)	
S_{55}	33858	295	0.018	HOMO-1→LUMO+7 (45 %)<br>HOMO-10→LUMO (10 %)	$MLCT_{TAP}$
S_{56}	33910	295	0.027	HOMO-4→LUMO+5 (57 %)<br>HOMO-13→LUMO (10 %)	LC_{dppn2}
S_{61}	34310	292	0.027	HOMO-6→LUMO+3 (35 %)<br>HOMO-7→LUMO+4 (25 %)	LC_{TAP}
S_{62}	34367	291	0.023	HOMO-2→LUMO+7 (48 %)<br>HOMO-7→LUMO+2 (12 %)<br>HOMO-7→LUMO+4 (11 %)	$MLCT_{TAP}$ /LC_{TAP}
S_{64}	34673	288	0.012	HOMO-8→LUMO+2 (25 %)<br>HOMO-2→LUMO+7 (15 %)<br>HOMO-9→LUMO (13 %)	LC_{TAP}
S_{68}	35297	283	0.036	HOMO-2→LUMO+8 (79 %)	$MLCT_{TAP}$
S_{70}	35902	279	0.012	HOMO-8→LUMO+3 (58 %)	LC_{TAP}
S_{73}	36092	277	0.055	HOMO-9→LUMO+4 (32 %)<br>HOMO-2→LUMO+8 (14 %)	LC_{TAP}
S_{74}	36107	277	0.020	HOMO-10→LUMO+2 (16 %)<br>HOMO-11→LUMO (14 %)<br>HOMO-12→LUMO+2 (12 %)<br>HOMO-9→LUMO+4 (11 %)	LC_{TAP}
S_{78}	36592	273	0.052	HOMO-7→LUMO+5 (60 %)<br>HOMO-2→LUMO+8 (11 %)	$LLCT_{TAP\rightarrow dppn2}$
S_{79}	36593	273	0.012	HOMO-7→LUMO+5 (18 %)<br>HOMO-12→LUMO+2 (12 %)	LC_{TAP}/<br>$LLCT_{TAP\rightarrow dppn2}$
S_{82}	36839	272	0.028	HOMO-9→LUMO+3 (22 %)<br>HOMO-6→LUMO+5 (17 %)<br>HOMO-8→LUMO+4 (15 %)	LC_{TAP}/<br>$LLCT_{TAP\rightarrow dppn2}$
S_{86}	37186	269	0.021	HOMO-2→LUMO+8 (25 %)<br>HOMO-9→LUMO+4 (11 %)	$MLCT_{TAP}$ /LC_{TAP}
S_{89}	37717	265	0.48	HOMO-4→LUMO+6 (24 %)<br>HOMO-1→LUMO+9 (17 %)<br>HOMO→LUMO+11 (13 %)	$MLCT_{dppn2}$/<br>LC_{dppn2}/$MLCT_{TAP}$
S_{90}	37840	264	0.085	HOMO-5→LUMO+6 (38 %)	LC_{dppn2}
S_{91}	37861	264	0.012	HOMO-13→LUMO+2 (43 %)<br>HOMO-14→LUMO+4 (38 %)	LC_{TAP}
S_{93}	37985	263	0.014	HOMO-8→LUMO+5 (38 %)<br>HOMO-13→LUMO+2 (13 %)	$LLCT_{TAP\rightarrow dppn2}$
S_{94}	38025	263	0.029	HOMO-8→LUMO+5 (33 %)<br>HOMO-13→LUMO+2 (32 %)	$LLCT_{TAP\rightarrow dppn2}$
S_{95}	38031	263	0.010	HOMO-15→LUMO (42 %)<br>HOMO-15→LUMO+1 (29 %)	LC_{dppn2}
S_{96}	38131	262	0.017	HOMO-9→LUMO+5 (78 %)	$LLCT_{TAP\rightarrow dppn2}$
S_{97}	38227	262	0.022	HOMO→LUMO+10 (58 %)<br>HOMO→LUMO+11 (20 %)	$MLCT_{TAP}$
S_{98}	38353	261	0.046	HOMO→LUMO+12 (70 %)	$MLCT_{TAP}$

S_{100}	38552	259	0.15	HOMO-6→LUMO+6 (56 %)<br>HOMO-1→LUMO+9 (16 %)	$LLCT_{TAP\rightarrow dppn2}$/<br>$MLCT_{TAP}$
T_1	13069	765		HOMO-3→LUMO+4 (96 %)	$LLCT_{dppn2\rightarrow TAP}$
T_2	13483	742		HOMO-3→LUMO+2 (99 %)	$LLCT_{dppn2\rightarrow TAP}$
T_3	14435	693		HOMO-3→LUMO (55 %)<br>HOMO-3→LUMO+1 (36 %)	LC_{dppn2}/$LLCT_{dppn2\rightarrow TAP}$
T_4	14969	668		HOMO-3→LUMO+3 (96 %)<br>HOMO-3→LUMO+5 (96 %)	$LLCT_{dppn2\rightarrow TAP}$
T_5	15892	629		HOMO→LUMO (52 %)<br>HOMO→LUMO+1 (38 %)	$MLCT_{TAP/dppn2}$
T_6	16408	610		HOMO→LUMO+2 (77 %)	$MLCT_{TAP}$
T_7	16648	601		HOMO→LUMO+5 (38 %)<br>HOMO→LUMO+1 (29 %)	$MLCT_{dppn2/TAP}$
T_8	17120	584		HOMO-2→LUMO+2 (27 %)<br>HOMO-1→LUMO (20 %)<br>HOMO-1→LUMO+1 (19 %)	$MLCT_{TAP/dppn2}$
T_9	17721	564		HOMO-1→LUMO+2 (31 %)<br>HOMO-2→LUMO+1 (23 %)	$MLCT_{TAP/dppn2}$
T_{10}	17743	564		HOMO→LUMO+5 (32 %)<br>HOMO→LUMO (26 %)<br>HOMO→LUMO+1 (20 %)	$MLCT_{TAP/dppn2}$
T_{11}	18272	547		HOMO-2→LUMO+2 (27 %)<br>HOMO-1→LUMO (25 %)<br>HOMO-1→LUMO+1 (19 %)<br>HOMO-2→LUMO+4 (16 %)	$MLCT_{TAP/dppn2}$
T_{12}	18284	547		HOMO→LUMO+4 (69 %)<br>HOMO→LUMO+2 (11 %)	$MLCT_{TAP}$
T_{13}	18886	530		HOMO-2→LUMO+5 (63 %)<br>HOMO-2→LUMO+1 (21 %)	$MLCT_{dppn2/TAP}$
T_{14}	18960	527		HOMO-1→LUMO+2 (45 %)<br>HOMO-2→LUMO (24 %)	$MLCT_{TAP}$
T_{15}	19069	524		HOMO→LUMO+3 (77 %)	$MLCT_{TAP}$
T_{16}	19190	521		HOMO-1→LUMO (40 %)<br>HOMO-1→LUMO+5 (24 %)<br>HOMO-1→LUMO+1 (16 %)	$MLCT_{TAP/dppn2}$
T_{17}	19606	510		HOMO-1→LUMO+1 (54 %)<br>HOMO-1→LUMO+4 (96 %)	$MLCT_{dppn2/TAP}$
T_{18}	19882	503		HOMO-3→LUMO+5 (75 %)	LC_{dppn2}

Table S5: Binding constant K_b (Abs) / Binding site size (*s*) determined for **2** with st-DNA using the Bard treatment of UV absorbance at 355 nm (dppp2 transition) and 415 nm (MLCT).

	K_b / s λ_{abs}=355 nm	R^2	K_b / s λ_{abs}=415 nm	R^2
**Δ-1**	Not applicable	NA	1.5 (±0.8) × 10^7 M^{-1} / 2.5 (±0.1)	0.99
**Λ-1**	Not applicable	NA	4.0 (±2.0) × 10^6 M^{-1} / 1.3 (±0.1)	0.99
**Δ-2**	1.5 (±0.7) × 10^7 M^{-1} / 1.5 (±0.03)	0.99	1.4 (±0.8) × 10^7 M^{-1} / 1.4 (±0.04)	0.99
**Λ-2**	7.0 (±1) × 10^6 M^{-1} / 1.4 (±0.02)	0.99	7.2 (±2.5) × 10^6 M^{-1} / 1.4 (±0.04)	0.99

Table S6: DNA binding constants for determined for **1** and **2** with GC and AT DNA, using the Bard treatment of UV absorbance at 355 nm (dppp2 transition) for **1** and 415 nm (MLCT) for **2** and luminescence band at 750 nm for **1** and **2**.

	**Binding constant K_b (Abs) / Binding site size (*s*)**	**Binding constant K_b (Em) / Binding site size (*s*)**	R^2
**Δ-1/GC**	λ_{abs}=415 nm: 1.7(±0.8) × 10^6 M^{-1} / 3.1 (±0.3)	λ_{em}=750 nm: 1.4 (±0.2) × 10^6 M^{-1} / 2.7 (±0.1)	0.99
**Λ-1/GC**	λ_{abs}=415 nm: 1.5 (±0.7) × 10^6 M^{-1} / 3.4 (±0.4)	λ_{em}=750 nm: 2.0 (±0.4) × 10^6 M^{-1} / 6.0 (±0.2)	0.99
**Δ-1/AT**	λ_{abs}=415 nm: 8.7 (±1.2) × 10^6 M^{-1} / 4.5 (±0.1)	λ_{em}=750 nm: 8.5 (±0.5) × 10^6 M^{-1} / 3.8 (±0.1)	0.99
**Λ-1/AT**	λ_{abs}=415 nm: 5.1 (±1.6) × 10^6 M^{-1} / 6.0 (±0.2)	λ_{em}=750 nm: 1.1(±0.3) × 10^7 M^{-1} / 5.0 (±0.1)	0.99
**Δ-2/GC**	λ_{abs}=355 nm: 3.6 (±0.7) × 10^6 M^{-1} / 3.9 (±0.1)	λ_{em}=750 nm: 1.8(±0.4) × 10^6 M^{-1} /3.4 (±0.1)	0.99
**Λ-2/GC**	λ_{abs}=355 nm: 1.2 (±0.2) × 10^6 M^{-1} /4.1 (±0.2)	λ_{em}=750 nm: 3.2(±0.6) × 10^6 M^{-1} /6.0 (±0.1)	0.98
**Δ-2/AT**	λ_{abs}=355 nm: 2.0(±0.3) × 10^6 M^{-1} /4.4(±0.1)	λ_{em}=750 nm: 4.5(±1.3) × 10^6 M^{-1} /4.6(±0.1)	0.99
**Λ-2/AT**	λ_{abs}=355 nm: 5.0 (±0.8) × 10^6 M^{-1} /5.6(±0.1)	λ_{em}=750 nm: 1.4(±0.4) × 10^7 M^{-1} /7.7(±0.1)	0.99

Table S7: Luminescence enhancement for enantiomers of **1** and **2** binding to DNA systems titrated 20 mM phosphate buffer at pH 7.0. I_b= is the intensity when bound to DNA and I_f Intensity of free complex.

	**Enhancement I_b/I_f**		**Enhancement I_b/I_f**
**Λ-1/ST**	3.6 x	**Δ-1/ST**	2.2 x
**Λ-1/GC**	1.8 x	**Δ-1/GC**	1.8 x
**Λ-1/AT**	2.5 x	**Δ-1/AT**	4.3 x
**Λ-2/ST**	2.6 x	**Δ-2/ST**	3.0 x
**Λ-2/GC**	2.0 x	**Δ-2/GC**	2.2 x
**Λ-2/AT**	2.6 x	**Δ-2/AT**	5.4 x

Table S8: DNA binding constants for determined for Λ-**2** with AT step and TA step DNA, using the Bard treatment of UV absorbance at 355 nm (dppp2 transition) and luminescence band at 750 nm.

	K_b / s λ_{abs}=355 nm	R^2	K_b / s λ_{abs}=415 nm	R^2
(5'-CCGG**TA**CCGG)$_2$	2.2 (±0.4) × 10^6 M^{-1} / 3.17 (±0.07)	0.99	1.4 (±0.3) × 10^6 M^{-1} / 7.6(±0.3)	0.99
(5'-CCGG**AT**CCGG)$_2$	3.8 (±1.5) × 10^6 M^{-1} / 3.5(±0.7)	0.99	1.0 (±0.2) × 10^6 M^{-1} / 5.5(±0.3)	0.99

Table S9: Tm of Δ-1 /Λ-1 and Δ-2 / Λ-2 bound to st-DNA ([Nucleobase]:[Os]) in 1 mM phosphate buffer and 2 mM NaCl pH 7.0 taken 0.5 fractional folded (average of 2 runs).

	[Nucleobase]:[Os] = 10:1		[Nucleobase]:[Os] = 20:1		[Nucleobase]:[Os] = 50:1	
Sample	T_m / °C	ΔT_m / °C	T_m / °C	ΔT_m / °C	T_m / °C	ΔT_m / °C
Λ-1	74.3±0.3	13.4±0.5	66.9±0.1	6.0±0.4	63.2±0.1	2.3±0.4
Δ-1	70.2±0.1	9.3±0.4	63.9±0.1	3.0±0.4	62.1±0.6	1.2±0.7
Λ-2	69.4±0.1	8.5±0.4	65.7±0.1	4.8±0.4	63.5±0.6	2.6±0.7
Δ-2	68.8±0.1	7.9±0.4	63.2±0.4	2.3±0.6	63.2±0.1	2.3±0.4

[a] st-DNA (150 μM) in 1 mM phosphate buffer and 2 mM NaCl pH 7.0, T_m = 60.9±0.4.

Optimised geometry coordinates (xyz) for S_0 state of $[Os(TAP)_2(dppz)]^{2+}$ (1)

```
73
dppz gs
  Os  0.04199560937283     -0.06248777038497      0.07127929005792
  N   0.37877814899763     -2.13290310900040      0.12540946047948
  N   1.64665197860402     -0.18565702573692      1.41640257304237
  N   -1.43758719686806     -0.30149155528121      1.52135077711856
  N   -1.66471540017416     -0.12972787465409     -1.13085103030453
  N   -0.03276747534728      2.02324756331575     -0.01442707419826
  N   1.39913015124382      0.30614528423244     -1.46854799819660
  N   3.22329109709394     -5.44719438594375      2.17710494970874
  N   4.58545363251841     -3.38652586715466      3.51393246498253
  C   -0.30149535699087     -3.07641931357848     -0.54154852331021
  H   -1.10828995875951     -2.73853531319851     -1.19294423513018
  C   0.00561142413186     -4.43593078196418     -0.42103797279168
  H   -0.57808606282966     -5.16267228318485     -0.98707360947671
  C   1.04335175294231     -4.83580146386775      0.41077559203357
  H   1.31157384488555     -5.88649047445293      0.52591137041792
  C   1.76420734348956     -3.85883845387212      1.11865511798603
  C   2.87660701142952     -4.18059831168447      2.01542610773142
  C   4.25107161978121     -5.73038765056906      3.00188904928241
  C   4.65669061221096     -7.07990986517015      3.20421352022591
  H   4.11304103429548     -7.86650361677484      2.67746104410659
  C   5.70950056527638     -7.35923084556293      4.04566846061871
  H   6.02371480859314     -8.39379378787089      4.20202765163947
  C   6.40265219726624     -6.31345379373055      4.72119247991827
  H   7.23615827789924     -6.56676413985594      5.38067831112872
  C   6.03557035178703     -4.99790885275807      4.55024207887611
  H   6.55327767355470     -4.18211030789194      5.05824770438568
  C   4.95116023992865     -4.67210377991495      3.68746782266778
  C   3.57412155173174     -3.12387386827452      2.70235977979463
  C   3.16503993739307     -1.73134885822058      2.50657962928062
  C   3.79706790431125     -0.65032364258288      3.14493012791528
  H   4.63468247604665     -0.84146465138044      3.81664520679050
  C   3.33725772311830      0.63732243364132      2.90185279974985
  H   3.79989473875467      1.50282892746217      3.37702407583898
  C   2.25936771014268      0.83549962502038      2.03247303152564
  H   1.87734640920309      1.83625884820782      1.83066179485224
  C   2.09182620025766     -1.45403562240862      1.64367837631178
  C   1.39600599722474     -2.51370636491750      0.94954089169905
  C   -1.33524816810927     -0.40402448728611      2.84673598567362
  H   -0.34610972520221     -0.32381989965366      3.29830842542317
  C   -2.48636322290616     -0.60557047029141      3.64232288562889
  H   -2.36308958102927     -0.68761630193003      4.72658715952443
  N   -3.70846727638409     -0.70157054442631      3.14576662070606
  C   -3.83593923831953     -0.59970125466878      1.80226319953990
  C   -5.12010645499934     -0.68401577143408      1.15795035126481
  H   -5.99390657119968     -0.83668700190120      1.79349787601843
  C   -5.23859301693681     -0.57445611576640     -0.20130359099157
```

H	-6.21073105016638	-0.63553840638953	-0.69346245534708
C	-4.08257869699550	-0.37673700032078	-1.03529002070730
N	-4.18831641939173	-0.26495778006379	-2.37977912164735
C	-3.06749349796045	-0.08760965475994	-3.05931609171931
H	-3.13456446443278	0.00841144649030	-4.14747268876648
C	-1.79197009820486	-0.01851459533449	-2.45309692240392
H	-0.89087056872795	0.11990265897834	-3.05113585399838
C	-2.81688532276416	-0.30109271424139	-0.41479008541693
C	-2.69500111822592	-0.40503028385323	0.99400418736424
C	-0.75948603032882	2.88948798733206	0.69195130153488
H	-1.43115964908990	2.49941907131108	1.45783209424240
C	-0.64854760246130	4.27945427726263	0.45664705018602
H	-1.25926476733835	4.96055862649681	1.05679940926589
N	0.15905332136582	4.80236031065373	-0.45081238889879
C	0.90153836571022	3.94062272529275	-1.18392338259059
C	1.81189566568901	4.40309424283895	-2.19814027098761
H	1.88463753452980	5.48049444377949	-2.35755892332849
C	2.55762890416364	3.52273713822711	-2.93432928145546
H	3.24912389550672	3.87006906381409	-3.70415555294517
C	2.45669681796858	2.10276299046377	-2.72247482487349
N	3.18832781457350	1.22367403493783	-3.44587072081622
C	3.02071071409201	-0.06105526184295	-3.17882911988601
H	3.60473913766233	-0.78656801220964	-3.75325187424695
C	2.12875689654005	-0.54235105643905	-2.19317834292500
H	2.01627917375457	-1.61104728733487	-2.00817821522470
C	1.56638242327656	1.63779803281360	-1.73078676955197
C	0.79716330382481	2.54970779941839	-0.96478114440260

Optimised geometry coordinates (xyz) for T_1 state of $[Os(TAP)_2(dppz)]^{2+}$ (1)

```
73
dppz t1
  Os  0.03382839697998     -0.05353383509860      0.06838999356425
  N   0.36670890085531     -2.12635099332571      0.12054075984007
  N   1.63818488699195     -0.17333816408600      1.41695088568769
  N   -1.44523325904117     -0.29156074970530      1.51771351726216
  N   -1.67171900161684     -0.12547086893245     -1.13409455933360
  N   -0.03516390246273      2.03180268834841     -0.01762848039532
  N   1.39347706146140      0.31072538064245     -1.46890468431616
  N   3.22201222403855     -5.47150633404985      2.17142916471783
  N   4.60212889352619     -3.38076426941619      3.53198131501575
  C   -0.30348987935381     -3.07440286224712     -0.53961069164946
  H   -1.11274853529462     -2.74751004213983     -1.19399443678578
  C   0.01426969585211     -4.43853273438145     -0.41232263057146
  H   -0.56684045690936     -5.16835612849138     -0.97721811868955
  C   1.04931640883261     -4.83125094040226      0.41731314032885
  H   1.32247626156349     -5.88002460288038      0.53572037364147
  C   1.77285310913038     -3.84659198777426      1.12919993783298
  C   2.87102315917313     -4.15651639447016      2.01482911769877
  C   4.25075474000118     -5.73001557941106      2.99686512624967
  C   4.67162923128634     -7.06661142673590      3.20634210051337
  H   4.13527016306653     -7.86006458168043      2.68183096249451
  C   5.77047153040786     -7.38297946047340      4.07721003380441
  H   6.06128845540598     -8.42719869527098      4.20570051281723
  C   6.43509814111023     -6.37838159111367      4.72849751805330
  H   7.27417596013964     -6.59547170057073      5.39212693354950
  C   6.02966351628728     -5.01265032213460      4.53883040855544
  H   6.55175489032587     -4.20359777734297      5.05370499105423
  C   4.95138496610814     -4.66929837523317      3.68617860855183
  C   3.55295817821754     -3.12161519805330      2.68996244878031
  C   3.15390807139228     -1.74605213168622      2.50388409016240
  C   3.79024568879657     -0.66037292946158      3.14936387756672
  H   4.62641445683984     -0.85808627671690      3.82055474262314
  C   3.33632626379657      0.62460261235910      2.91167204699325
  H   3.80093726327847      1.48720578107486      3.39039613350472
  C   2.25484654760611      0.83563809869391      2.03771788717446
  H   1.88120270457260      1.84130313383533      1.84309532359875
  C   2.07581228845698     -1.45044828351862      1.63679876385402
  C   1.38949041565993     -2.49651404813551      0.95011387011085
  C   -1.34305132502284     -0.39129801157290      2.84348950503439
  H   -0.35402084496090     -0.30759388656934      3.29465306936370
  C   -2.49377043389212     -0.59421541158226      3.63905211785564
  H   -2.37053949324620     -0.67401158868176      4.72350480127126
  N   -3.71577047643019     -0.69420497240477      3.14259329475867
  C   -3.84305706195781     -0.59524704885487      1.79886473319271
  C   -5.12682266288520     -0.68367833464682      1.15413764083100
  H   -6.00056037732566     -0.83707610436216      1.78961634877608
  C   -5.24510231579886     -0.57687273276999     -0.20535911429990
```

H	-6.21699610975863	-0.64083243793207	-0.69766811866147
C	-4.08925369694784	-0.37802156452242	-1.03943034727024
N	-4.19479279897157	-0.26859065885506	-2.38411410168784
C	-3.07386718134397	-0.08957858249185	-3.06332224453028
H	-3.14065501213770	0.00454717721075	-4.15167373689136
C	-1.79892471533470	-0.01650874510275	-2.45670524982721
H	-0.89779222083335	0.12297532811750	-3.05446210211403
C	-2.82401106468329	-0.29862175513223	-0.41848751018050
C	-2.70243215641394	-0.39949461613050	0.99051201475393
C	-0.76074442919819	2.90050599217868	0.68719563788133
H	-1.43429575416815	2.51242712412498	1.45246954942140
C	-0.64644087144344	4.28989346696564	0.45099433611700
H	-1.25635114653737	4.97280384122692	1.04993901788846
N	0.16319925808130	4.81054857836292	-0.45614726228786
C	0.90461211976942	3.94649961617159	-1.18757813537065
C	1.81657238627215	4.40597490851122	-2.20181570540210
H	1.89155574061380	5.48305060326970	-2.36242382736294
C	2.56073074187598	3.52329639602190	-2.93681060678152
H	3.25331207387497	3.86841381810714	-3.70667584460152
C	2.45705776154614	2.10370837877929	-2.72332074816023
N	3.18743185223206	1.22235586434584	-3.44521220660314
C	3.01656033003029	-0.06180958092893	-3.17675803937387
H	3.59976695428485	-0.78921563926237	-3.74963479775253
C	2.12219323342037	-0.54011093488844	-2.19205845904103
H	2.00670061947944	-1.60834160515458	-2.00611130267749
C	1.56501102836532	1.64179384021356	-1.73179275576447
C	0.79723061296325	2.55602086822439	-0.96720083436403

Optimised geometry coordinates (xyz) for S_0 state of $[Os(TAP)_2(dppp2)]^{2+}$ (2)

```
72
dppp2 gs
  Os  0.20389738686355     -0.05366083719351     -0.00763985232182
  N   1.96446352505331      0.71923540010490     -0.84510267620711
  N   1.66895136794036     -1.07265310594736      1.09462691521803
  N   0.11640128256592     -1.45316169717813     -1.55227347662287
  N   -1.11528429607189      0.90066231993626     -1.31573040848696
  N   -1.44429895091917     -0.77096144570969      1.05627601785604
  N   0.08962106496163      1.36011859030552      1.52181525223557
  N   6.76659614818788      0.40659201124753     -0.50285953053665
  N   6.45283380192080     -1.45809561743622      1.57519647685850
  C   2.05664087557179      1.61852390109904     -1.83563631949584
  H   1.12206164415030      2.00212802113461     -2.24422639957257
  C   3.29087915257090      2.05795663488536     -2.32506071993376
  H   3.30996189613621      2.79105656217745     -3.13248716614284
  C   4.46558783804239      1.55882805238231     -1.77816091082413
  H   5.44412560941031      1.88221191739514     -2.13362325806738
  C   4.38487093889644      0.61521722735435     -0.73973452514058
  C   5.56404345831580      0.03543342658500     -0.09475508430276
  C   7.83774416086078     -0.13427758060862      0.10569492840388
  C   9.15724930129580      0.22349653176551     -0.28940553203189
  H   9.28962231949702      0.94427200975979     -1.09923517896326
  C   10.21320834315339     -0.35715360638891      0.36757595701443
  H   11.24658343658353     -0.12091121505210      0.10710155914116
  C   9.95060581559200     -1.29070077855911      1.41398718120801
  H   10.79861392662823     -1.75002731819866      1.93504875095863
  N   8.74692350535501     -1.64751872418440      1.80576311280719
  C   7.68197101588402     -1.08893391446537      1.17257191469307
  C   5.40529052695061     -0.92291647997516      0.97031047147220
  C   4.06197644947358     -1.32002971297712      1.39826548768173
  C   3.83103344367991     -2.25183123613512      2.42472394999860
  H   4.68030097053590     -2.70625490522745      2.93517980882338
  C   2.52162346140818     -2.56931996411069      2.76152753501614
  H   2.29807047267925     -3.28583126171844      3.55299201407780
  C   1.46363770640014     -1.96213195747918      2.07723029930025
  H   0.42787286601158     -2.19952940515695      2.32085871043069
  C   2.94983266567349     -0.74895175071751      0.75858532944009
  C   3.11057911236660      0.21735162903713     -0.30400631660407
  C   0.73410868682595     -2.62708229920870     -1.68662955511226
  H   1.38039563275092     -2.96737796038783     -0.87720979631995
  C   0.54566467057150     -3.40931595252814     -2.84954371596120
  H   1.06710772329190     -4.36807001885031     -2.92333458679825
  N   -0.23160354573345     -3.04418741738673     -3.85565938620679
  C   -0.86757356985615     -1.85498091747816     -3.74101271812626
  C   -1.74053973483618     -1.36379126137017     -4.77481558188467
  H   -1.87073627223472     -1.98595947826770     -5.66188767026672
  C   -2.38381173450433     -0.16222232178033     -4.64680673077801
  H   -3.04656640933210      0.21249290953732     -5.42872841824736
```

C	-2.20854782858310	0.65440831657162	-3.47461253320624
N	-2.84038838519717	1.84314038256876	-3.33658866202634
C	-2.61333091889627	2.52011786452166	-2.22312218719837
H	-3.11646930258637	3.48294023206934	-2.09394004114796
C	-1.75247161763088	2.06574144749559	-1.19732430968588
H	-1.58600035282324	2.65813825361968	-0.29698613854868
C	-1.34993279456384	0.18491606218611	-2.45676877269331
C	-0.68663480822957	-1.06136151070355	-2.58741856110903
C	-2.23354745551871	-1.81826955242043	0.81644819106337
H	-2.01668810066724	-2.44260685585226	-0.05095959466516
C	-3.31788346667595	-2.11065732350106	1.67557068945134
H	-3.94432666745798	-2.97888134275420	1.44922484223736
N	-3.61525431798715	-1.39035563180461	2.74435219767530
C	-2.82940053599786	-0.31975593912455	3.00401080380332
C	-3.06331225602635	0.53056160723533	4.14148515936600
H	-3.90087610161425	0.28076451891604	4.79507818188786
C	-2.26190794988837	1.61147283539126	4.39347299960392
H	-2.43395057267624	2.25768205392038	5.25589030960818
C	-1.16156722006866	1.94089951036164	3.52601804768110
N	-0.36798551903965	3.01157595260063	3.76256973518758
C	0.61266386498760	3.23413518510428	2.90321900660835
H	1.26540398846731	4.09465935531668	3.07773717440577
C	0.85878897033870	2.41980725047770	1.77380343985229
H	1.67417052757519	2.64225408517468	1.08543606031879
C	-0.92398047082568	1.11489910668806	2.40618294284088
C	-1.74750839898347	-0.01066286708820	2.15135486101008

Optimised geometry coordinates (xyz) for T_1 state of $[Os(TAP)_2(dppp2)]^{2+}$ (2)

```
72
dppp2 t1
  Os  0.18951861065042     -0.05262160143231     -0.00568851949481
  N   1.94935800849193      0.72324759785187     -0.84381167482547
  N   1.65121940687579     -1.07511888635920      1.10043098487800
  N   0.10644408391020     -1.45251636913460     -1.54907462636300
  N   -1.12369446125655      0.90208608012767     -1.31884319265354
  N   -1.45769998119365     -0.76983474991166      1.05903142617902
  N   0.07591002280913      1.36105429964018      1.52245252827156
  N   6.77183827914490      0.36695556139476     -0.46052076699783
  N   6.46058953502593     -1.45598749366802      1.56680640945285
  C   2.04971265249853      1.62121694924813     -1.82937074989416
  H   1.11967931175721      2.01032316505413     -2.24334588375925
  C   3.29298832298301      2.05806149821385     -2.31345303375914
  H   3.31745796339938      2.79274173708077     -3.11934575996055
  C   4.46127220491410      1.55586917032689     -1.76566250746073
  H   5.44024977925147      1.87990916125996     -2.12135932154037
  C   4.37629180818765      0.60638126361165     -0.72440090073125
  C   5.53676456374369      0.02783045245506     -0.08561946230228
  C   7.87543602192988     -0.12315939938450      0.08785611734888
  C   9.18832251414146      0.21089286971027     -0.28565658906700
  H   9.31828428398147      0.93162192644819     -1.09493980919510
  C   10.28037225146743     -0.36487767930567      0.36108117336186
  H   11.30361488033464     -0.11218226514523      0.07946448038843
  C   10.04561838198217     -1.28020384034491      1.38760681370794
  H   10.86197342911648     -1.76298976599994      1.93299605558662
  N   8.80285520660444     -1.60029939192560      1.74195622116916
  C   7.69741855263620     -1.12151825148501      1.20191091087514
  C   5.38598419169751     -0.92611981779884      0.97132778326091
  C   4.05590946620426     -1.31626868896531      1.39288815799795
  C   3.82250036164920     -2.25367306601955      2.42309741768318
  H   4.67058284556716     -2.71060825994772      2.93376222481756
  C   2.51773409743845     -2.57147229336340      2.76088228491357
  H   2.29721579026610     -3.28955240863085      3.55188410868580
  C   1.45139420708287     -1.96356391592921      2.07937367388003
  H   0.41750881903599     -2.20453954112442      2.32785016764187
  C   2.93234148009756     -0.74535862739864      0.75691725546141
  C   3.09150830635386      0.21274274971028     -0.29435700313481
  C   0.72369191277826     -2.62719710446496     -1.68016302258608
  H   1.36495070864886     -2.96787730674825     -0.86689435024343
  C   0.54095803089516     -3.40898631695850     -2.84414662522381
  H   1.06189468069946     -4.36823600538912     -2.91520064185309
  N   -0.23016151395915     -3.04300548845483     -3.85484687789462
  C   -0.86540687981909     -1.85308243657671     -3.74360158457828
  C   -1.73150110569629     -1.36028648361113     -4.78245781573357
  H   -1.85733685477285     -1.98191247403784     -5.67055335908256
  C   -2.37385603811763     -0.15785117372292     -4.65790700155254
  H   -3.03133145391185      0.21800863721334     -5.44375308611466
```

C	-2.20458652343492	0.65820917972375	-3.48436844872640
N	-2.83600254714996	1.84755051237556	-3.34959646951918
C	-2.61481791005498	2.52359220616180	-2.23422025637963
H	-3.11764675272993	3.48691435326973	-2.10753766761294
C	-1.76059935337683	2.06777067997566	-1.20369741645834
H	-1.59832650425153	2.65962585462162	-0.30222827086615
C	-1.35241859904111	0.18733153339020	-2.46176844952347
C	-0.69030658187229	-1.05989376193107	-2.58885773077993
C	-2.24728376931389	-1.81721945133022	0.82006116021678
H	-2.03068125326920	-2.44163334545250	-0.04732518377890
C	-3.33067047995100	-2.10970952026006	1.68012334590733
H	-3.95723823133207	-2.97801181237146	1.45438379558036
N	-3.62717886729293	-1.38955498360359	2.74941658708746
C	-2.84104549088606	-0.31897422394715	3.00830347771151
C	-3.07366706304563	0.53137231250951	4.14607573336259
H	-3.91044565938682	0.28155408967523	4.80068093270651
C	-2.27207986573074	1.61238996855188	4.39712247866925
H	-2.44330949362221	2.25854628471573	5.25975360516047
C	-1.17273989945922	1.94203787853064	3.52839559172263
N	-0.37904473261243	3.01287063992203	3.76389021378812
C	0.60064207404816	3.23514384567064	2.90314016488978
H	1.25359976301827	4.09568618442240	3.07683344343230
C	0.84549628480959	2.42079923229312	1.77360983505640
H	1.66004513004366	2.64270145937591	1.08407651249937
C	-0.93644787723640	1.11592562657095	2.40841802279700
C	-1.76002248239556	-0.00976675896835	2.15461296349716

References

1. Rigaku Oxford Diffraction, 2020. *CrysAlisPro Software System*, Version 171.41.93a, Rigaku Corporation, Oxford, UK.

2. SHELXL version 3/2018, G. M. Sheldrick, 2015, *Acta Cryst.*, C**71**, 3-8.

3. O. V. Dolomanov, L. J. Bourhis, R. J. Gildea, J. A. K. Howard and H. Puschmann, *J. Appl. Cryst.* 2009, **42**, 339–341.

4. M. T. Carter, M. Rodriguez and A. J. Bard, *J. Am. Chem. Soc.*, 1989, **111**, 8901–8911.
